# Photochromic coenzyme Q derivatives: switching redox potentials with light†
†Electronic supplementary information (ESI) available: Detailed experimental procedures and analytical data are available. CCDC 1533545. For ESI and crystallographic data in CIF or other electronic format see DOI: 10.1039/c7sc00781g



**DOI:** 10.1039/c7sc00781g

**Published:** 2017-07-20

**Authors:** Nadja A. Simeth, Andrea C. Kneuttinger, Reinhard Sterner, Burkhard König

**Affiliations:** a University of Regensburg , Faculty of Chemistry and Pharmacy , Institute of Organic Chemistry , Universitätsstraße 31 , 93053 Regensburg , Germany . Email: burkhard.koenig@ur.de ; Tel: +49-941-943-4575; b University of Regensburg , Faculty of Biology and Preclinical Medicine , Institute of Biophysics and Physical Biochemistry , Universitätsstraße 31 , 93053 Regensburg , Germany . Email: reinhard.sterner@ur.de

## Abstract

Coenzyme Q is an important redox cofactor involved in a variety of cellular processes, and is thus found in several cell compartments. We report a photochromic derivative of coenzyme Q that combines the molecular structures of the redox active cofactor and a photochromic dye. Light irradiation triggers an electronic rearrangement reversibly changing the redox potential. We used this effect to control the intermolecular redox reaction of the photochromic coenzyme Q derivative with dihydropyridine in solution by light irradiation. On mitochondria, the altered redox properties showed an effect on the respiratory chain. The experiments demonstrate that the redox reactions can be initiated inside the system of interest through irradiation with light and the accompanied photoisomerization.

## Abstract 2

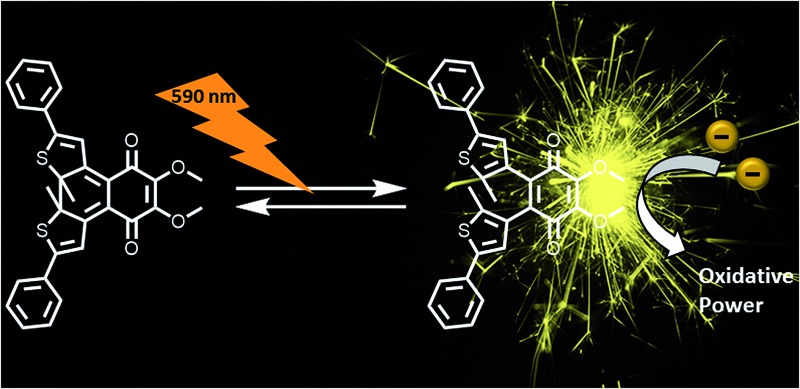
A photochromic coenzyme Q derivative could be activated through irradiation with orange light and initiate redox reactions with Hantzsch ester and on isolated mitochondria.

## 1. Introduction

The concept of employing small, light-responsive molecules to gain spatiotemporal control over biological processes has been known for a long time.^1^ However, in recent years, the field, now often termed as photopharmacology, expanded rapidly.^2–5^ Photochromic inhibitors based on azo dyes or bridged diarylethenes (DAEs) were used to modulate enzymatic activities.^6–11^ Moreover, switchable antibiotics^12–14^ and molecules, which cause light-induced apoptosis of single cells in tissue^15^ illustrate the potential of the approach. Using light as an external stimulant adds an extrinsic parameter determining the selectivity of the compound.^3^ Furthermore, light is non-invasive, easy to apply, and offers an inimitable spatiotemporal control over the process of interest.^1^ Both azobenzenes and DAEs interconvert upon light irradiation between two structural isomers, which differ strongly in their physical properties, and thus also in their activity towards biomolecules.^1,2^ DAEs generally show good photoconversion, fatigue resistance, and thermal stability. They are thus frequently used as photochromic tools in biological contexts.^1,16^

While several analogues of DNA bases,^17,18^ fatty acids, and phospholipids^19–23^ have been reported, the number of photoswitchable derivatives of enzymatic cofactors such as ATP, NAD(P)H, FAD, CoQ, is small. Recently, Wilson *et al.* presented a photochromic analogue of pyridoxal-5′-phosphate,^24^ and furthermore ATP has been derivatized into a light-controllable mimetic.^25^ However, redox cofactors, a likewise widely spread family of coenzymes, have not been considered so far.^26^ They are involved in many cellular processes, and therefore found in various cellular compartments. They facilitate *e.g.* transhydrogenation reactions catalyzed by oxidoreductases by either accepting or providing electrons.^27^ In general, they act as an activated electron carrier for the oxidation of metabolites in aerobic organisms. An important auxiliary molecule involved in this process is Coenzyme Q (CoQ), also called ubiquinone. The core part of the molecule, the benzoquinone ring, facilitates the molecule to serve as a two-electron carrier – interconverting between a ubiquinone and an ubiquinol form. Due to its large, nonpolar poly-isoprenyl chain, the coenzyme is able to diffuse within lipophilic membranes.^28^ Besides its function as an auxiliary in metabolism,^26^ it also acts as an antioxidant,^29,30^ regulates the physiochemical properties of membranes,^31^ and modulates the amount of β-integrins on the surface of blood monocytes.^32^

As most of the cofactors, redox cofactors are not only present in one single cellular compartment, but widely distributed. Thus, targeting them with conventional strategies of medicinal chemistry would cause an uncontrollable impact on all cells of the organism and it would be difficult to regulate the effect in the desired fashion. However, using a photopharmacological strategy, we are able to activate the agent specifically at the favored site for a defined time.

For our CoQ mimetic, we merged a redox-active benzoquinone moiety with two substituted thiophene moieties, building a photochromic DAE. Through irradiation with light, the 6π-system formed by the three parts undergoes an electrocyclic ring-closing reaction. The ring-closure changes color, shape, and electronic properties of the molecule. Most applications of photochromic compounds aim at an altered structure with different physiological effect, but only few studies utilized the different electronic properties of both photoisomeres.^24,33^ However, we focus on changing the redox potentials of the benzoquinone moiety through electronical changes upon irradiation. This specific mode of action may allow in principle to use one cofactor mimetic to target all related enzymes even if the active site differs, as the molecule's mode of action does not rely on its spatial interaction, but its redox state. Moreover, benzoquinone motifs have been considered as a scaffold by Deng *et al.* and Katsumura *et al.* and were investigated regarding their switching behavior.^34,35^

Light- and electronic-modulated switches have already been a subject of physical–electrochemical as well as spectroscopic studies,^36–41^ but to the best of our knowledge, have never been considered as a the role of a cofactor in biological context. Thus, we present the first photochromic molecule that has the potential to serve as a redox cofactor.

## 2. Results and discussion

### 2.1. Design

**Fig. 1:**
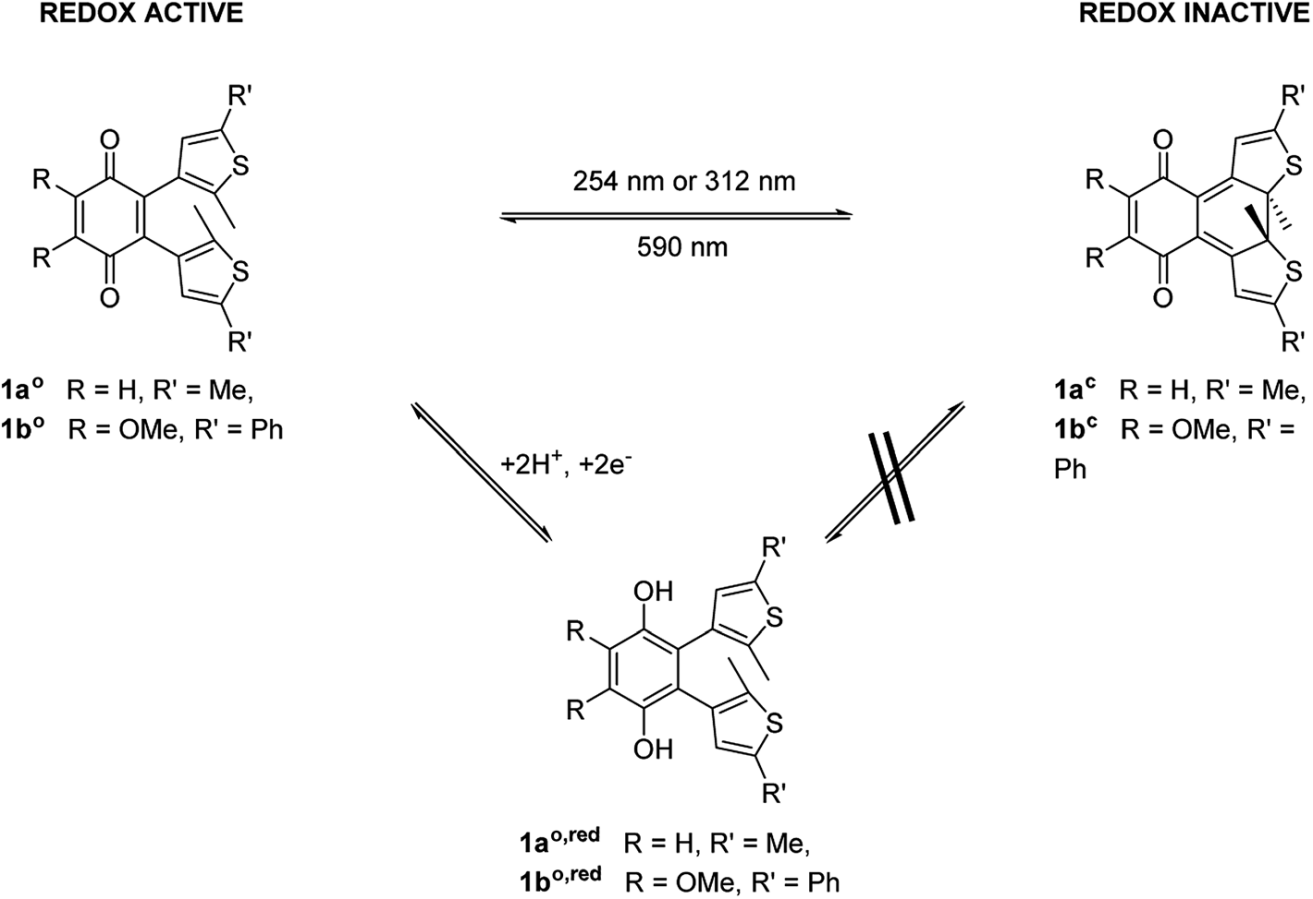
General principle of switchable redox potentials based on photochromic CoQ mimetics.

To modulate the redox properties of coenzyme Q by light irradiation, we designed the photochromic derivatives **1a^o^** and **1b^o^** as depicted in Fig. 1 by merging a redox-active benzoquinone with thiophene moieties to build a DAE. This allows the molecule to accept electrons for redox reactions just in one photoisomeric state. Upon irradiation by light of 254 nm, the redox-switch undergoes a 6π-conrotatory electrocyclic ring closure to the closed photoisomer (**1a^o^**, **1b^o^** to **1a^c^**, **1b^c^**, Fig. 1). The rearrangement delocalizes one double bond of the redox-active moiety in the conjugated backbone of the closed switch. The “closed photoisomer” should be thus mostly redox-inactive as the quinone system was intercepted. In contrast, the “open form” should be able to undergo oxidation reactions similar to unaltered CoQ (**1a^o^**, **1b^o^** to **1a^o,red^**, **1b^o,red^**, Fig. 1). The methoxy groups of the benzoquinone moiety (**1b^o^**) are known to be crucial for interaction at the binding site of CoQ-consuming proteins, and are therefore retained in the model compound (Fig. 1).^42^

### 2.2. Synthesis of the photochromic coenzyme Q derivatives

**Scheme 1:**
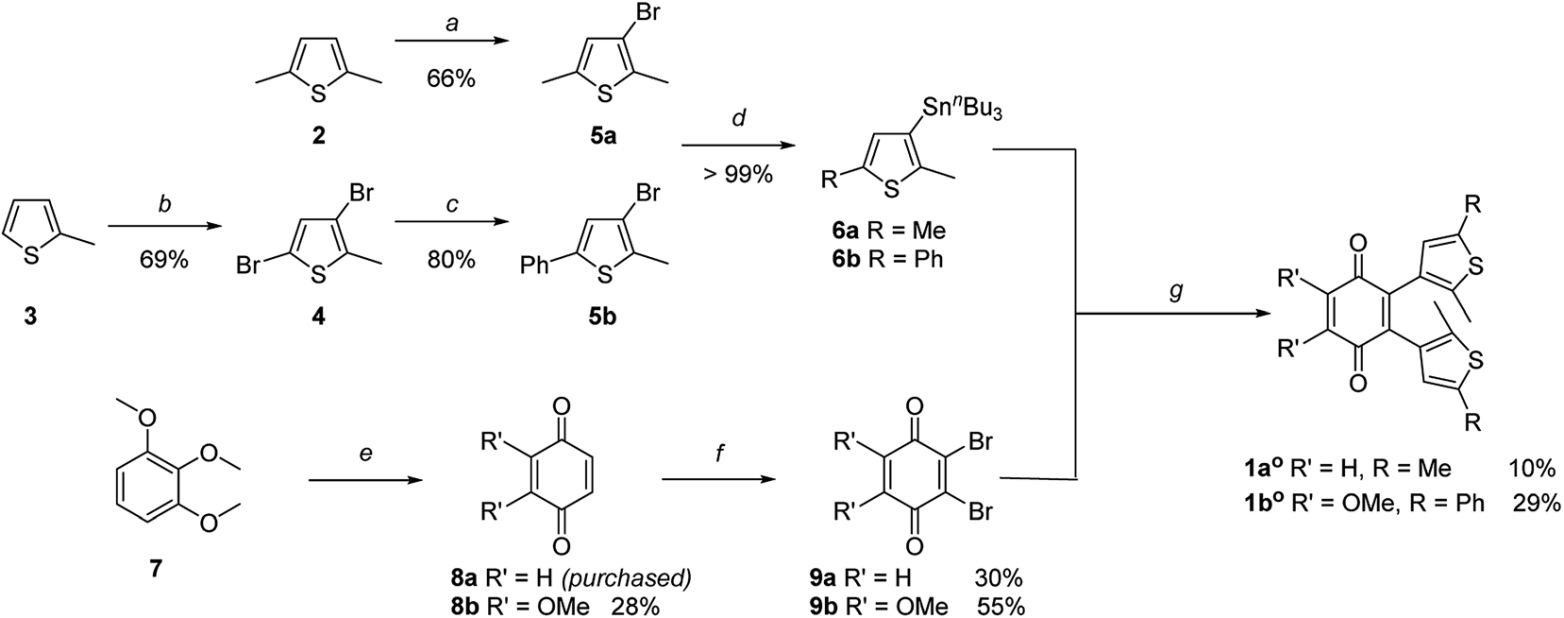
Synthesis of photochromic coenzyme Q mimetics **1a^o^** and **1b^o^**: (a) NBS, AcOH, 2 h, 66%. (b) Br_2_, AcOH, 18 h, r.t., 69%. (c) PhB(OH)_2_, Pd(PPh_3_)_2_Cl_2_, AsPh_3_, Na_2_CO_3_ (2 M, aq.), THF, 16 h, 80 °C, 80%. (d) ^*sec*^BuLi, SnBu_3_Cl, dry THF, –78 °C to r.t., quantitative. (e) K_3_[Fe(CN)_6_], H_2_O_2_, AcOH, r.t., 28%. (f) Br_2_, H_2_SO_4_, Et_2_O, 1 h; then Ag_2_O, Et_2_O, 1 h, 30% for **9a**; 55% for **9b**. (g) Pd(PPh_3_)_4_, CuI, dry toluene, 85 °C (**1a^o^**) or 115 °C (**1b^o^**), 16 h, 10% (**1a^o^**) or 29% (**1b^o^**).

The key step of the synthesis towards compounds **1a^o^** and **1b^o^** was a Stille cross-coupling reaction of **5a** or **5b** onto quinone **8a** or **8b**, respectively (Scheme 1). The thiophene precursors (**5a** and **5b**) were easily synthesized from 2,5-dimethyl thiophene (**2**) or 2-methyl thiophene (**3**), respectively, partly following known protocols.^34,43^ After bromination and arylation through Suzuki–Miyaura cross-coupling the pre-functionalized thiophenes **5a** and **5b** were treated with ^*sec*^BuLi and subsequently SnClBu_3_ yielding organo stannates **6a** and **6b**.^35^ The quinone **8b** was synthesized through oxidation of **7** using Fenton's reagent in acetic acid.^44^ Benzoquinone (**8a**) as well as 2,3-dimethoxy benzoquinone (**8b**) were then halogenated utilizing bromine under oxidative conditions.^45^ The final step was performed employing Pd(PPh_3_)_4_ and elevated temperatures for 16 h to give **1a^o^** and **1b^o^** in acceptable yields (10% or 29%, respectively).^34^

**Scheme 2:**
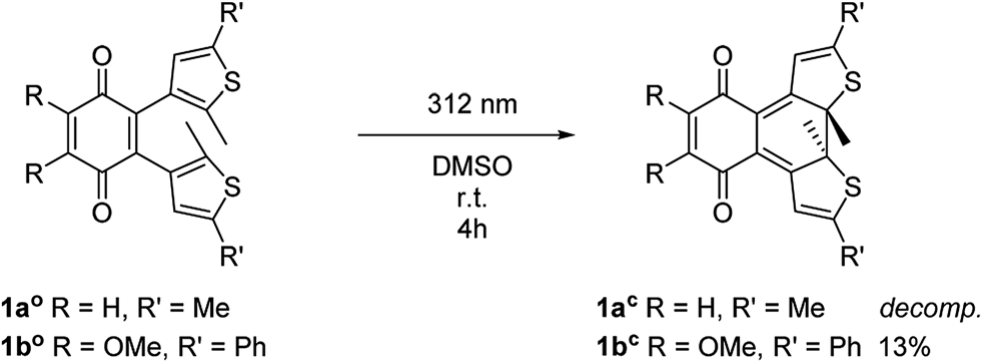
Synthesis of closed isomers **1a^c^** and **1b^c^**.

The open form of the photochromic quinones (**1a^o^** and **1b^o^**) were converted into the corresponding closed isomers (**1a^c^** and **1b^c^**) through irradiation with UV light (Scheme 2). Compound **1a^o^** did not tolerate the long-term irradiation and decomposed. However, closed isomer **1b^c^** was isolated from reversed phase column chromatography (13% yield).

### 2.3. Spectroscopic, electrochemical and spectroelectrochemical studies

**Fig. 2:**
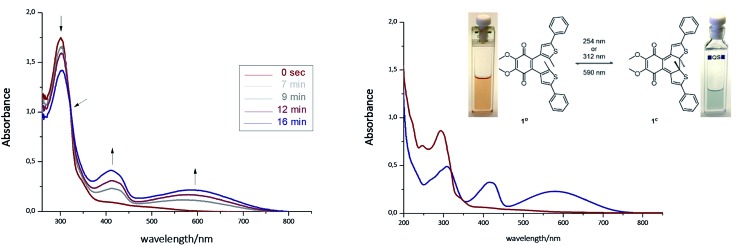
Left: Photoisomerization of **1b^o^** in DMSO (30 μM, irradiation with 312 nm). Right: Spectra of isolated photoisomers **1b^o^** (red line, maxima at 247 nm and 297 nm) and **1b^c^** (blue line, maxima at 307 nm, 415 nm and 583 nm) in MeCN (isosbestic point at 317 nm). For further details see ESI.†

The photoisomerization of compound **1a^o^** and **1b^o^** was investigated in toluene, dimethylsulfoxide (DMSO), dichloromethane (DCM), dioxane and MeCN. Unfortunately, compound **1a^o^** did not tolerate irradiation with UV light (312 nm) and resulted in decomposition into uncharacterized products (for spectra see ESI†). However, compound **1b^o^** switches in polar and non-polar solvents (Fig. 2 and ESI†). Upon irradiation with UV light (312 nm or 254 nm, respectively), the color of the solution changed from bright red to blue. The photoisomerization was monitored by UV/Vis spectroscopy; associated spectra in DMSO are depicted exemplary in Fig. 2 (further spectra are shown in the ESI†). New absorption maxima at 410 nm and 594 nm show the formation of the closed photoisomer; the isosbestic point at 323 nm indicates a distinct two-compound isomerization. Photostationary states (PSSs) were determined by HPLC measurements (ESI†). The PSS for the closing reaction was determined to be 52% in DMSO, whereas the opening reaction was achieved quantitatively through irradiation with orange light (590 nm, 30 μM, 2 min). An intramolecular electron transfer (TICT) as reported for other DAE derivatives bearing an electron-acceptor core moiety rationalizes the generally rather low PSS for the closing reaction.^46,47^ Thus, we focused on the opening reaction for further experiments, which generates a redox active compound from a redox silent molecule upon irradiation.

**Chart 1:**
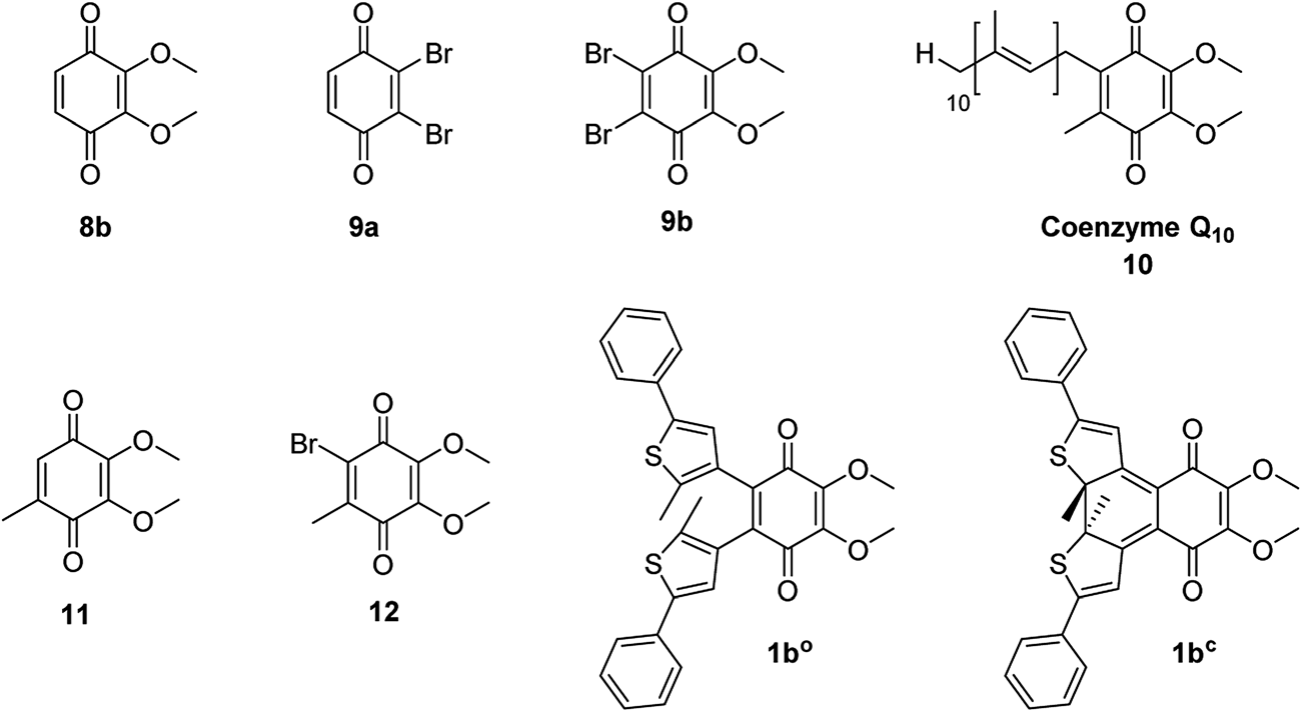
1,4-Benzoquinones included in the electrochemical studies.

**Fig. 3:**
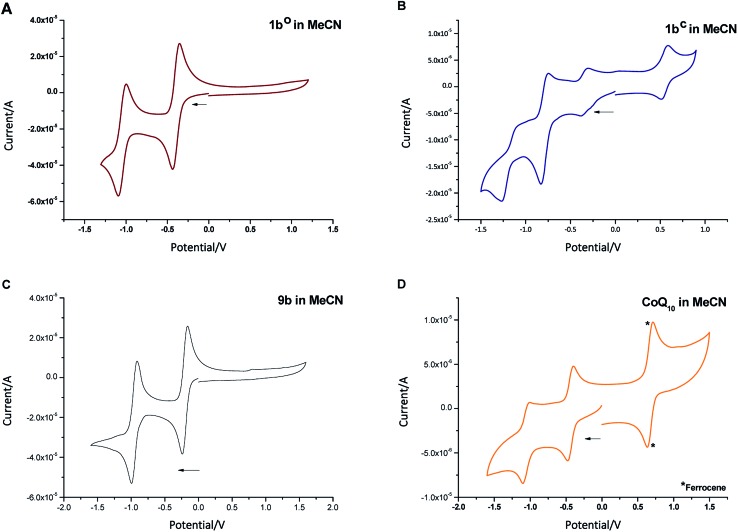
Cyclic voltammogram of compounds **1b^o^** ((A) 30 μM), **1b^c^** ((B) 2 μM), **9b** ((C) 30 μM), and CoQ_10_ ((D) 2 μM, *ferrocene (0.005 M) as internal standard) in MeCN + 0.1 M Bu_4_NBF_4_.

To determine the redox potential of CoQ derivatives **1b^o^** and **1b^c^**, we performed cyclic voltammetric (CV) studies. The compounds included in the study are depicted in Chart 1. Compound **12** was hereby synthesized from commercially available **11** similar to **9b** in one step. All 1,4-benzoquinone derivatives (**9b**, **10**: Fig. 3C and D; **8b**, **9a**, **11**, **12**, ESI†) including the open photochromic compound **1b^o^** (Fig. 3A) show similar behavior as CoQ_10_ (Fig. 3D). Specifically, all compounds exhibit two well-defined, fully reversible reduction signals. This finding is in agreement with previous investigations on benzoquinones. The first step corresponds to the formation of a semiquinone radical, while the second step corresponds to the formation of a dianion (or hydroquinone if protons are present).^48^ The potentials of the molecules are predestined by a substitution pattern of the respective compound and are summarized in Table 1. The substituents of the benzoquinone core affect the reduction potential values. With an increasing number of electron withdrawing bromine substituents the reductions occur at less negative potentials. In contrast to all other investigated *para*-benzoquinones, the closed isomer **1b^c^** generates three – not two – cathodic waves as well as one anodic one (Fig. 3B), which are reproducible (*cf.* ESI†). Also, the intensities of the respective waves differ, and hence indicate that redox steps involve a different number of electrons.

**Table 1:** Redox potentials of benzoquinone derivatives **8b**, **9a**, **9b**, **1b^o^**, **1b^c^**, **CoQ_10_ (10)**, **11**, and **12** (*cf*. Chart 1) *vs*. SCN^49^ (determined by CV measurements in MeCN + 0.1 M Bu_4_NBF_4_ using ferrocene (0.005 M) as internal standard)

Compound	Potentials *vs.* SCN^49^
**1b^o^**	–0.58; –1.23
**1b^c^**	+0.83; –0.24; –0.55; –1.39
**8b**	–0.66; –1.37
**9a**	–0.24; –0.97
**9b**	–0.35; –1.1
**CoQ_10_ (10)**	–0.77; –1.39
**11**	–0.69; –1.34
**12**	–0.54; –1.27

Next, we investigated the redox states by spectroelectrochemistry, using several different 1,4-benzoquinone derivatives for comparison (Chart 1).

**Fig. 4:**
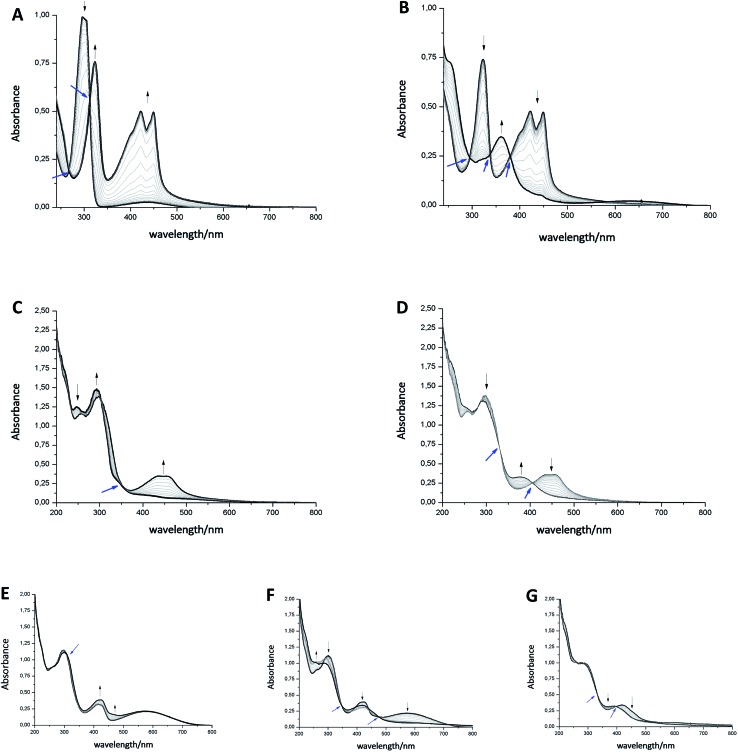
Spectroelectrochemical studies of compounds **9b** ((A) first peak, (B) second peak), **1b^o^** ((C) first peak, (D) second peak) and **1b^c^** ((E) first peak, (F) second peak, (G) third peak). One spectra-set collerates with one cathodic wave. Black arrows indicate increase/decrease of maxima; blue arrows assign isosbestic points.

Exemplary, changes of the UV/Vis spectra of compounds **1b^o^**, **1b^c^**, and **9b** during one cathodic potential sweep are depicted in Fig. 4 (difference spectra of **1b^o^**, **1b^c^**, and **9b** as well as UV/Vis spectra of **9a**, **10**, **11** and **12** can be found in ESI†). All *para*-benzoquinones – besides the closed photoswitch **1b^c^** – show similar absorption bands with the changing potential; without applying an external potential, the benzoquinones show an absorption band at around 280–300 nm depending on their substitution pattern. During the first reduction step corresponding to the formation of the semiquinone intermediate,^48^ this band decreases to give rise to two new bands – one at around 310 nm and a broader one at around 410 nm exhibiting a well-defined double maximum, which is also present in the case of **1b^o^** and CoQ_10_, but not so well-defined.^50^ For CoQ_10_, this might be due to solubility issues in MeCN. The second cathodic wave is the formation of the dianion,^48^ generating a novel band at around 370 nm, whereas the two bands of the semiquinone radical decrease again. All spectra show clear isosbestic points indicating a distinct two-compound process. The reduction process is fully reversible and can be repeated over several cycles.

In contrast to the other investigated benzoquinones, the closed photoswitch **1b^c^** showed a slightly different spectrum in the beginning due to the attached chromophore. The molecule exhibits maxima at 307 nm, 415 nm, and 583 nm. During the first reduction step, the maximum at 415 nm broadens, while the other maxima remain unchanged. Changes are more drastic as the second reduction occurs; the long-wave band around 580 nm fully decreases, the band at around 410 nm broadens even more and the short-wave band at around 300 nm experiences a hypsochromic shift. While the last reduction step takes place, the band around 410 nm is also hypsochromically shifted.

Although there are similarities to the non-photochromic benzoquinones and to **1b^o^**, the spectroelectrochemical studies clearly reveals a different behavior of **1b^c^**. Especially, the well-pronounced decrease of the long-wave band around 580 nm, which can be assigned to the conjugated thiophene backbone in the chromophore of the photoswitch,^16^ indicates a redox process in this moiety of the molecule. However, this appears rather counterintuitive, as though impaired the benzoquinone moiety still should be more prompt to reduction. Yet very recently, Saito *et al.* report EPR and computational studies on a related molecule and could show the formation of a radical anion similar to a semiquinone.^51^ A charge delocalization over the whole molecule is likely to be allowed through two nearly degenerated LUMO and LUMO+1. Thus, we suggest that the second reductive wave also has semiquinone character but delocalized over the whole molecule (loss of long-wave band around 580 nm, which is associated with the closed ring in DAEs). The first and the third reduction potential show less prominent changes in UV/Vis and are yet to be assigned.

Overall, the cyclovoltammetric and spectroelectrochemical studies revealed a different redox behavior for both photoisomers of **1b**. Spectroscopic investigations show that **1b^o^** and **1b^c^** can be in fact interconverted into each another through light, but the PSS values of the closing reaction in all investigated solvents (see ESI†) is rather low (maximum 52% in DMSO). In addition, the time required for the opening and the closing reaction are in the minute-range. However, both characteristics are crucial for many redox mediated reactions, especially in biological systems as *e.g.* enzymatic reactions take place in the milli-second range or even faster.^52^ Therefore, compound **1b** can only be considered for a limited set of experiments up to now. The investigated redox reactions should be slow or independent of diffusion of the cofactor mimetic during irradiation. The quantitative opening reaction can be used to provide a redox partner within a system upon irradiation similar to caged compounds. However, the reverse process would not be quantitative. Considering these properties, we investigated the applicability of the redox switch as a tool in reactions.

**Fig. 5:**
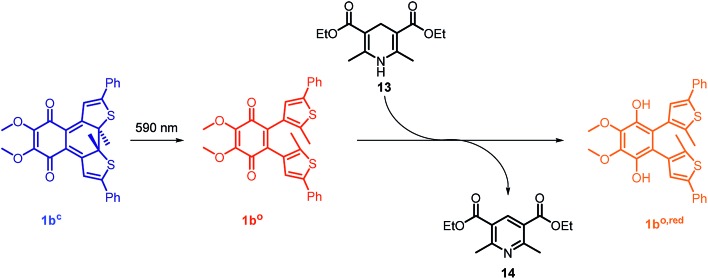
Photoactivated redox reaction between photochromic CoQ derivative **1b^o^** and an NADH model Hantzsch ester **13**. Compound **1b^o^** is generated *in situ* from closed isomer **1b^c^** *via* irradiation.

### 2.4. Photoactivated oxidation reactions

In the mitochondrial respiratory chain, CoQ_*n*_ oxidizes NADH to NAD^+^ in complex I (NADH-Q oxidoreductase).^26^ Thus, we decided to let our photochromic CoQ mimetic **1b^o^** react with Hantzsch ester **13**, which belongs to the same family of dihydropyridines as NADH, and hence serves as an excellent test substrate for a photoactivated oxidation reaction (Fig. 5).

**Fig. 6:**
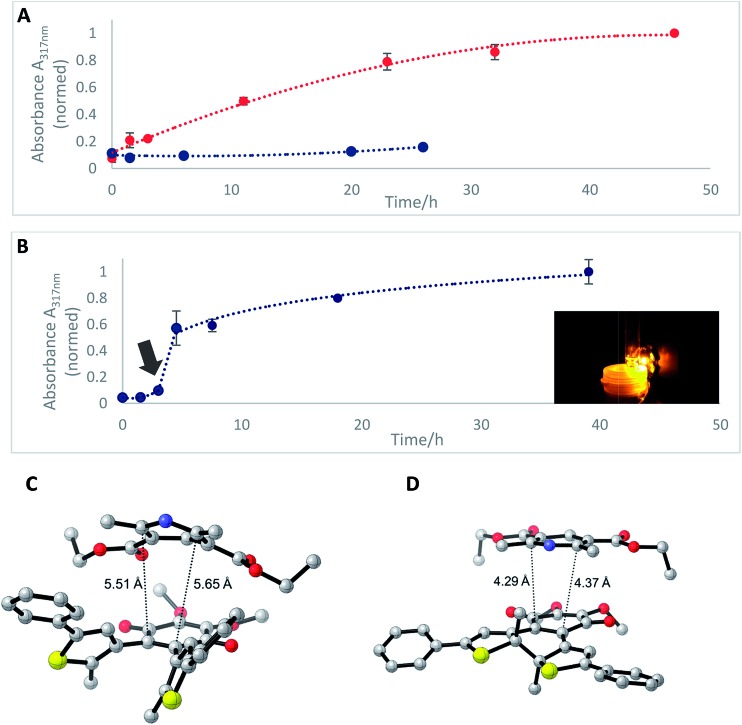
(A) Formation of **1b^o,red^** *via* reduction of **1b^o^** (5 mM, DMSO, red line) or **1b^c^** (5 mM DMSO, blue line) through dihydropyridine derivative, Hantzsch ester **13** monitored through HPLC-UV/Vis. (B) Formation of **1b^o,red^** after photoactivation (arrow) of **1b^c^** (10 mM, DMSO) after three hours of initial reaction time with a 590 nm single-spot LED for 10 min (picture). Values are typically the means ± SEM of three individual experiments. (C) Optimized geometry of the **1b^o^**–**13** adduct. (D) Optimized geometry of the **1b^c^**–**13** adduct (all the distances are reported in Å).

A solution of each photoisomer (5 mM, **1b^o^**, **1b^c^**) was treated with a solution of Hantzsch ester **13** (12 mM) in DMSO and reacted at ambient temperature in the dark. We took samples in certain time intervals and analyzed them through analytical HPLC (UV/Vis detection at 317 nm which equals PSS, HPLC traces see ESI†) to follow the consumption and the generation of the respective redox pairs. The formation of **1b^o,red^** from **1b^o^** and **1b^c^**, respectively, through reduction with **13** is depicted in Fig. 6A (normed areas of HPLC traces). The open photoisomer **1b^o^** is rapidly reduced by Hantzsch ester **13** (red line, Fig. 6A), whereas the closed one **1b^c^** shows almost no conversion to **1b^o,red^** or any other product (blue, Fig. 6B, minor background reaction results from thermal ring-opening). Thus, compound **1b^c^** is unreactive before irradiation.

Then, we investigated whether the formation of **1b^o,red^** can be triggered through light-activation of **1b^c^**. The closed isomer (10 mM, DMSO) was treated with Hantzsch ester **13** (12 mM) and left stirring in the dark for three hours. Subsequently, the solution was irradiated with a 590 nm single-spot LED for 10 min (picture, Fig. 6) to be quantitatively converted into **1b^o^**. HPLC analysis confirmed a full conversion into only the open photoisomer and also an immediately initialized reduction into **1b^o,red^** (Fig. 6B, for HPLC traces see ESI†). The initial slope of the reaction seems to be larger than utilizing **1b^o^**. This could be due to a pre-aggregation of **1b^c^** and the Hantzsch ester. A preliminary computational analysis was carried out in order to unravel the presence of an interaction between both photoisomers and Hantzsch ester **13**. The DFT method at the B3LYP/6-31G(d) level of theory (*cf.* ESI†).^53–56^ We decided to follow a modification of an approach already found in the literature^57^ to gain qualitative insight on the presence of a non-covalent π stacking between the two photoisomers of **1b** and **13**.

From an energetic point of view, the closed form appeared to be more stable (*ca.* 7 kcal mol^–1^), *cf.* ESI† due to the interaction with **13**. Moreover, the geometry of the optimized structures clearly shows the Hantzsch ester resides in closer proximity to **1b^o^** than to **1b^c^** (see Fig. 6C and D).

In summary, we can conclude that the intermolecular reaction does relay on the respective redox potentials of the compounds indicating that we are truly able to change redox potentials of **1b** by photo-induced ring-opening of the electronically caged closed photoisomer.

### 2.5. Photoactivated mitochondrial reduction of 2,6-dichlorophenol indophenol

**Fig. 7:**
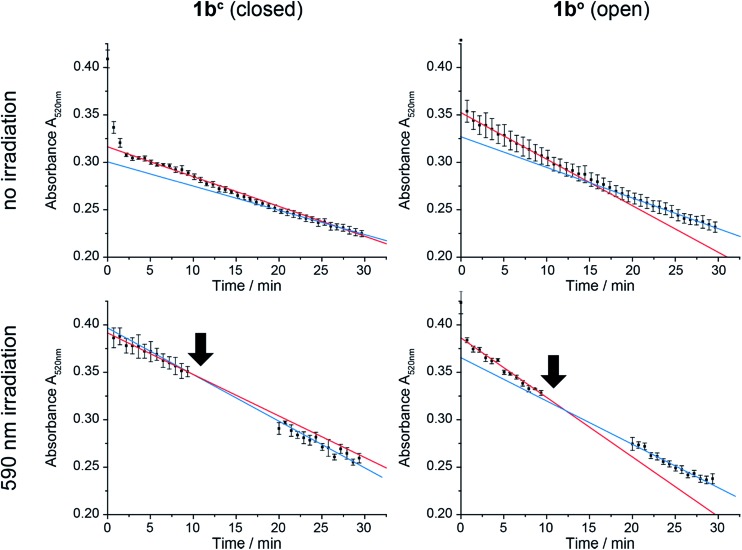
Reduction of 2,6-dichlorophenol indophenol (DCIP) by mitochondria over time monitored spectrophotometrically at 520 nm. **1b^c^** and **1b^o^** assays were carried out continuously (upper panels) or interrupted by irradiation at 590 nm as indicated by arrows (lower panels). The data were fitted for linear activity at 0–10 min (red) and 20–30 min (blue). Whereas activity decreases with time without irradiation of either the closed or the open form as well as after irradiation of the open form (blue slope < red slope), it increases after irradiation of the closed form (blue slope > red slope).

In order to test the effect of **1b^c^** and **1b^o^** on a natural system, mitochondria were isolated from the wildtype yeast strain BY4742.^58^ CoQ_*n*_ serves as electron transmitter in the respiratory chain of mitochondria from complex II (succinate dehydrogenase) to complex III (cytochrome-C oxidoreductase). By using 2,6-dichlorophenol indophenol (DCIP) as electron acceptor instead of Complex III, CoQ analogues can be tested on isolated mitochondria.^59^ The reduction of DCIP with **1b^c^** and **1b^o^** was followed spectrophotometrically (Fig. 7), continuously for 30 min or interrupted by irradiation with 590 nm, in order to switch the closed **1b^c^** into the open, more active **1b^o^** isomer.

For both isomers of our CoQ mimetic **1b**, reduction of DCIP could be observed in the continuous assay (Fig. 7, no irradiation) where a fast initial rate (red) was followed by a lower rate (blue). Comparing the initial rate of DCIP reduction with **1b^c^** and **1b^o^**, the open isomer is only approximately 70% more active than the closed isomer. We assume that this seemingly high activity of the closed isomer is due to residual activity of bound natural CoQ_*n*_, which we cannot exclude in isolated mitochondria.

To emphasize the activity difference of **1b^c^** and **1b^o^**, we irradiated both isomers after 10 min reaction time (Fig. 7, 590 nm irradiation). The activity for the open isomer decreased as in the non-irradiated assay (blue slope < red slope). However, the rate for the closed isomer after irradiation increased (blue slope > red slope) indicating that the reaction was restarted.

Thus, despite the complex system of mitochondria, in which CoQ is deployed in several reactions, we were able to see an activation of the DCIP reduction after irradiation of **1b^c^**. Consequently, the photochromic CoQ mimetic **1b** cannot only replace CoQ_*n*_ in a natural system, but can also activate the same after irradiation.

## 3. Conclusion

In conclusion, we have developed a photochromic CoQ derivative (**1b**) combining a redox active and a photochromic moiety in one molecule. We could show by cyclic voltammetry that the redox potential of both photoisomers differs, and thus can be altered through photoisomerization. In addition, the mode of action of cathodic waves in spectroelectrochemical studies was examined indicating the nature of the reductive steps. Furthermore, we could show that the closed form of the switch (**1b^c^**) is redox inactive in a reaction with Hantzsch ester, a member of the dihydropyridine family, and has to be photoactivated to its open isomer (**1b^o^**) to be redox active. This proves that the reactivity of **1b** relies on the altered redox potentials of the two photoisomers, and is thus controllable through electronic changes rather than by conformational and geometry changes as seen in most other studies. Finally, we were able to show that treatment of isolated mitochondria with both photoisomers led to a difference of approximately 70% of the velocity of DCIP reduction. We could further show that the closed isomer **1b^c^** can restart the DCIP reduction upon activation through irradiation. Thus, we present the first photochromic redox cofactor that operates in a complex, biological context. The photochromic compound converts upon irradiation from the formally caged closed form of the mimetic into an active redox probe inside the system. However, reversibility of the redox reaction could not yet be achieved due to low PSS values and insufficiently fast reaction rates. Optimization of both issues should be addressed in further research to finally obtain a tool facilitating the control of redox-dependent biological functions in cells by light.

## References

[cit1] Brieke C., Rohrbach F., Gottschalk A., Mayer G., Heckel A. (2012). Angew. Chem., Int. Ed..

[cit2] Szymański W., Beierle J. M., Kistemaker H. A. V., Velema W. A., Feringa B. L. (2013). Chem. Rev..

[cit3] Velema W. A., Szymanski W., Feringa B. L. (2014). J. Am. Chem. Soc..

[cit4] Lerch M. M., Hansen M. J., van Dam G. M., Szymanski W., Feringa B. L. (2016). Angew. Chem., Int. Ed..

[cit5] Wilson D. and Branda N. R., in Photochromic Materials, ed. H. Tian and J. Zhang, Wiley-VCH Verlag GmbH & Co, KGaA, Weinheim, 2016, ch. 9, pp. 361–391.

[cit6] Westmark P. R., Kelly J. P., Smith B. D. (1993). J. Am. Chem. Soc..

[cit7] Falenczyk C., Schiedel M., Karaman B., Rumpf T., Kuzmanovic N., Grotli M., Sippl W., Jung M., Konig B. (2014). Chem. Sci..

[cit8] Vomasta D., Högner C., Branda N. R., König B. (2008). Angew. Chem., Int. Ed..

[cit9] Trads J. B., Burgstaller J., Laprell L., Konrad D. B., de la Osa de la Rosa L., Weaver C. D., Baier H., Trauner D., Barber D. M. (2017). Org. Biomol. Chem..

[cit10] Reisinger B., Kuzmanovic N., Löffler P., Merkl R., König B., Sterner R. (2014). Angew. Chem., Int. Ed..

[cit11] Szymanski W., Ourailidou M. E., Velema W. A., Dekker F. J., Feringa B. L. (2015). Chem.–Eur. J..

[cit12] Lien L., Jaikaran D. C. J., Zhang Z., Woolley G. A. (1996). J. Am. Chem. Soc..

[cit13] Babii O., Afonin S., Berditsch M., Reiβer S., Mykhailiuk P. K., Kubyshkin V. S., Steinbrecher T., Ulrich A. S., Komarov I. V. (2014). Angew. Chem., Int. Ed..

[cit14] Velema W. A., van der Berg J. P., Hansen M. J., Szymanski W., Driessen A. J. M., Feringa B. L. (2013). Nat. Chem..

[cit15] Borowiak M., Nahaboo W., Reynders M., Nekolla K., Jalinot P., Hasserodt J., Rehberg M., Delattre M., Zahler S., Vollmar A., Trauner D., Thorn-Seshold O. (2015). Cell.

[cit16] Irie M. (2000). Chem. Rev..

[cit17] Cahová H., Jäschke A. (2013). Angew. Chem., Int. Ed..

[cit18] Singer M., Jäschke A. (2010). J. Am. Chem. Soc..

[cit19] Morgan C. G., Thomas E. W., Yianni Y. P., Sandhu S. S. (1985). Biochim. Biophys. Acta, Biomembr..

[cit20] Frank J. A., Moroni M., Moshourab R., Sumser M., Lewin G. R., Trauner D. (2015). Nat. Commun..

[cit21] Frank J. A., Yushchenko D. A., Hodson D. J., Lipstein N., Nagpal J., Rutter G. A., Rhee J.-S., Gottschalk A., Brose N., Schultz C., Trauner D. (2016). Nat. Chem. Biol..

[cit22] Morgan C. G., Yianni Y. P., Sandhu S. S., Mitchell A. C. (1995). Photochem. Photobiol..

[cit23] Frank J. A., Franquelim H. G., Schwille P., Trauner D. (2016). J. Am. Chem. Soc..

[cit24] Wilson D., Branda N. R. (2012). Angew. Chem., Int. Ed..

[cit25] Kamei T., Fukaminato T., Tamaoki N. (2012). Chem. Commun..

[cit26] Berg J. M., Tymoczko J. L. and Stryer L., Biochemistry, W.H. Freeman, New York, 2002.

[cit27] Cantó C., Auwerx J. (2011). Cold Spring Harbor Symp. Quant. Biol..

[cit28] Lenaz G., Genova M. L. (2009). Biochim. Biophys. Acta, Bioenerg..

[cit29] Bentinger M., Brismar K., Dallner G. (2007). Mitochondrion.

[cit30] Beyer R. E. (1994). J. Bioenerg. Biomembr..

[cit31] Cornell B. A., Keniry M. A., Post A., Robertson R. N., Weir L. E., Westerman P. W. (1987). Biochemistry.

[cit32] Turunen M., Wehlin L., Sjöberg M., Lundahl J., Dallner G., Brismar K., Sindelar P. J. (2002). Biochem. Biophys. Res. Commun..

[cit33] Göstl R., Hecht S. (2014). Angew. Chem., Int. Ed..

[cit34] Deng X., Liebeskind L. S. (2001). J. Am. Chem. Soc..

[cit35] Katsumura S., Yoshida S., Kubo H. and Saiga T., Jp. Pat., JP09077743A, 1997.

[cit36] Vilà N., Royal G., Loiseau F., Deronzier A. (2011). Inorg. Chem..

[cit37] Goulle V., Harriman A., Lehn J.-M. (1993). J. Chem. Soc., Chem. Commun..

[cit38] Sánchez R. S., Gras-Charles R., Bourdelande J. L., Guirado G., Hernando J. (2012). J. Phys. Chem. C.

[cit39] Goulet-Hanssens A., Utecht M., Mutruc D., Titov E., Schwarz J., Grubert L., Bléger D., Saalfrank P., Hecht S. (2017). J. Am. Chem. Soc..

[cit40] Al-Atar U., Fernandes R., Johnsen B., Baillie D., Branda N. R. (2009). J. Am. Chem. Soc..

[cit41] Herder M., Utecht M., Manicke N., Grubert L., Patzel M., Saalfrank P., Hecht S. (2013). Chem. Sci..

[cit42] Gao X., Wen X., Esser L., Quinn B., Yu L., Yu C.-A., Xia D. (2003). Biochemistry.

[cit43] Ma J., Cui X., Wang F., Wu X., Zhao J., Li X. (2014). J. Org. Chem..

[cit44] Matsumoto M., Kobayashi H. (1985). J. Org. Chem..

[cit45] Yu D., Mattern D. L. (1999). Synth. Commun..

[cit46] Yamaguchi T., Uchida K., Irie M. (1997). J. Am. Chem. Soc..

[cit47] Yamaguchi T., Irie M. (2004). Chem. Lett..

[cit48] Guin P. S., Das S., Mandal P. C. (2011). Int. J. Electrochem..

[cit49] Pavlishchuk V. V., Addison A. W. (2000). Inorg. Chim. Acta.

[cit50] Shim Y.-B., Park S.-M. (1997). J. Electroanal. Chem..

[cit51] Saito E., Ako T., Kobori Y., Tsuda A. (2017). RSC Adv..

[cit52] Chen W. W., Niepel M., Sorger P. K. (2010). Genes Dev..

[cit53] Becke A. D. (1993). J. Chem. Phys..

[cit54] Lee C., Yang W., Parr R. G. (1988). Phys. Rev. B: Condens. Matter Mater. Phys..

[cit55] Stephens P. J., Devlin J. F., Chabalowski C. F., Frisch M. J. (1994). J. Phys. Chem..

[cit56] Frisch M. J., Trucks G. W., Schlegel H. B., Scuseria G. E., Robb M. A., Cheeseman J. R., Scalmani G., Barone V., Petersson G. A., Nakatsuji H., Li X., Caricato M., Marenich A., Bloino J., Janesko B. G., Gomperts R., Mennucci B., Hratchian H. P., Ortiz J. V., Izmaylov A. F., Sonnenberg J. L., Williams-Young D., Ding F., Lipparini F., Egidi F., Goings J., Peng B., Petrone A., Henderson T., Ranasinghe D., Zakrzewski V. G., Gao J., Rega N., Zheng G., Liang W., Hada M., Ehara M., Toyota K., Fukuda R., Hasegawa J., Ishida M., Nakajima T., Honda Y., Kitao O., Nakai H., Vreven T., Throssell K., Montgomery Jr J. A., Peralta J. E., Ogliaro F., Bearpark M., Heyd J. J., Brothers E., Kudin K. N., Staroverov V. N., Keith T., Kobayashi R., Normand J., Raghavachari K., Rendell A., Burant J. C., Iyengar S. S., Tomasi J., Cossi M., Millam J. M., Klene M., Adamo C., Cammi R., Ochterski J. W., Martin R. L., Morokuma K., Farkas O., Foresman J. B. and Fox D. J., Gaussian 09, Revision A.02, Gaussian, Inc., Wallingford CT, 2016.

[cit57] Kuhn B., Kollman P. A. (2000). J. Am. Chem. Soc..

[cit58] Gregg C., Kyryakov P. and Titorenko V. I., J. Visualized Exp., 2009, 30, e1417. 10.3791/1417 PMC3149909 19704406

[cit59] Reddy C. A., Beveridge T. J., Breznak J. A., Marzluf G. A., Schmidt T. M. and Snyder L. R., Methods for General and Molecular Microbiology, American Society of Microbiology, 2007.

