# Supplementary material for: Photochromic coenzyme Q derivatives: switching redox potentials with light
Source: Chem Sci. 2017 Jul 20;8(9):6474–83. doi: 10.1039/c7sc00781g (PMC5628583; doi:10.1039/c7sc00781g)
Supplement: Supplementary file 1 [file SC-008-C7SC00781G-s001.pdf]

Electronic Supplementary Material (ESI) for Chemical Science.
This journal is © The Royal Society of Chemistry 2017

Photochromic Coenzyme Q Derivatives: Switching Redox Potentials with Light

Nadja A. Simeth,[a] Andrea C. Kneuttinger,[b] Reinhard Sterner[b,*] and Burkhard König[a,*]

[a] University of Regensburg, Faculty of Chemistry and Pharmacy, Institute of Organic Chemistry, Universitätsstraße 31, 93053 Regensburg, Germany.

[b] University of Regensburg, Faculty of Biology and Preclinical Medicine, Institute of Biophysics and Physical Biochemistry, Universitätsstraße 31, 93053 Regensburg, Germany.

Supporting Information

Contents

1. General preliminary remarks 3

2. Synthesis 5

2.1. Compound 1a: 2,3-Bis(2,5-dimethylthiophen-3-yl)cyclohexa-2,5-diene-1,4-dione 5

2.2. Compound 1b^{o}: 2,3-Dimethoxy-5,6-bis(2-methyl-5-phenylthiophen-3-yl)cyclohexa-2,5-diene-1,4-dione 6

2.3. Compound 1b^{c}: (10a,10b)-5,6-Dimethoxy-10a,10b-dimethyl-2,9-diphenyl-10a,10b-dihydronaphtho[2,1-b:3,4-b']dithiophene-4,7-dione 7

2.4- Compound 5a: 3-Bromo-2,5-dimethylthiophene 7

2.5. Compound 5b: 3-Bromo-2-methyl-5-phenylthiophene 8

2.6. Compound 9b: 2,3-Dibromo-5,6-dimethoxycyclohexa-2,5-diene-1,4-dione 8

3. UV/Vis Spectroscopy: Further spectra 10

4. Cyclic Voltammery and Spectroelectrochemical Studies (further material) 14

4.1. Compound 1b^{o} 14

4.2. Compound 1b^{c} 18

4.3. Compound 8b 23

4.4. Compound 9b 24

4.5. Compound 10 (CoQ_{10}) 27

4.6. Compound 11 29

4.7. Compound 12 31

4.8. DCIP 32

4.9. PMS 33

5. Photoactivated Oxidation 34

6. Activation of 1b^{c} on isolated Mitochondria 45

7. Computational Studies 47

8. NMR spectra 48

- Compound 1a^{o} 48
- Compound 1b^{o} 49
- Compound 1b^{c} 50
- Compound 9b 50

9. X-Ray Structure of 9b 52

10. Cartesian Coordinates 58

11. References 86

1. General preliminary remarks

a) General working methods

All reactions were performed using magnetic stirring under ambient atmosphere unless otherwise specified. Reactions requiring anhydrous conditions were carried out under dry nitrogen or argon atmosphere using standard Schlenk techniques. Oxygen sensitive reactions were degassed under vacuum and performed under nitrogen or argon atmosphere or deaerated through argon bubbling.

All reagents and solvents were obtained from commercial suppliers and used without further purification if not otherwise stated.

For TLC, silica coated aluminum plates (Merck $60F^{254}$ silica gel, 0.2 mm) were utilized. Visualization was done with UV light at 254 or 366 nm or through suitable staining.

b) Isolation of compounds

For MPLC a Biotage Isolera One Flash Purification System with manually packed columns using Merck Geduran SI 60 (70-230 grain diameter) or Macherey- Nagel GmbH & Co. KG 60M (0.04-0.063 mm, 230-400 grain diameter) was utilized.

c) Analysis

Nuclear magnetic resonance spectroscopy (NMR) was carried out using a Bruker Avance 300 MHz spectrometer (^{1}H: 300 MHz, ^{13}C: 75 MHz, T = 300 K), a Bruker Avance 400 MHz spectrometer (^{1}H: 400 MHz, ^{13}C: 101 MHz, T = 300K) or a Bruker Avance 600 MHz spectrometer (^{1}H: 600 MHz, ^{13}C: 151 MHz, T = 300 K). Chemical shifts are reported in δ [ppm] relative to an internal standard (solvent residual peak). The used solvents are indicated for each spectrum. Coupling constants are reported in Hertz [Hz]. Characterization of the signals: s = singlet, d = doublet, t = triplet, q = quartet, m = multiplet, bs = broad singlet, dd = doublet of doublet, dt = doublet of triplet. Integration is directly proportional to the number of the protons. Characterization of the ^{13}C-NMR signals: (+) for CH3 or CH, (-) for CH2 and (q) for quaternary C-atoms. The assignment resulted from DEPT135, ATP, HMBC, HSQC and NOESY experiments.
CV measurements and spectroelectrochemical studies were carried out under argon atmosphere. All measurements were performed in MeCN containing 0.1 M tertra ^{n}butylammonium tertra fluoroborate using ferrocene/ferrocenium (Fc/Fc+) as an internal reference. A glassy carbon electrode (working electrode), platinum wire counter electrode, and Ag quasi-reference electrode were employed for CV measurements. The scan rate was 50 mV/s, a step potential of 5.0 mV was applied. Spectroelectrochemical studies were carried out in an optically transparent thin layer electrochemical cell (OTTLE). The scan rate was 2 mV/s, a step potential of 0.02 V was applied.

IR spectroscopy was done using an Agilent Cary 630 FTIR. Melting points were recorded on a Stanford Research Systems OptiMelt (MPA100).

Steady state absorption measurements were carried out on a Varian Cary Bio 50 UV/Vis spectrometer. The used solvent is stated for each experiment. UV-induced isomerizations were performed at ambient temperature using a Herolab hand-lamp (254 nm, 2x8W or 312 nm, 1x6W) or an OSRAM Oslon SSL 80 single-spot LED (590 nm, 0.7 W). Sample volume was 2.5 mL. Photo stationary distribution as well as monitoring of photoactivated oxidation experiments was analyzed through separation on an 1220 Infinity LC System from Agilent with a reverse phase Phenomenex Luna® 3μ C18(2) column (150 × 2.0 mm, 100 Å) thermostatted at 40 °C. HPLC conditions were the following: solvent A = water (Millipore)/TFA (0.05% v/v), solvent B = MeCN (Fisher scientific, gradient grade); flow rate = 0.3 mL/min; injection volume 0.5 μL, elution with a gradient of 30% to 98% MeCN in 25 min for purity (220 nm, VWD detector) and 40% to 98% MeCN in 20 min for photoactivated oxidation monitoring and PSS (isosbestic point @317 nm, VWD detector).

2. Synthesis

2.1. Compound **1a**: 2,3-Bis(2,5-dimethylthiophen-3-yl)cyclohexa-2,5-diene-1,4-dione

Chemical Formula: $C_{18}H_{16}O_2S_2$

Molecular Weight: 328.44

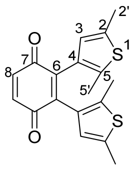

A crimp top vial was equipped with **8a** (97 mg, 0.36 mmol), **6a** (591 mg, 1.47 mmol), CuI (30 mg, 0.16 mmol) and dry toluene (7 mL) under nitrogen. The vial was deaerated through argon bubbling for five minutes. Then $Pd(PPh_3)_4$ (173 mg, 0.15mmol) was added and the vial was sealed and heated to 85 °C for 16h. The reaction mixture was cooled to room temperature and the solvent was removed *in vacuo*. The residue was dissolved in EtOAc (5 mL), a saturated KF solution (aq, 5 mL) was added and the mixture was stirred for one hour. The reaction mixture was filtered to remove precipitated Bu_3SnF and the organic layer was isolated. The aqueous layer was extracted with EtOAc (2 x 10 mL). The combined organic layers were dried over $MgSO_4$ and the solvent was removed *in vacuo*. The crude product was purified through MPLC (PE : EtOAc = 0 to 20%). The pure product was isolated as brown solid.

Yield: 12 mg, 0.04 mmol, 10%.

^{1}H-NMR: δ_H(400 MHz, CDCl3): 6.76 (s, 2H, H8), 6.63 (d, *J* = 1.3 Hz, 2H, H3), 2.46 (s, 6H, H2’), 2.34 (s, 6H, H5’).

^{13}C-NMR: δ_C(101 MHz, CDCl3): 146.4 (s, C7) , 137.6 (s, C2), 134.8 (s, C5), 132.3 (s, C4) , 126.9 (s, C3), 123.4 (s, C6), 116.4 (s, C8), 15.2 (s, C2’) , 13.8 (s, C5’).

ESI-MS: found: 331.08 (reduced form; MH^+, 100%).

HR-MS: found: 329.0665 (MH^+, 100%).

R_f: 0.85 (30% PE in EA).

2.2. Compound **1b**[a]: 2,3-Dimethoxy-5,6-bis(2-methyl-5-phenylthiophen-3-yl)cyclohexa-2,5-diene-1,4-dione

Chemical Formula: $C_{30}H_{24}O_4S_2$

Molecular Weight: 512.11

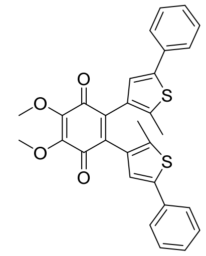

Compounds **5b** (1.42 g, 3.97 mmol), **8a** (522 mg, 1.59 mmol) and CuI (30 mg, 0.16 mmol) were dissolved in dry toluene (25 mL) under Argon atmosphere. The solution was deaerated through Argon bubbling for 5 min and $Pd(PPh)_3$ (183 mg, 0.16 mmol) was added. The vial was sealed and the reaction mixture was refluxed for 16h. The reaction mixture was cooled to room temperature and the solvent was removed *in vacuo*. The residue was dissolved in EtOAc (10 mL), a saturated KF solution (aq, 10 mL) was added and the mixture stirred for one hour. The reaction mixture was filtered to remove precipitated Bu_3SnF and the organic layer was isolated. The aqueous layer was extracted with EtOAc (2 x 20 mL). The organic layers were combined and dried over Na_2SO_4. The volatiles were removed *in vacuo* and the crude product was purified through MPLC (EA (0-55%) in PE) to give a red solid. For biological testing the sample was further purified through reversed phase MPLC (C18, 0.05% TFA in H_2O/MeCN, gradient 65 to 100%).

Yield: 560 mg, 1.04 mmol, 29%.

^{1}H-NMR: δ_H(600 MHz, CDCl3): 7.44 – 7.40 (m, 4H, phenyl), 7.34 – 7.28 (m, 4H), 7.24 (d, *J* = 7.3 Hz, 4H, phenyl), 6.83 (s, 2H, thiophene), 4.11 (s, 6H, methoxy), 2.07 (s, 6H, methyl).

^{13}C-NMR: δ_C(151 MHz, CDCl3): 183.2 (+), 144.6 (+), 140.3 (+), 139.6 (+), 138.00 (+), 134.0 (+), 130.1 (+), 128.8 (+), 127.3 (+), 125.7 (+), 125.3 , (+) 61.4 (+, methoxy), 14.9 (+, methyl).

FTIR: $\tilde{\nu}$(cm^{-1}): 2937, 1655, 1592, 1256, 1070, 757, 693.

HR-MS: found: 513.1187 (100%).

Melting point: 137 °C.

R_f: 0.65 (PE: EA = 1:1).

2.3. Compound **1b**[c]: (10a,10b)-5,6-Dimethoxy-10a,10b-dimethyl-2,9-diphenyl-10a,10b-dihydronaphtho[2,1-b:3,4-b']dithiophene-4,7-dione

Chemical Formula: $C_{30}H_{24}O_4S_2$

Molecular Weight: 512.11

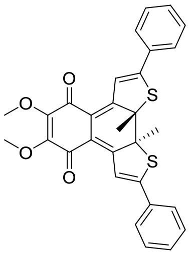

Compound **9b**[o] (29 mg, 0.06 mmol) was dissolved in DMSO (5 mL) and effused into a Petri dish. The solution was irradiated for 4h with a 312 nm hand-lamp and then directly injected onto a reversed phase column. The compound could be isolated through elution with 0.05% TFA in H_2O/MeCN (reversed phase MPLC, C18, gradient 65 to 100%) to give a blue solid.

Yield: 9 mg, 0.01 mmol, 13%.

^{1}H-NMR: δ_H(400 MHz, CDCl3): 8.24 (s, 2H, thiophene), 7.83 – 7.61 (m, 4H, phenyl), 7.45 (dd, *J* = 5.2, 1.9 Hz, 6H, phenyl), 4.08 (s, 6H, methoxy), 2.09 (s, 3H, methyl).

HR-MS: found: 513.1185 (100%).

2.4- Compound **5a**: 3-Bromo-2,5-dimethylthiophene

Molecular formula: C_6H_7BrS

Molecular weight: 191.09 g/mol

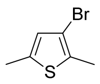

Compound **2** (2.0 mL, 17.54 mmol, 1.0 eq) was dissolved in glacial acetic acid (50 mL). NBS (3120 mg, 17.54 mmol, 1.0 eq) was added in portions. The reaction mixture was stirred for 2h at ambient temperature. The solution was poured onto ice water (50 mL) and extracted with DCM (3 x 8 mL). The combined organic phases were dried over $MgSO_4$. The solvent was evaporated *in vacuo*. The crude product was purified through MPLC (PE). The pure product was obtained as translucent oil.

Yield: 2213 mg (11.58 mmol), 66%.

^{1}H-NMR: δ_H (300 MHz, $CDCl_3$): 2.35 (s, 3H, CH3 thiophene), 2.42 (s, 3H, CH3 thiophene), 6.57 (s, 1H, CH thiophene).

R_f: 0.92 (PE).

The analytical data were in agreement with published data.[1]

2.5. Compound **5b**: 3-Bromo-2-methyl-5-phenylthiophene

Chemical Formula: $C_{11}H_9BrS$

Molecular Weight: 253.16

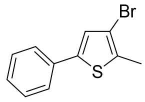

A crimp top vial was equipped with **4** (3390 mg, 13.25 mmol), phenyl boronic acid (1651 mg, 13.52 mmol), $Pd(PPh_3)_2Cl_2$ (88 mg, 0.13 mmol), $AsPh_3$ (69 mg, 0.23 mmol), THF (39 mL) and a Na_2CO_3 solution (aq, 2M, 16 mL) and sealed. The vial was purged with argon for five minutes and the reaction mixture was heated to 80 °C for 16h. The mixture was cooled to room temperature and extracted with EtOAc (2 x 40 mL). The organic layers were combined and dried over $MgSO_4$. The solvent was removed *in vacuo* and the crude product was purified through MPLC (PE). The pure product was isolated as white crystalline powder.

Yield: 2700 mg, 10.67 mmol, 80%

The analytical data were in agreement with published data.[2]

2.6. Compound **9b**: 2,3-Dibromo-5,6-dimethoxycyclohexa-2,5-diene-1,4-dione

Chemical Formula: $C_8H_6Br_2O_4$

Molecular Weight: 325.94

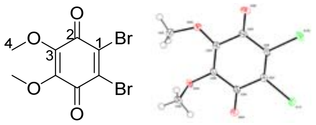

Compound **8b** (768 mg, 4.57 mmol) was dissolved in dry Et_2O (21 mL) under nitrogen atmosphere. Bromine (2773 mg, 17.35 mmol, 0.90 mL) was dissolved in a mixture of $CHCl_3$ (10 mL) and dry Et_2O (2.3 mL) and added dropwise to the reaction mixture at 0 °C. The reaction mixture was stirred for one hour at ambient temperature; then, concentrated sulfuric acid (23 mL) was added dropwise at 0 °C. The reaction mixture was stirred at ambient temperature for another hour and then poured onto ice. The mixture was extracted with Et_2O (3 x 50 mL). Ag_2O (5503 mg, 23.75 mmol) was added to the combined organic layers and stirred for 20 min. The solids were filtered off and the solvent was removed *in vacuo*. The crude product was purified through reversed phase MPLC (C18; H_2O/0.05% TFA : MeCN = 30 to 65 %). The pure product was obtained as red powder and crystallized for analytical purpose from $CHCl_3$/PE.

Yield:	826 mg, 2.54 mmol, 55%.
^{1}H-NMR:	δ_H(600 MHz, CDCl3): 4.04 (s, 6H, H4).
^{13}C-NMR:	δ_C(101 MHz, CDCl3): 174.7 (s, C2), 145.0 (C3) , 137.2 (C1), 61.7 (s, C4).
FTIR:	$\tilde{\nu}$(cm^{-1}): 2948, 1625, 1662, 1562, 936, 731.

APCI-MS: found: 324.87 (MH^+, ^{79}Br, 50%), 326.87 (MH^+, $^{79}Br/^{81}Br$, 100%), 328.87 (MH^+, ^{81}Br, 50%)

HR-MS: 323.8625 ($M^{+\bullet}$,^{79}Br).

Melting point: 102 °C.

R_f: 0.74 (PE : EA = 1:1).

3. UV/Vis Spectroscopy: Further spectra

Compound **1a**^o^

- Compound **1a**^{o} in toluene

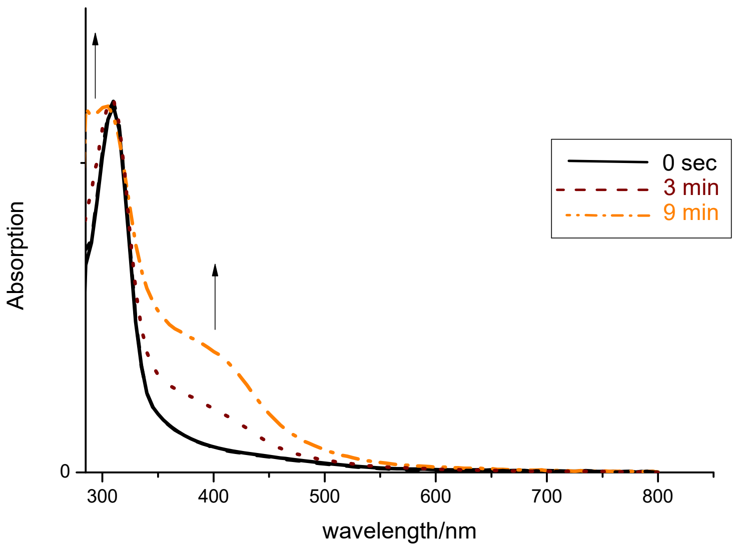

- Compound **1a**^{o} in DCM

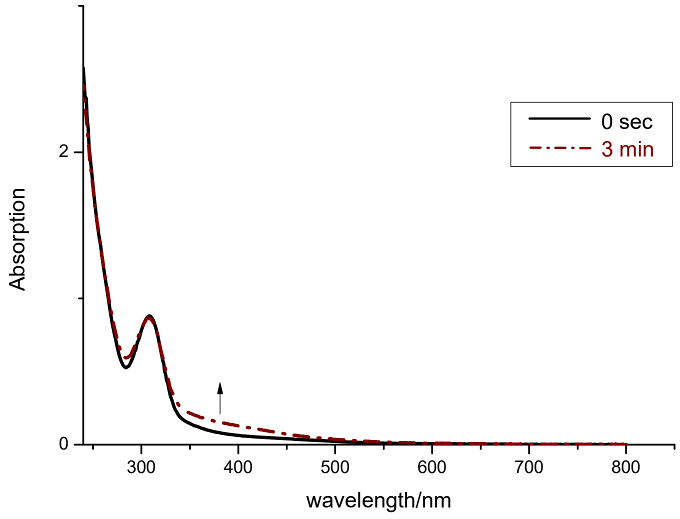

Compound **1b^o**

Table 1: Overview of spectroscopic properties of compound **1b^o** determined by UV/Vis spectroscopy and analytical HPLC.

Solvent	λ_{max} 1b^o [nm]	λ_{max} 1b^c [nm]	PSS [%]
MeCN	247, 297	307, 415, 582	20
DMSO	302	304, 410, 594	52
Toluene	298	296, 413, 580	47
Dioxane	301	294, 399, 595	42

- Compound **1b^o** in DMSO, fatigue resistance

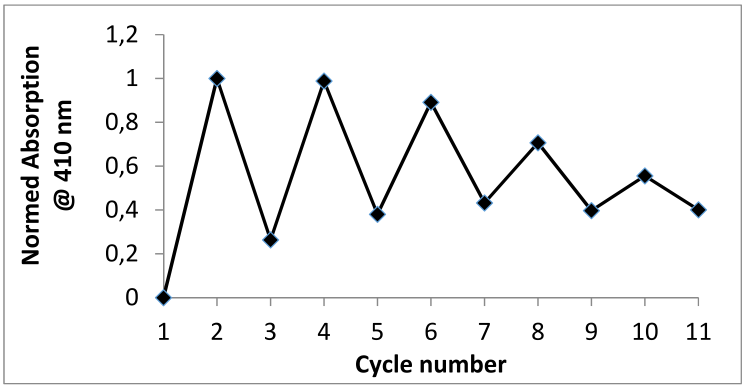

- Compound **1b^o** in toluene

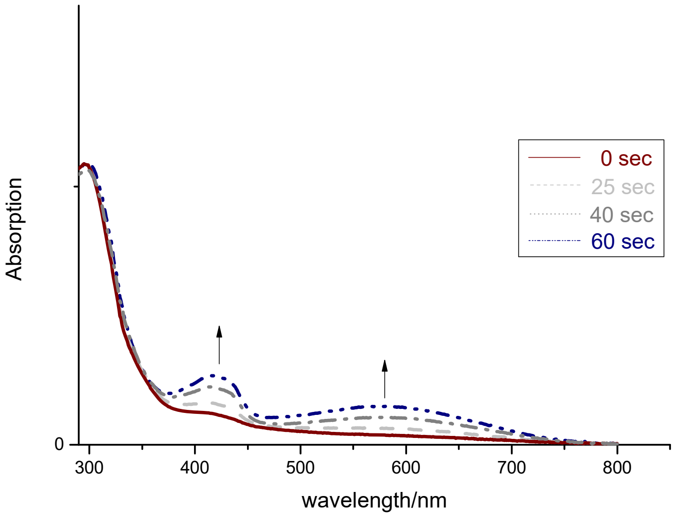

- Compound **1b**^{o} in MeCN

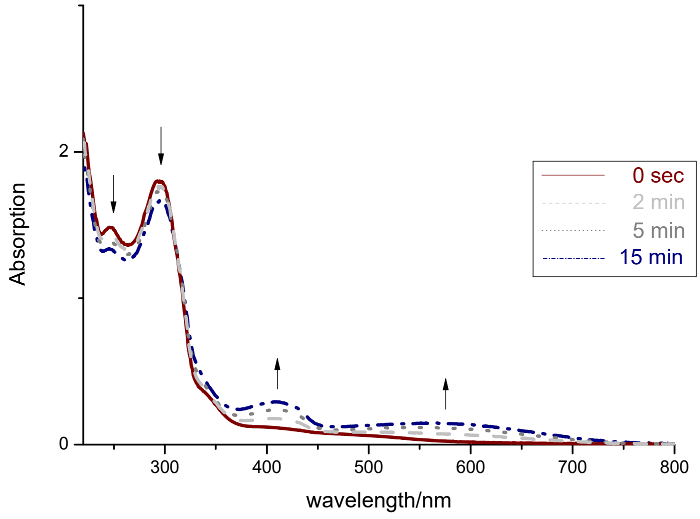

- Compound **1b**^{o} in toluene

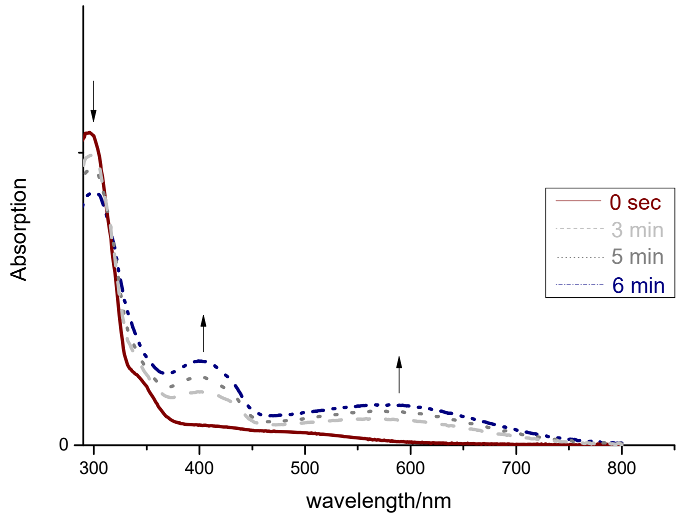

- Compound **1b**^{o} in toluene, fatigue resistance

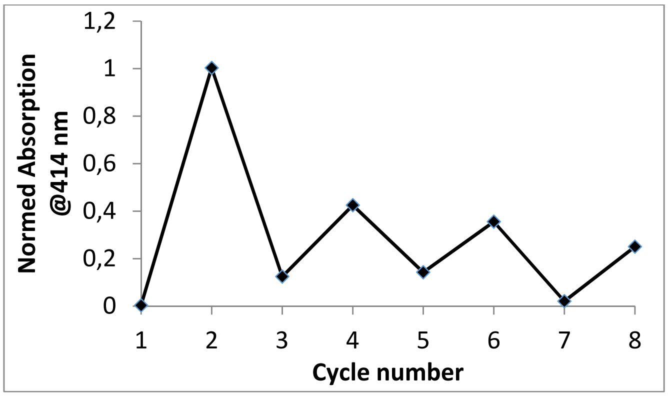

- Extinction coefficients

Compound	Solvent	Extinction coefficient [L/(mol*cm)]
**1b**^{o}	DMSO	ε_{300} = 582
**1b**^{o}	MeCN	ε_{296} =2539
**1b**^{c}	MeCN	ε_{579} =4928

4. Cyclic Voltammery and Spectroelectrochemical Studies (further material)

Molecules included in the study:

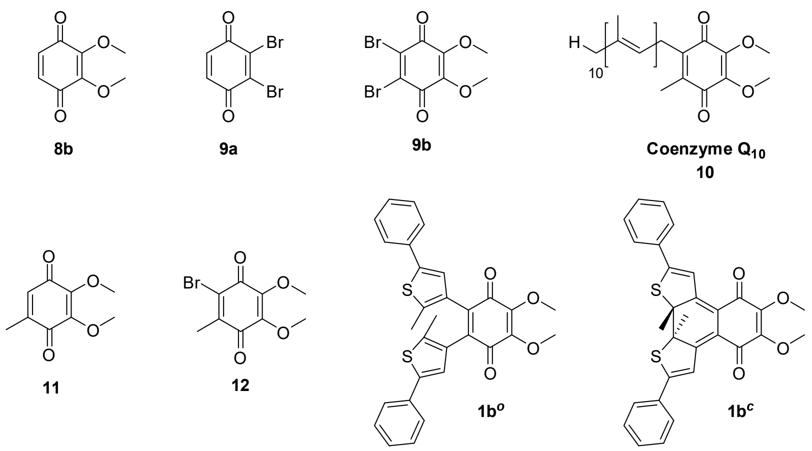

4.1. Compound $\mathbf{1b}^o$

- Cyclic Voltammetry

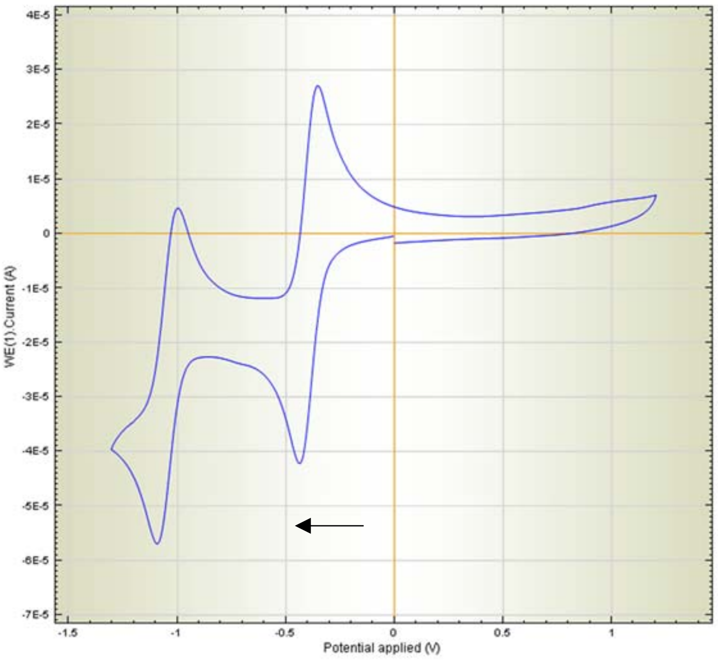

- Spectroelectrochemical studies

Peak 1

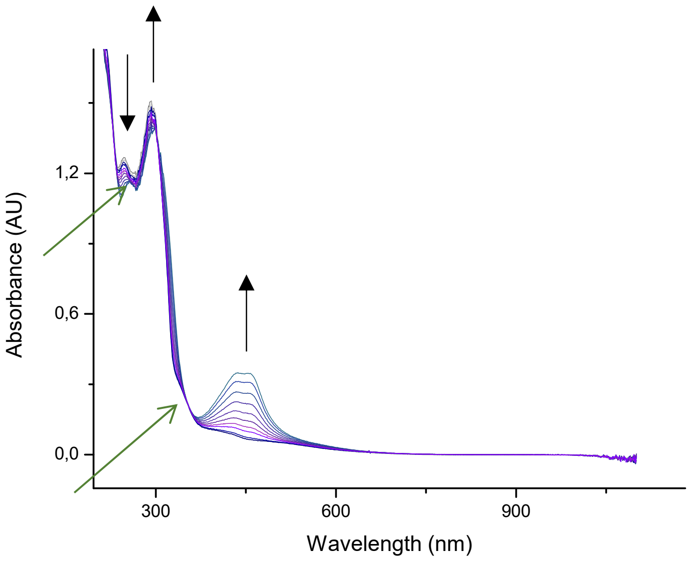

Peak 2

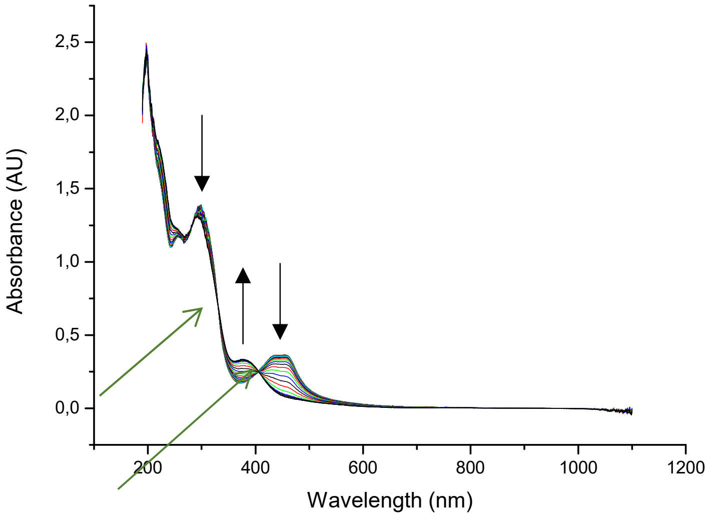

Peak 2 (invers)

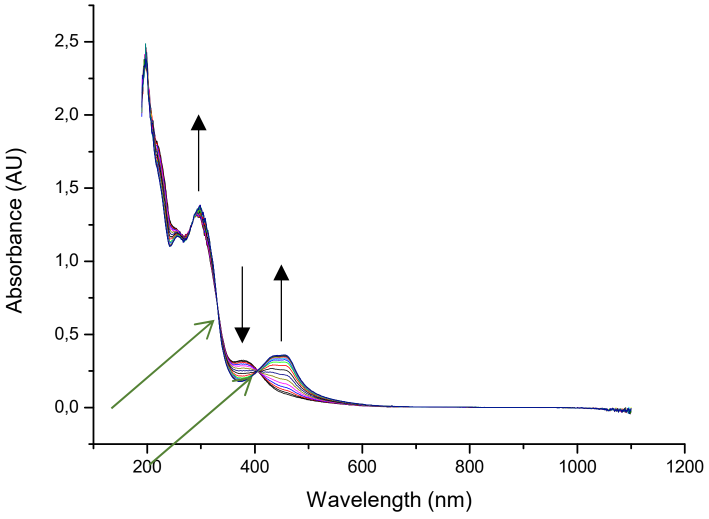

Peak 1 (invers)

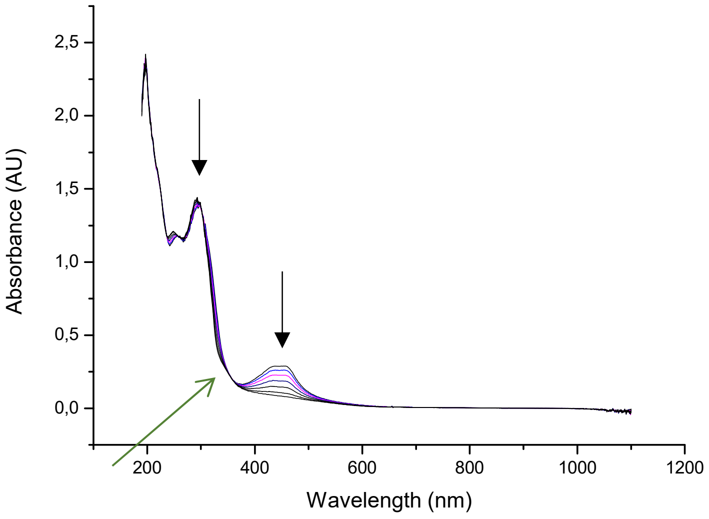

- Difference Spectra

Peak 1

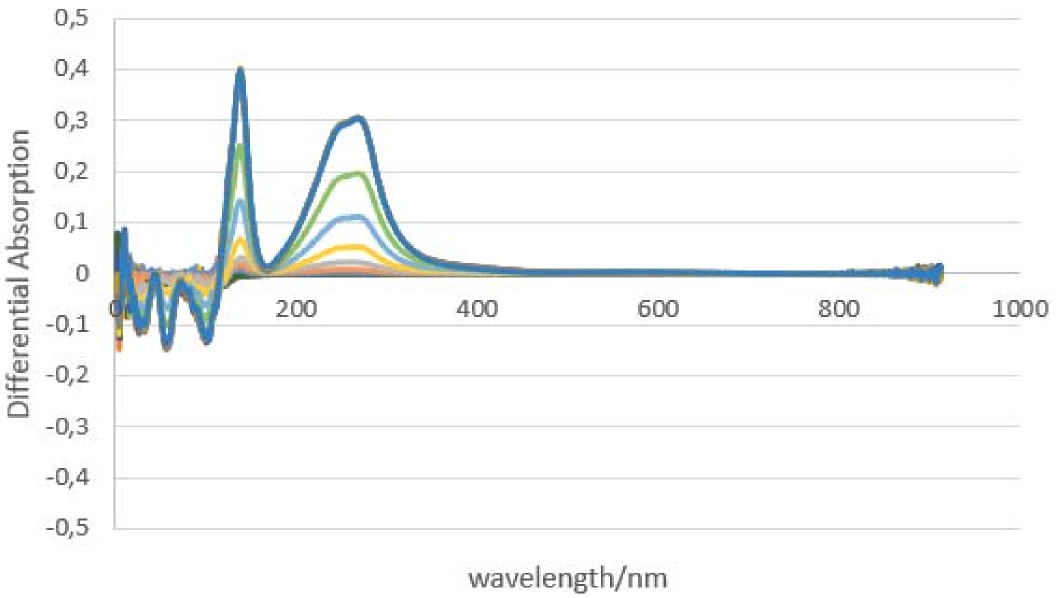

Peak 2

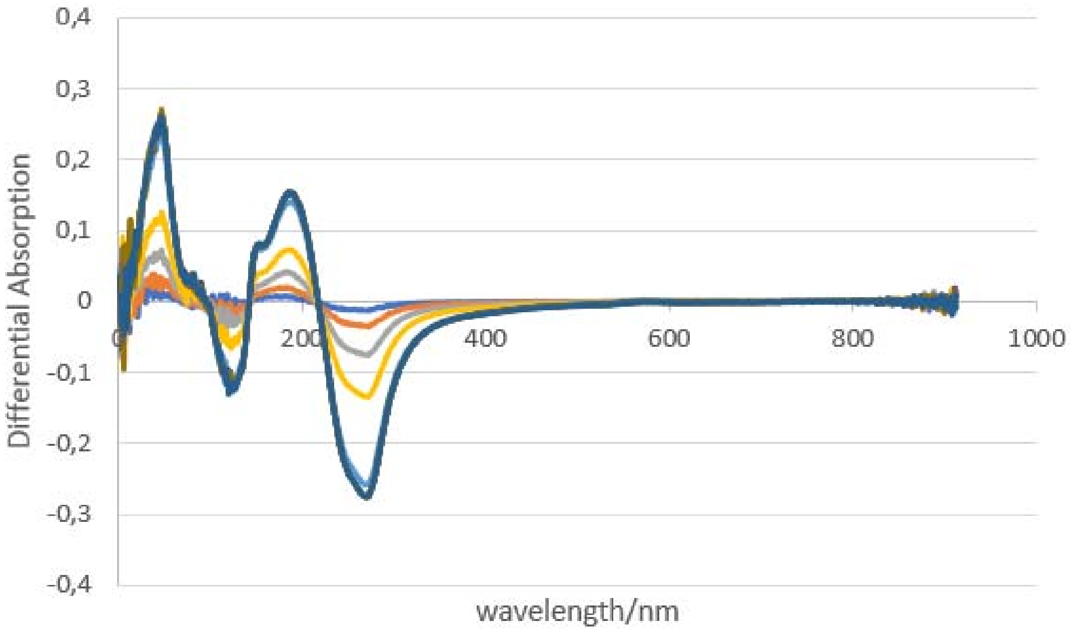

4.2. Compound **1b**^{c}

- Cyclic Voltammetry

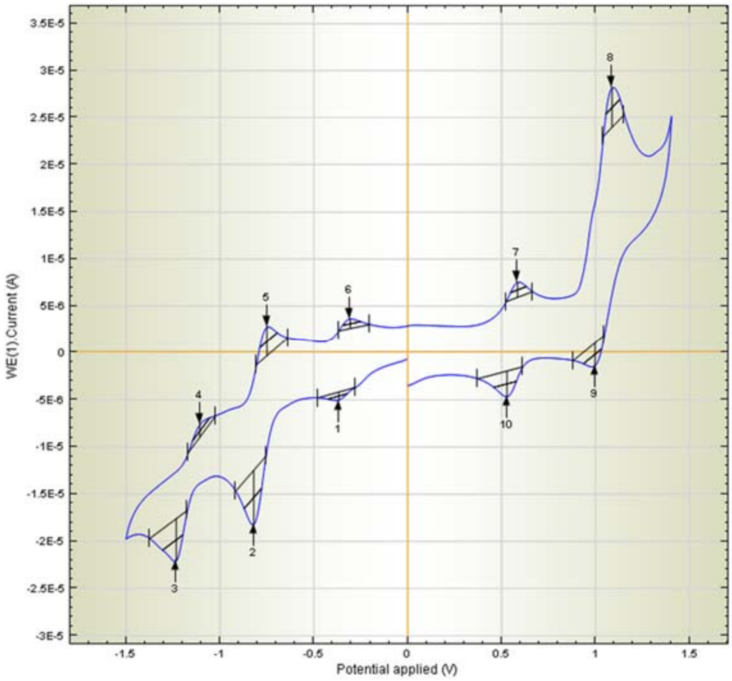

Compound **1b**^{c} with Ferrocene (peaks 7 and 10) as internal standard.

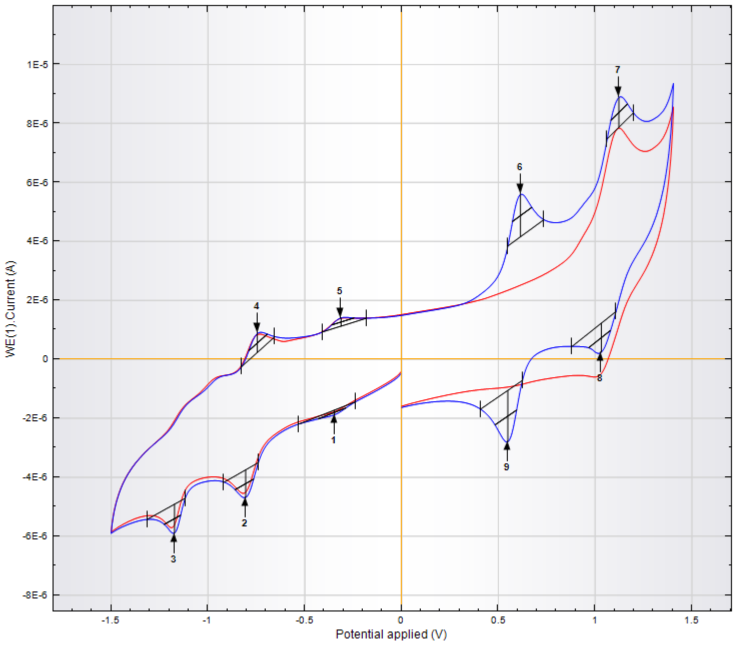

Two consecutive measurements of compound **1b**^{c}: Red: First round, without internal standard; Blue: Second round, after addition of ferrocene (peaks 6 and 9) as internal standard.

- Spectroelectrochemical studies

Peak 1 (0- -0.5 V)

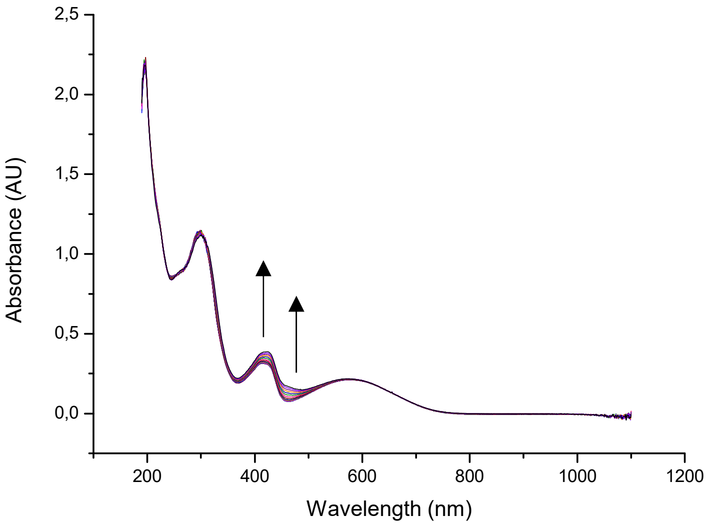

Peak 2 (-0.5 - -1.0V)

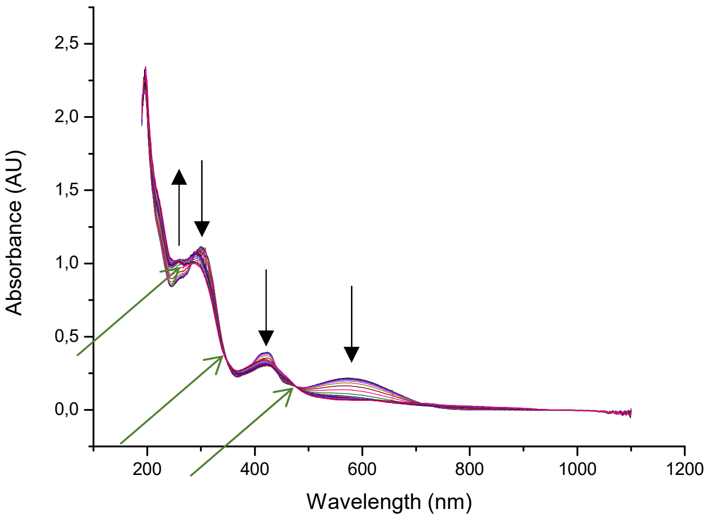

Peak 3 (-1.0 - -1.5V)

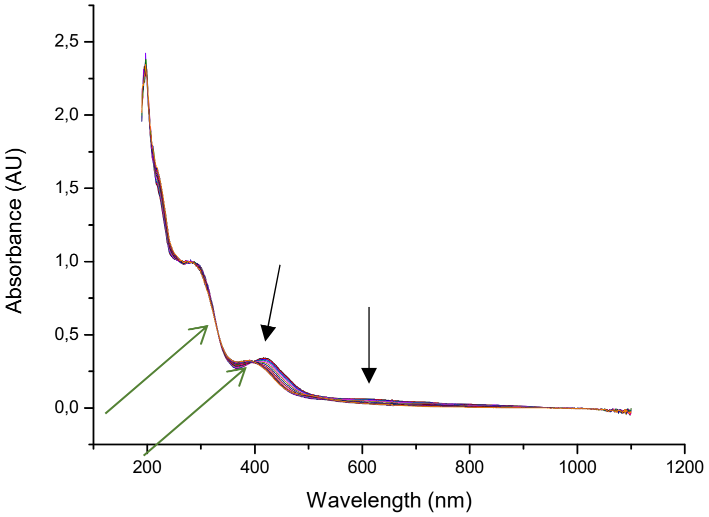

Peak 3 (invers, -1.5V - -1.0V)

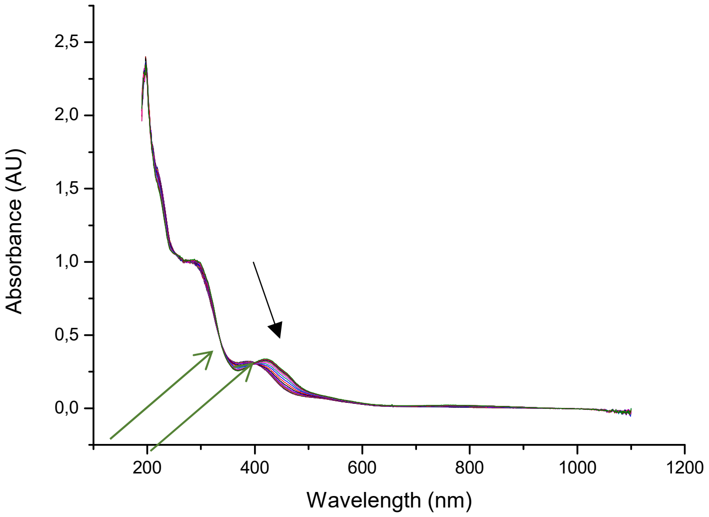

Peak 2 (invers, -1.0 - -0.5V)

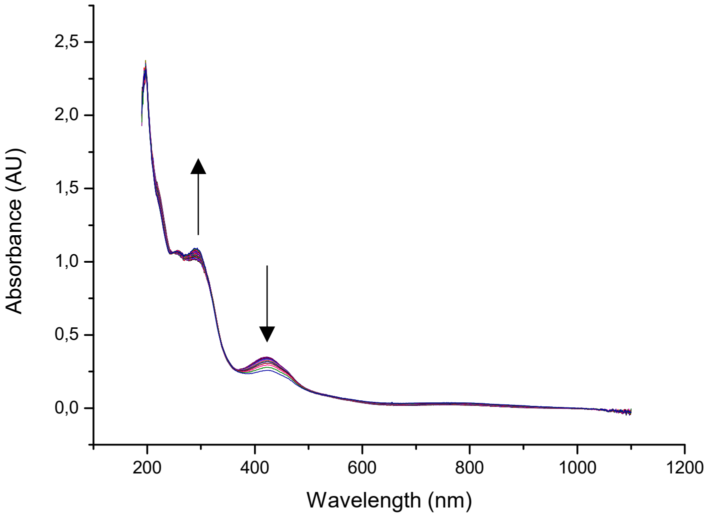

Peak 1 (invers, -0.5 – 0V)

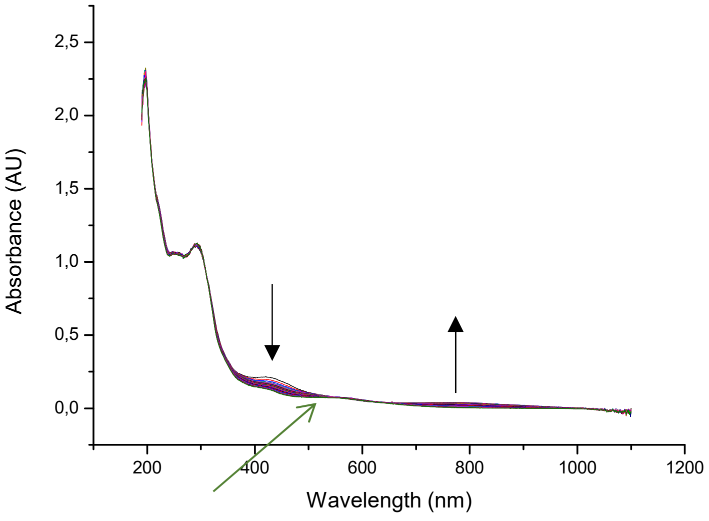

- Difference Spectra

Peak 1

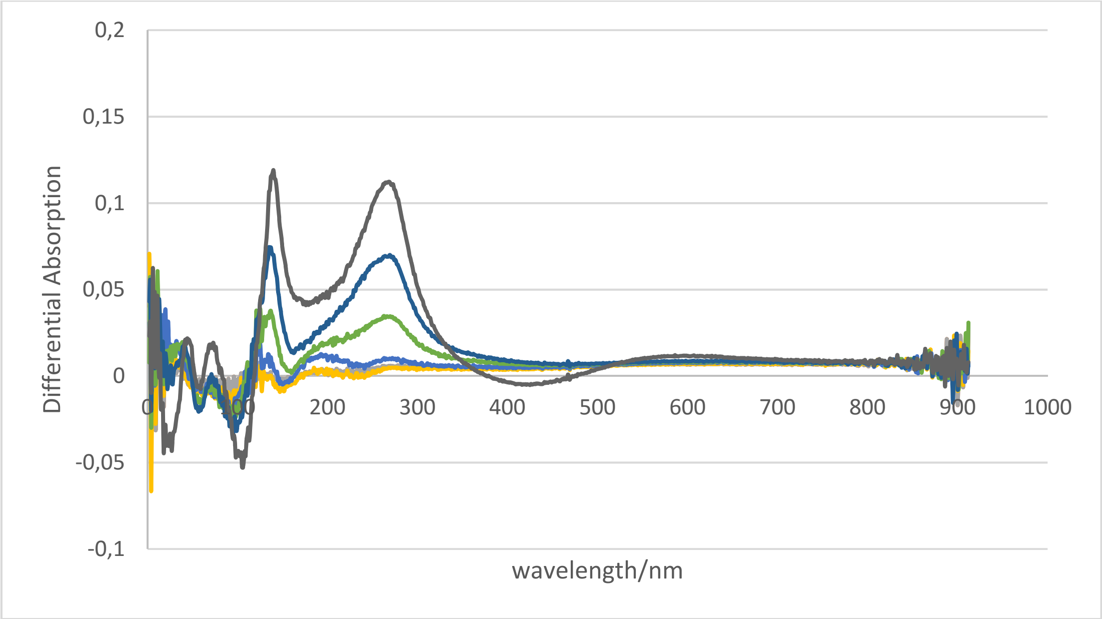

Peak 2

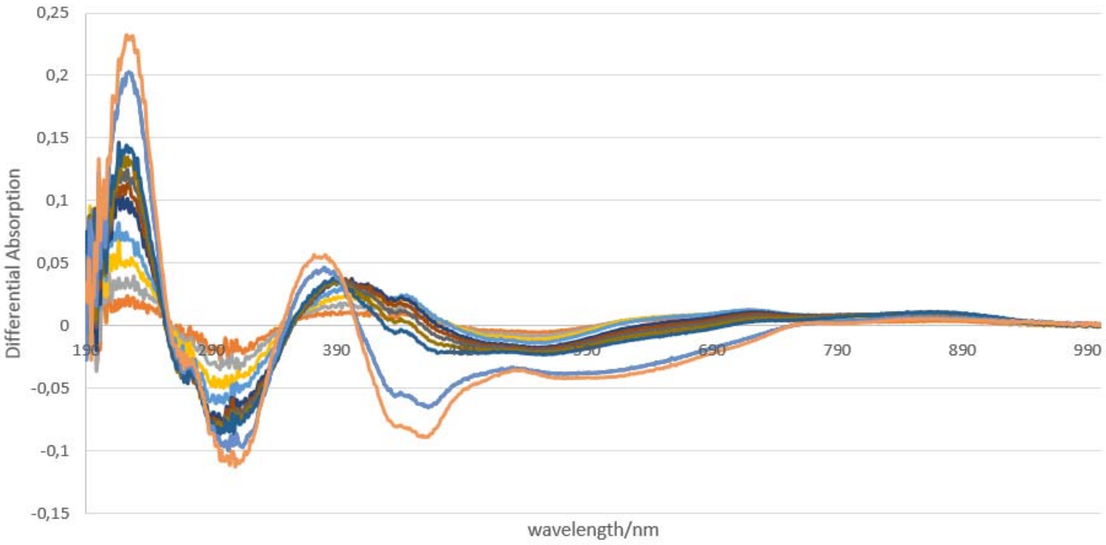

Peak 3

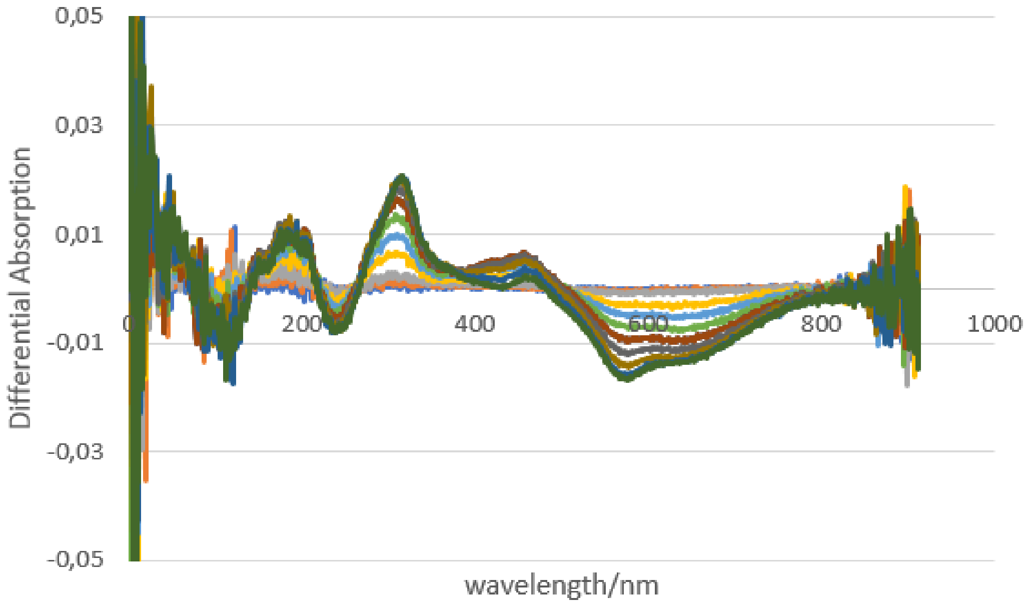

4.3. Compound **8b**

- Cyclic Voltammetry

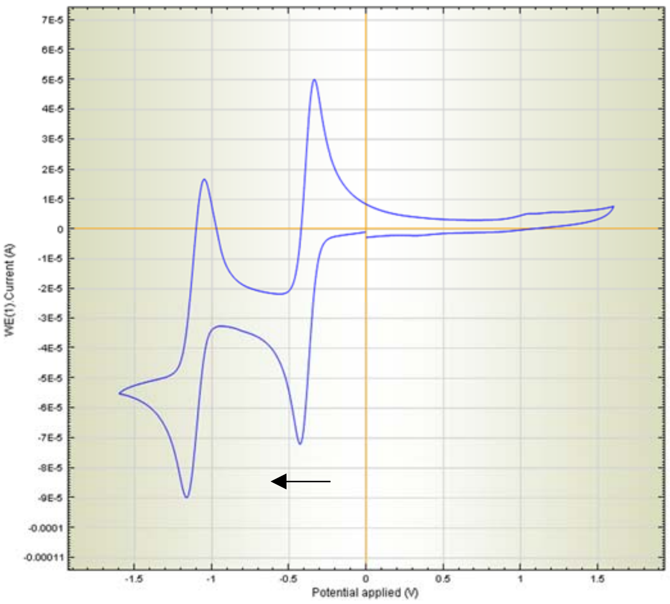

4.4. Compound **9b**

- Cyclic Voltammetry

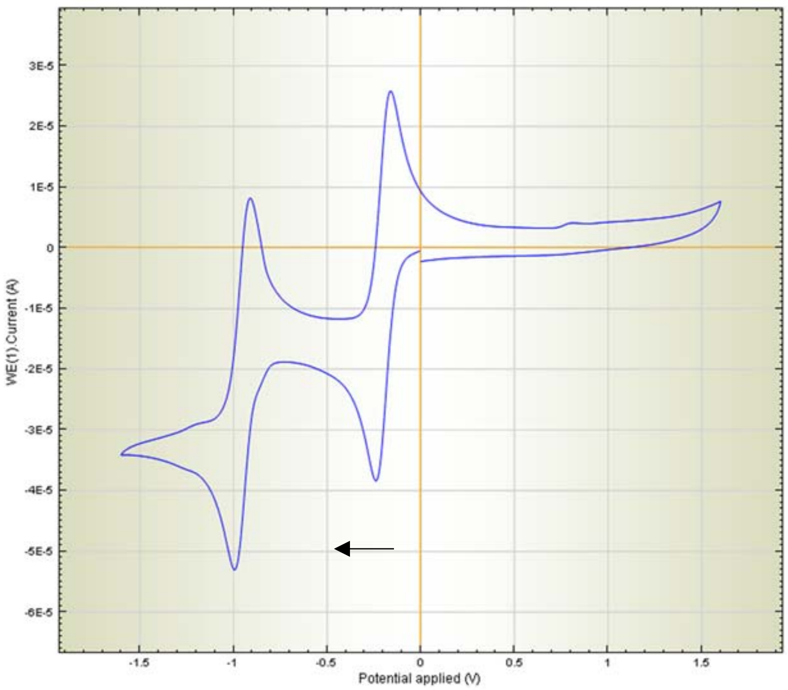

- Spectroelectrochemical studies

Peak 1 (0- -0.5V)

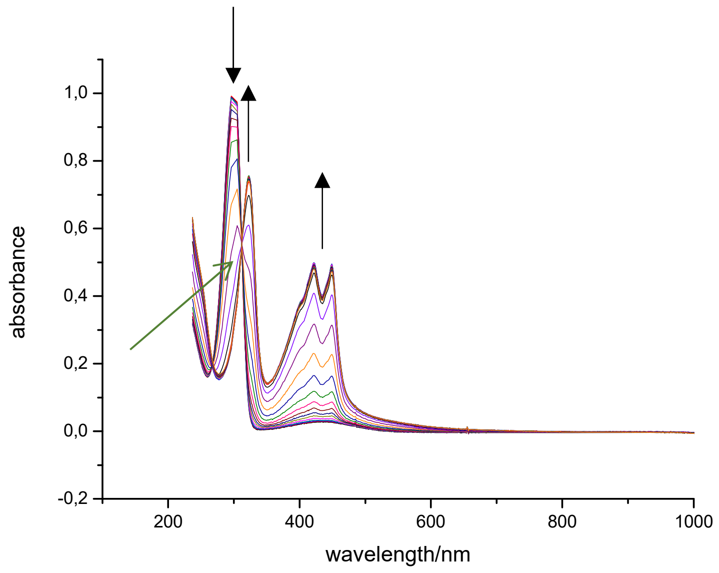

Peak 2 (-0.5 - -1.5V)

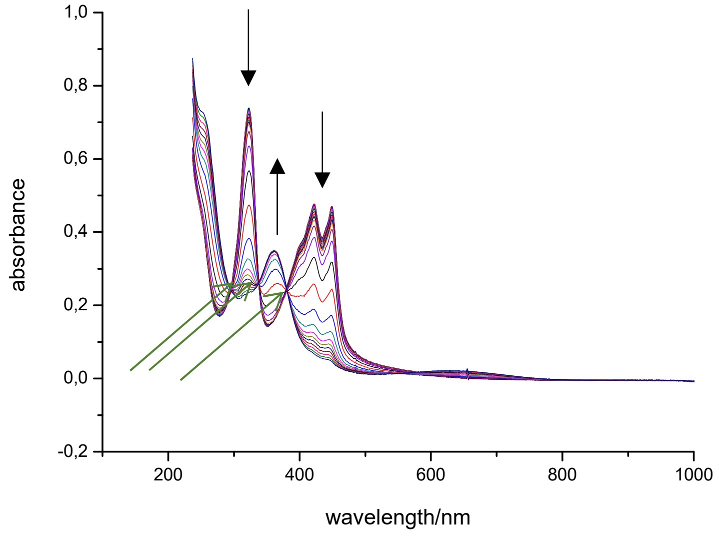

Peak 2 (invers, -1.5V - -0.5V)

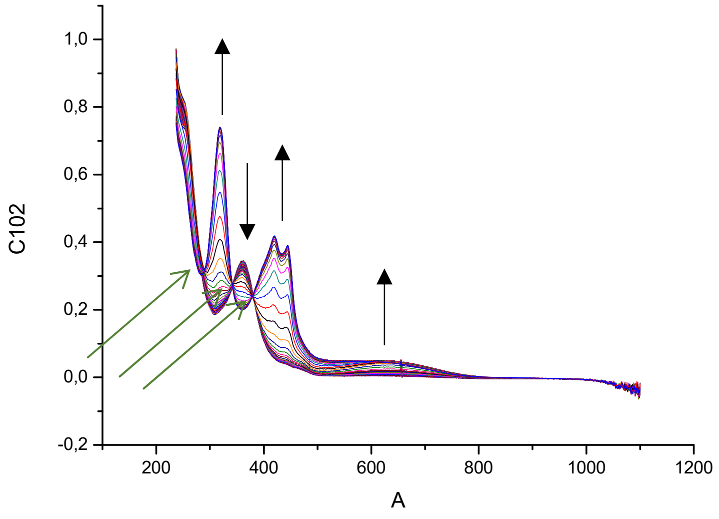

Peak 1 (invers, -0.5 – 0V, incomplete re-oxidation)

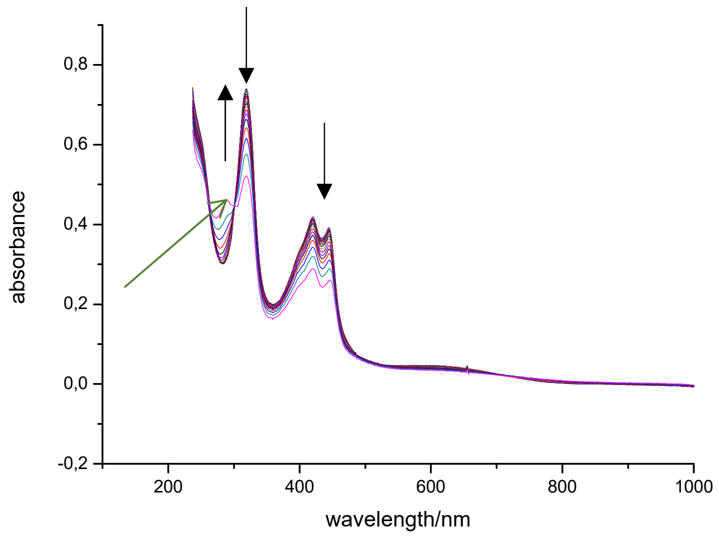

- <u>Difference Spectra</u>

<u>Peak 1</u>

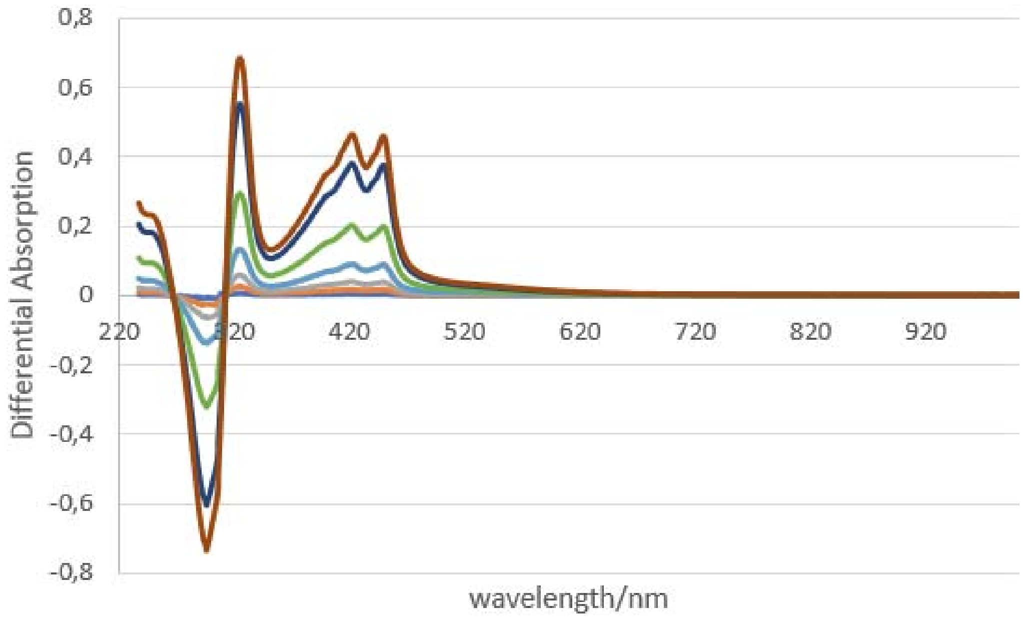

Peak 2

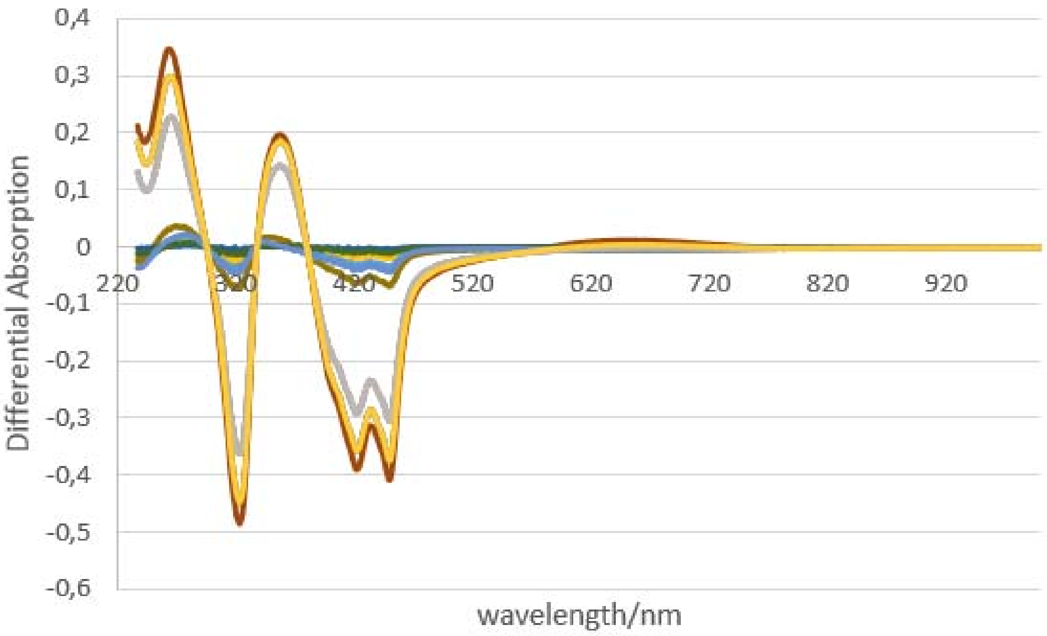

4.5. Compound **10** (CoQ_{10})

- Spectroelectric measurement

Peak 1

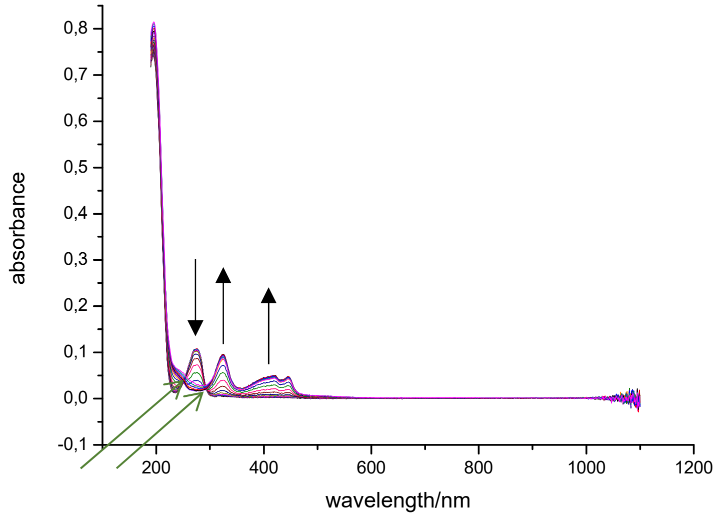

Peak 2

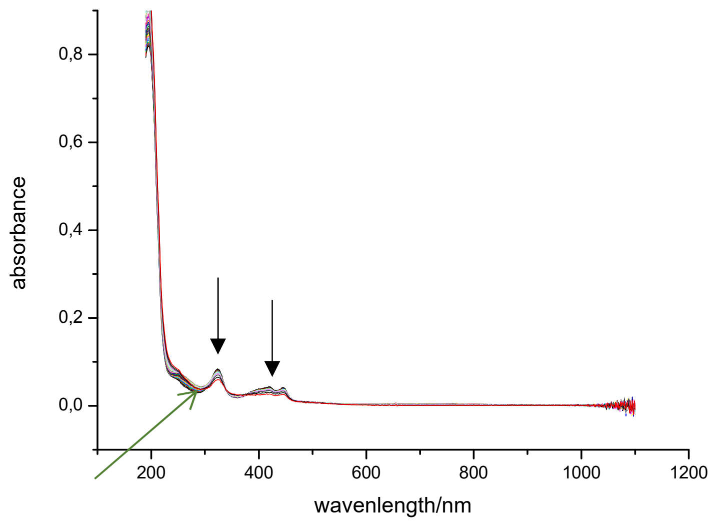

2nd peak (invers)

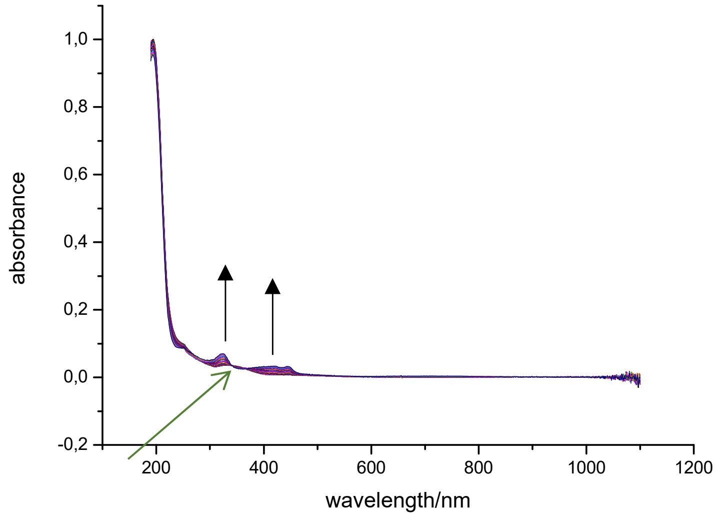

1st peak (invers)

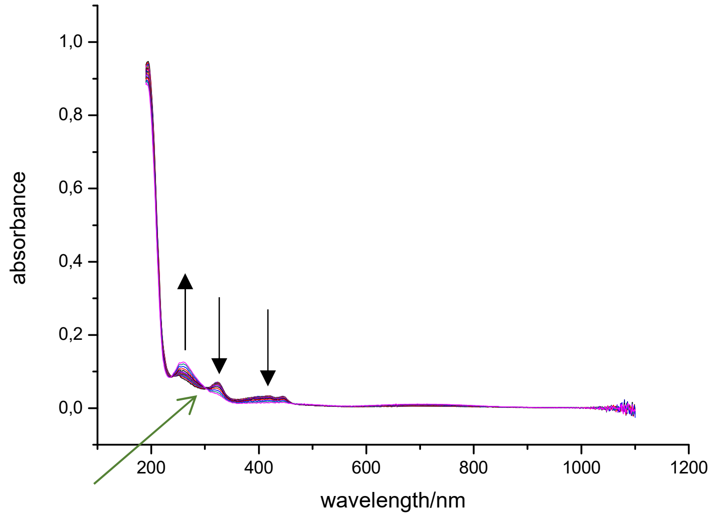

4.6. Compound **11**

- Cyclic Voltammetry

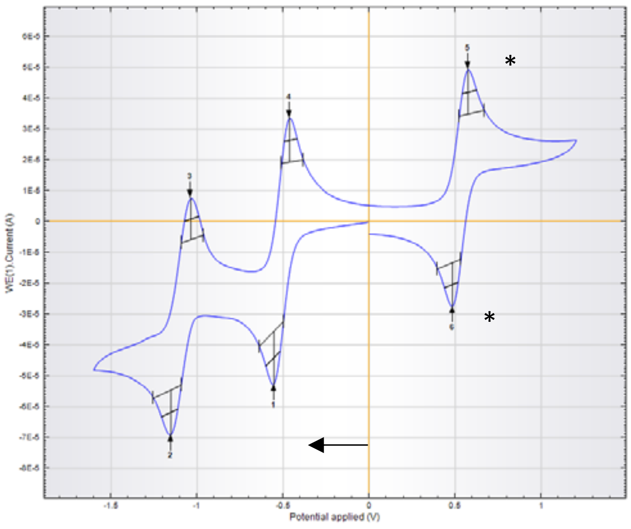

*Ferrocene as internal standard

- Spectroelectrochemical studies

Peak 1 (0- -0.7 V)

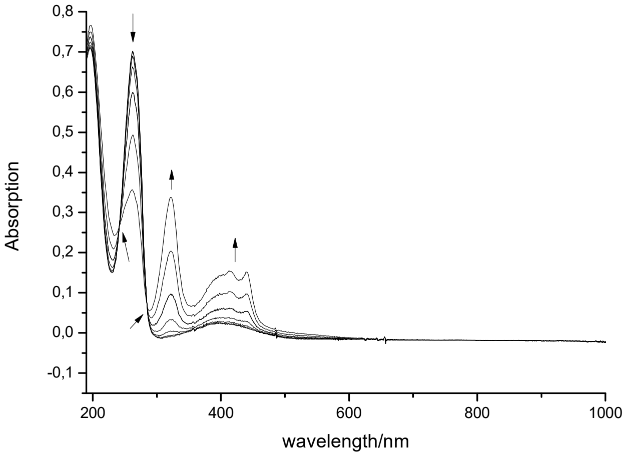

Peak 2 (-0.7- - 1.4V)

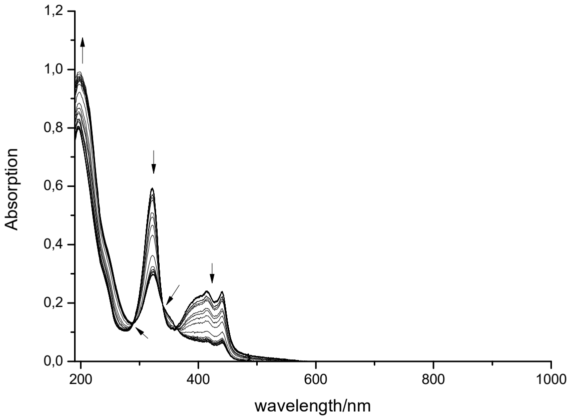

4.7. Compound **12**

- Cyclic Voltrammetry

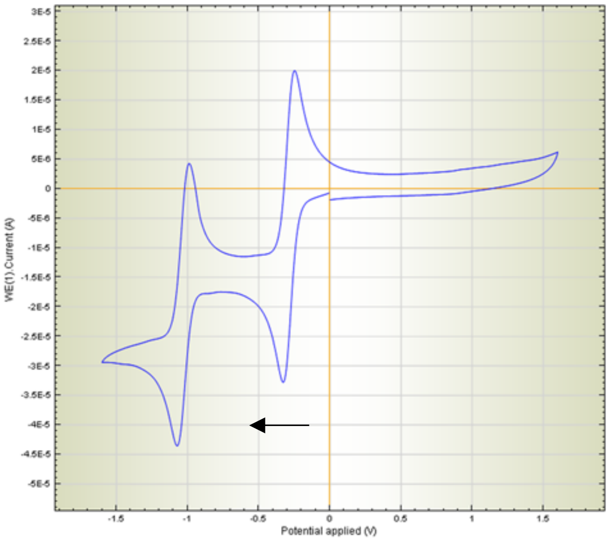

- Spectroelectrochemical studies

Peak 1 (0- -0.5V)

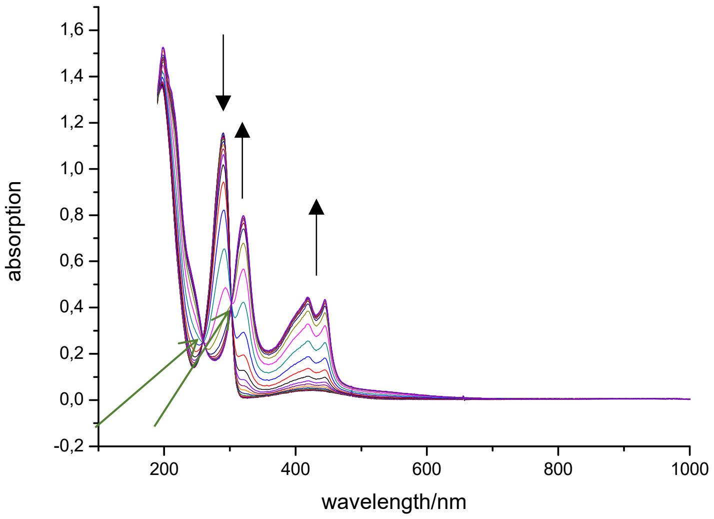

Peak 2 (-0.5 - -1.5V)

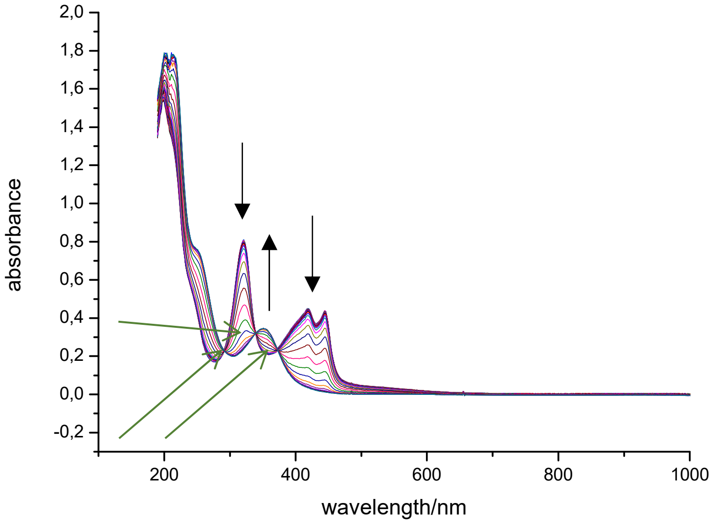

4.8. DCIP

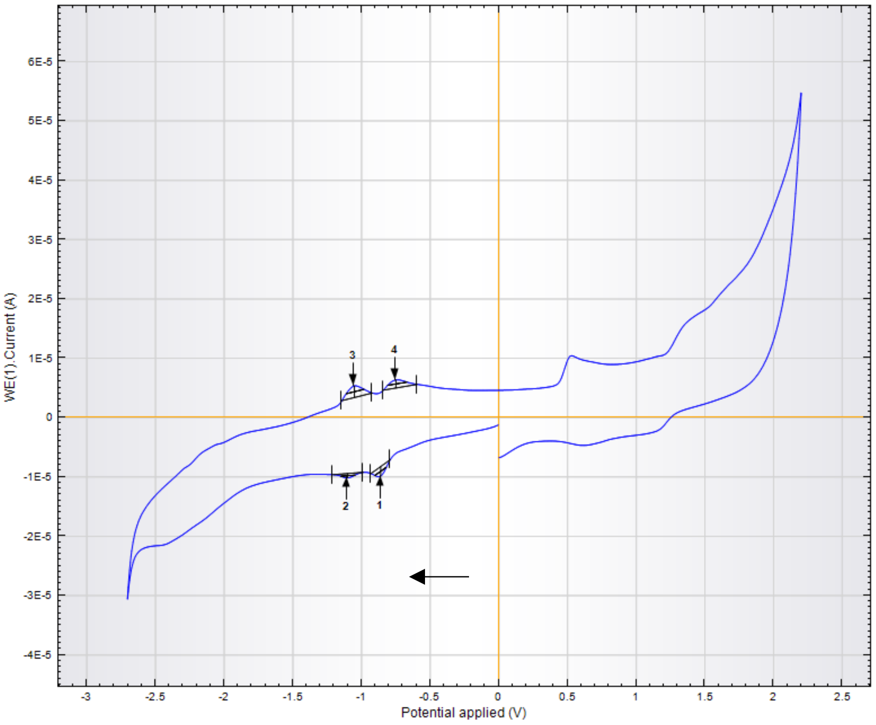

Potentials vs. SCN: -0.96 V, -1.23 V.

4.9. PMS

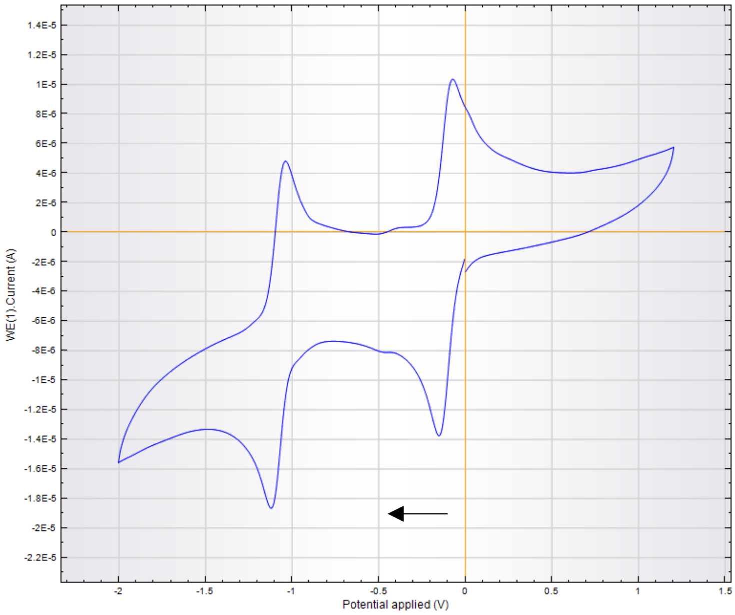

Potentials vs SCN: -0.11 V, -1.08 V.

5. Photoactivated Oxidation

5.1. Experimental Approach:

A Reduction of **1b**^{o}: A solution of **1b**^{o} (5 mM or 10 mM, DMSO, 600 μL) was dissolved in DMSO (1200 μL) and treated with a saturated solution of Hantzsch ester **13** (12 mM, DMSO, 600 μL) and stirred at ambient temperature in the dark.

To monitor the reaction, samples (100 μL) were taken and diluted with DMSO (100 μL), filtered and injected into analytical HPLC.

B Reduction of **1b**^{c}: A solution of **1b**^{c} (5 mM, DMSO, 50 μL) was dissolved in DMSO (100 μL) and treated with a saturated solution of Hantzsch ester **13** (12 mM, DMSO, 50 μL) and stirred at ambient temperature in the dark.

To monitor the reaction, samples (20 μL) were taken and diluted with DMSO (40 μL), filtered and injected into analytical HPLC.

C Reduction of **1b**^{c} and photoactivation: A solution of **1b**^{c} (10mM, DMSO, 50 μL) was dissolved in DMSO (100 μL) and treated with a saturated solution of Hantzsch ester **13** (12mM, DMSO, 50 μL) and stirred at ambient temperature in the dark. After three hours the sample was irradiated with a 590 nm single-spot LED for 10 min, then stirring was continued in the dark.

To monitor the reaction, samples (20 μL) were taken and diluted with DMSO (40 μL), filtered and injected into analytical HPLC.

5.2. Formation of **1b**^{o,red} using **1b**^{o} (10 mM, DMSO)

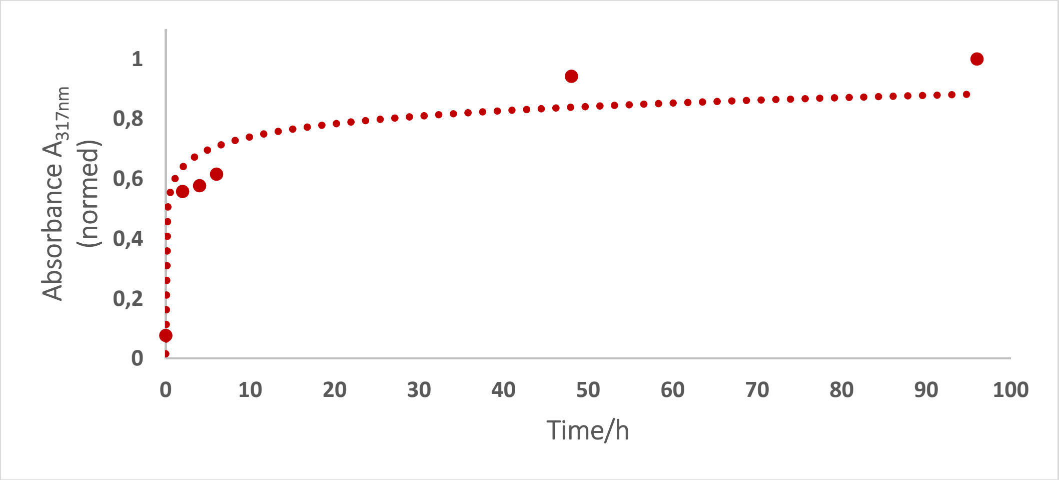

5.3. Representative HPLC traces (for Main Text, Figure 6 **A** and **B**)

Peaks: 7.2 min Hantzsch ester **13** (at the applied gradient oxidized and reduced form show the same retention time); 16.3 min: **1b**^{o,red}; 18.3 min: **1b**^{o}; 21.0 min: **1b**^{c}.

A_0h

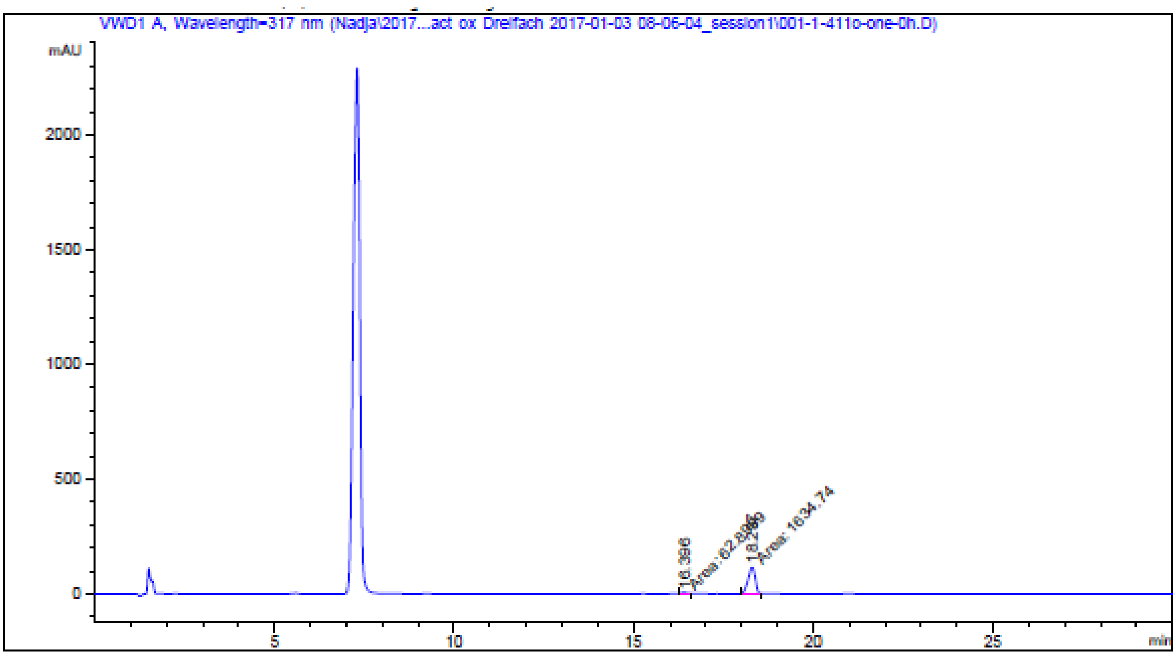


```
=====================================================================
                        Area Percent Report
=====================================================================

Sorted By             :      Signal
Multiplier            :      1.0000
Dilution              :      1.0000
Use Multiplier & Dilution Factor with ISTDs


Signal 1: VWD1 A, Wavelength=317 nm

Peak RetTime Type  Width     Area      Height     Area
  #   [min]        [min]   [mAU*s]     [mAU]        %
----|-------|----|-------|----------|----------|--------|
   1  16.396 MM    0.2133   62.89994    4.91378   3.7051
   2  18.297 MM    0.2337 1634.74426  116.57957  96.2949
```

A_11h

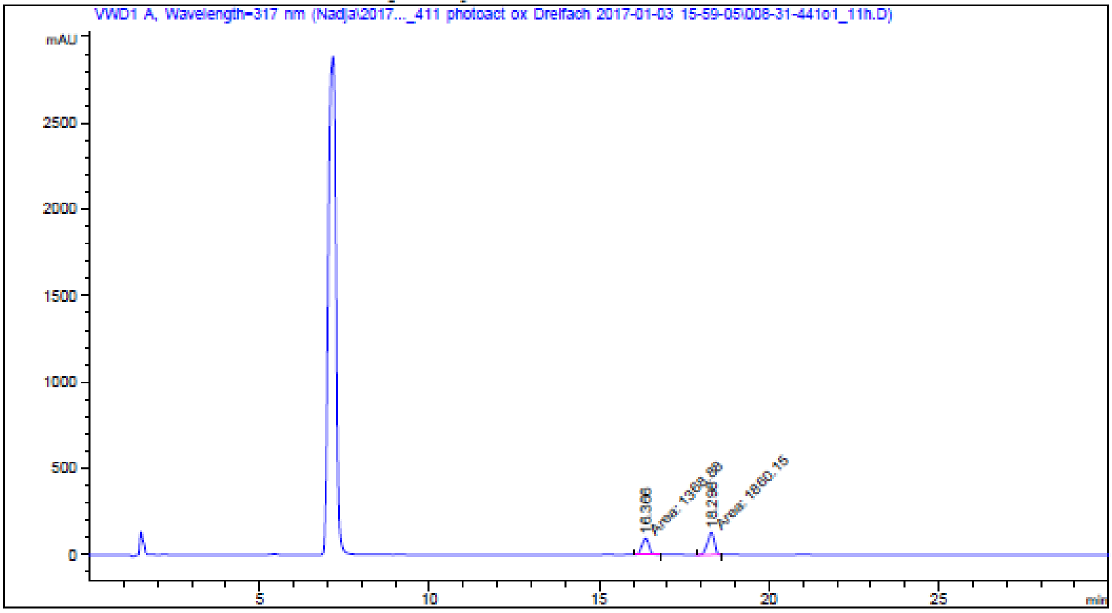


```
=====================================================================
                         Area Percent Report
=====================================================================

Sorted By             :      Signal
Multiplier            :      1.0000
Dilution              :      1.0000
Use Multiplier & Dilution Factor with ISTDs


Signal 1: VWD1 A, Wavelength=317 nm

Peak RetTime Type  Width     Area      Height     Area
  #   [min]        [min]   [mAU*s]     [mAU]        %
----|-------|----|-------|----------|----------|--------|
   1  16.366 MM    0.2389 1368.88477   95.50758  42.3930
   2  18.298 MM    0.2382 1860.14685  130.17055  57.6070
```

A_32h

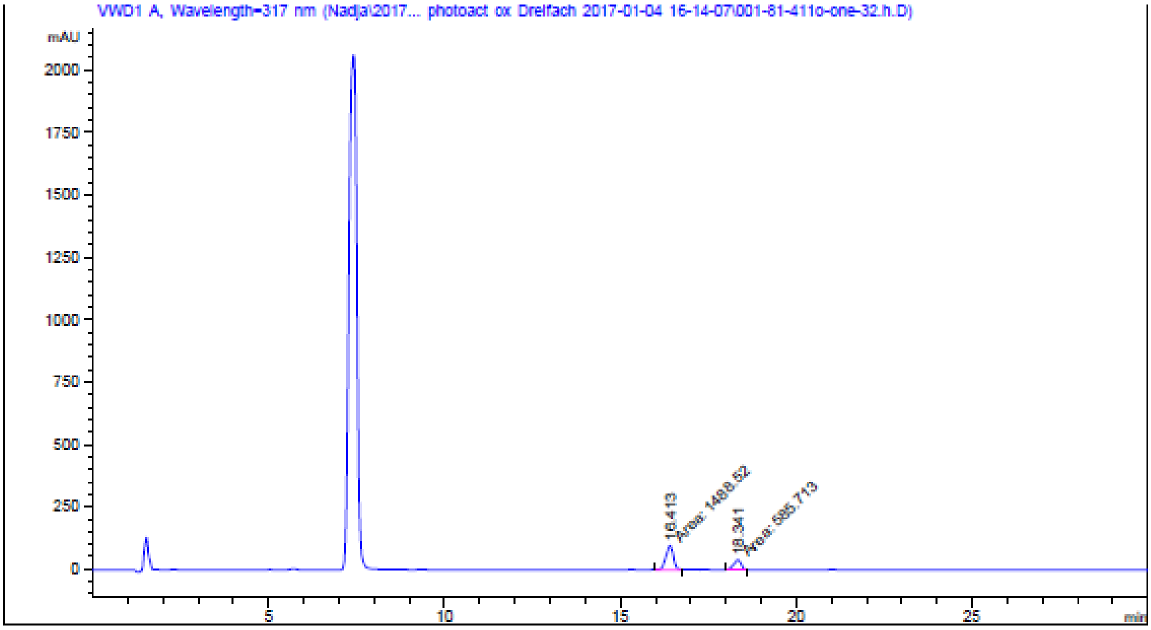

Area Percent Report

Sorted By	:	Signal
Multiplier	:	1.0000
Dilution	:	1.0000

Use Multiplier & Dilution Factor with ISTDs

Signal 1: VWD1 A, Wavelength=317 nm

Peak #	RetTime [min]	Type	Width [min]	Area [mAU*s]	Height [mAU]	Area %
1	16.413	MM	0.2525	1488.51843	98.24986	71.7624
2	18.341	MM	0.2508	585.71344	38.92433	28.2376

A_47h

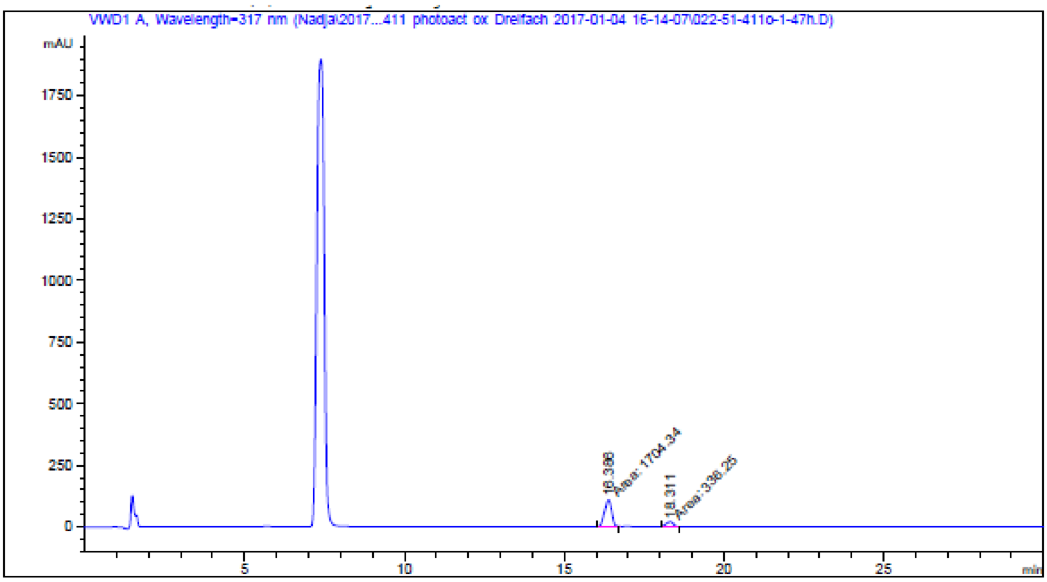

===
Area Percent Report
===

Sorted By : Signal
Multiplier : 1.0000
Dilution : 1.0000
Use Multiplier & Dilution Factor with ISTDs

Signal 1: VWD1 A, Wavelength=317 nm

Peak #	RetTime [min]	Type	Width [min]	Area [mAU*s]	Height [mAU]	Area %
1	16.386	MM	0.2598	1704.33765	109.34824	83.5219
2	18.311	MM	0.2752	336.25031	20.36510	16.4781

B_0h/C_0h

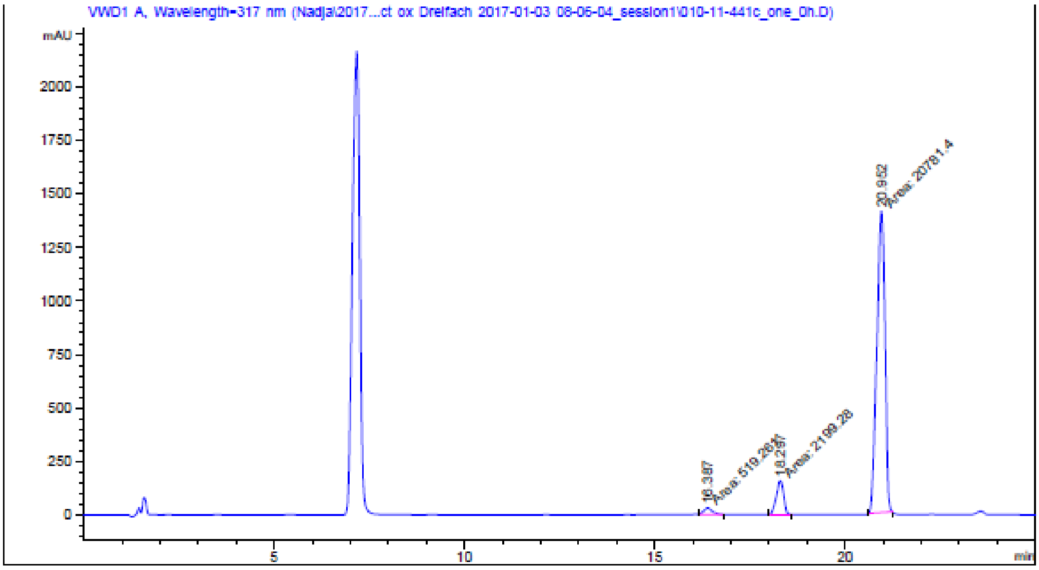


```
=====================================================================
                         Area Percent Report
=====================================================================

Sorted By             :      Signal
Multiplier            :      1.0000
Dilution              :      1.0000
Use Multiplier & Dilution Factor with ISTDs


Signal 1: VWD1 A, Wavelength=317 nm

Peak RetTime Type  Width     Area      Height     Area
  #   [min]        [min]   [mAU*s]     [mAU]        %
----|-------|----|-------|----------|----------|--------|
   1  16.387 MM    0.2781  519.26062   31.12502   2.2096
   2  18.297 MM    0.2289 2199.28003  160.10457   9.3587
   3  20.952 MM    0.2459 2.07814e4  1408.44507  88.4317
```

B_6h

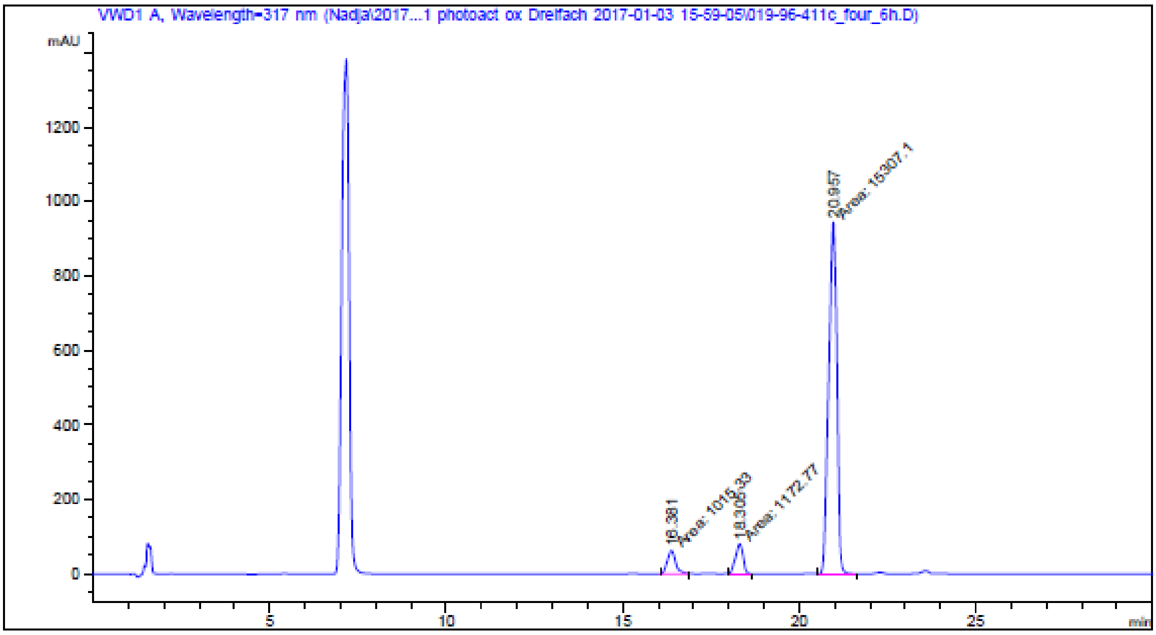


```
=====================================================================
                        Area Percent Report
=====================================================================

Sorted By             :      Signal
Multiplier            :      1.0000
Dilution              :      1.0000
Use Multiplier & Dilution Factor with ISTDs


Signal 1: VWD1 A, Wavelength=317 nm

Peak RetTime Type  Width     Area      Height     Area
  #   [min]        [min]   [mAU*s]     [mAU]        %
----|-------|----|-------|----------|----------|--------|
   1  16.381 MM    0.2752 1015.33301   61.48637   5.8035
   2  18.305 MM    0.2456 1172.76794   79.58045   6.7034
   3  20.957 MM    0.2697 1.53071e4   945.79211  87.4931
```

B_20h

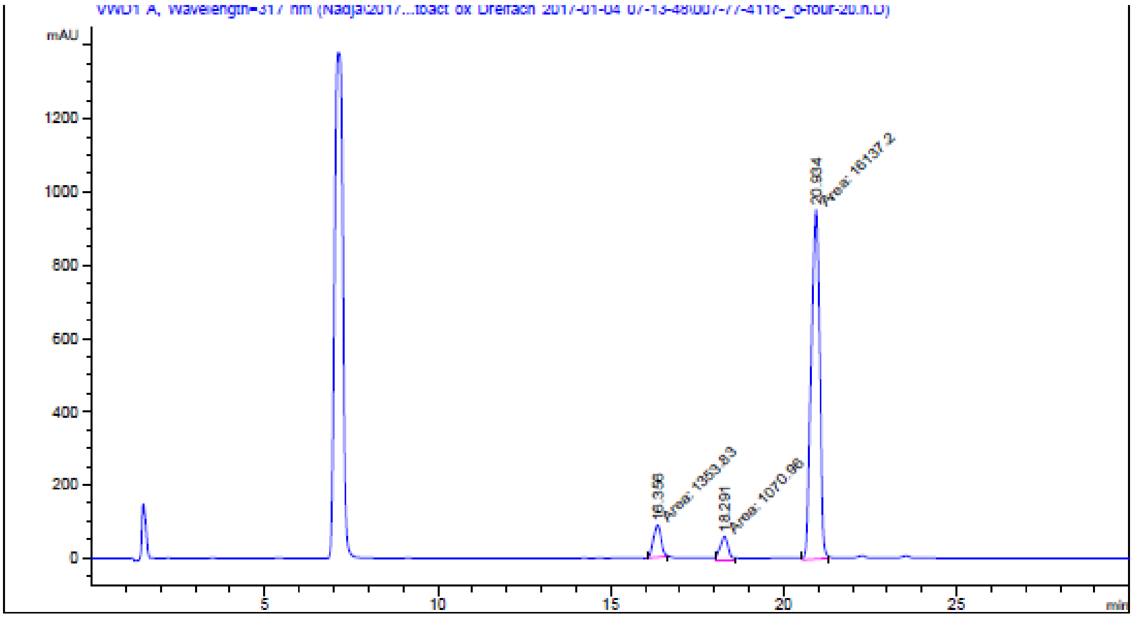


```
=====================================================================
                         Area Percent Report
=====================================================================

Sorted By             :      Signal
Multiplier            :      1.0000
Dilution              :      1.0000
Use Multiplier & Dilution Factor with ISTDs


Signal 1: VWD1 A, Wavelength=317 nm

Peak RetTime Type  Width     Area      Height     Area
  #   [min]        [min]   [mAU*s]     [mAU]       %
----|-------|----|-------|----------|----------|--------|
   1  16.356 MM    0.2586 1353.82556   87.26817   7.2935
   2  18.291 MM    0.2799 1070.96094   63.77544   5.7696
   3  20.934 MM    0.2822 1.61372e4   953.15631  86.9368
```

C_3h

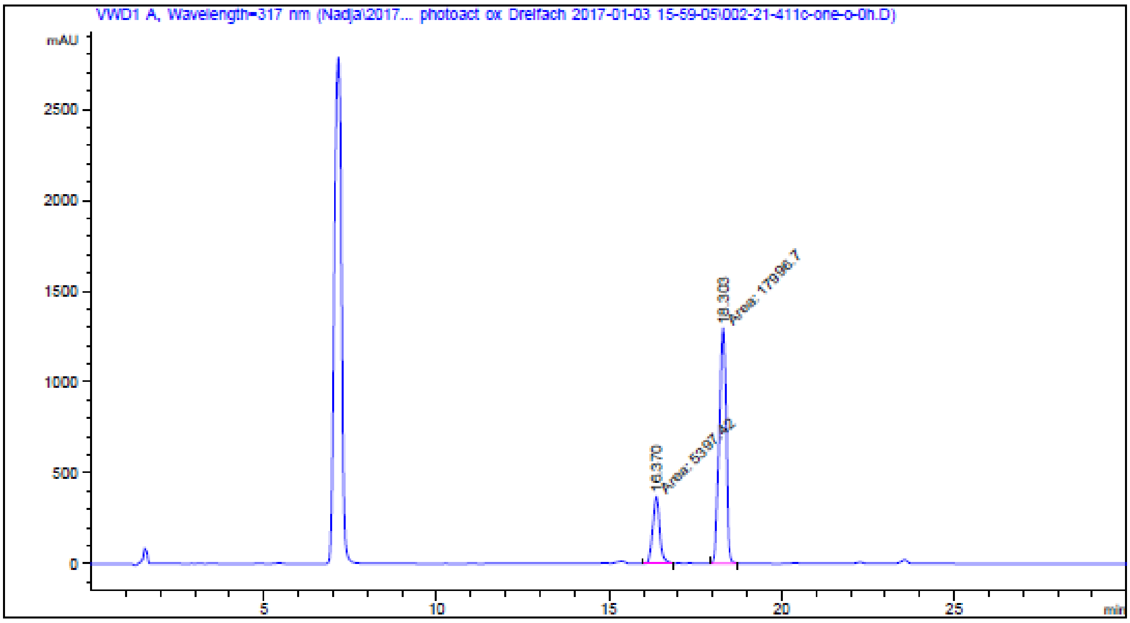


```
=====================================================================
                         Area Percent Report
=====================================================================

Sorted By             :      Signal
Multiplier            :      1.0000
Dilution              :      1.0000
Use Multiplier & Dilution Factor with ISTDs


Signal 1: VWD1 A, Wavelength=317 nm

Peak RetTime Type  Width     Area      Height     Area
  #   [min]        [min]   [mAU*s]     [mAU]       %
----|-------|----|-------|----------|----------|--------|
   1  16.370 MM     0.2431 5397.41650  370.02362  23.0717
   2  18.303 MM     0.2320 1.79967e4  1292.72192  76.9283
```

C_7.5h

Additional Info : Peak(s) manually integrated

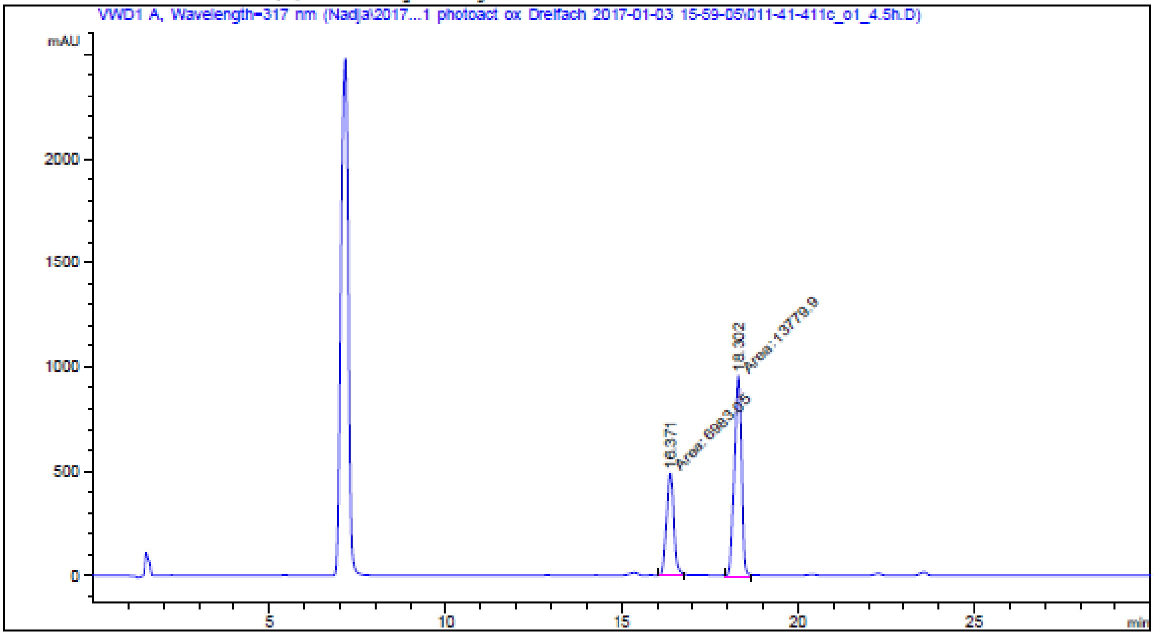


```
=====================================================================
                          Area Percent Report
=====================================================================

Sorted By             :      Signal
Multiplier            :      1.0000
Dilution              :      1.0000
Use Multiplier & Dilution Factor with ISTDs


Signal 1: VWD1 A, Wavelength=317 nm
```

Peak #	RetTime [min]	Type	Width [min]	Area [mAU*s]	Height [mAU]	Area %
1	16.371	MM	0.2390	6983.05029	486.93979	33.6323
2	18.302	MM	0.2388	1.37799e4	961.71655	66.3677

C_39h

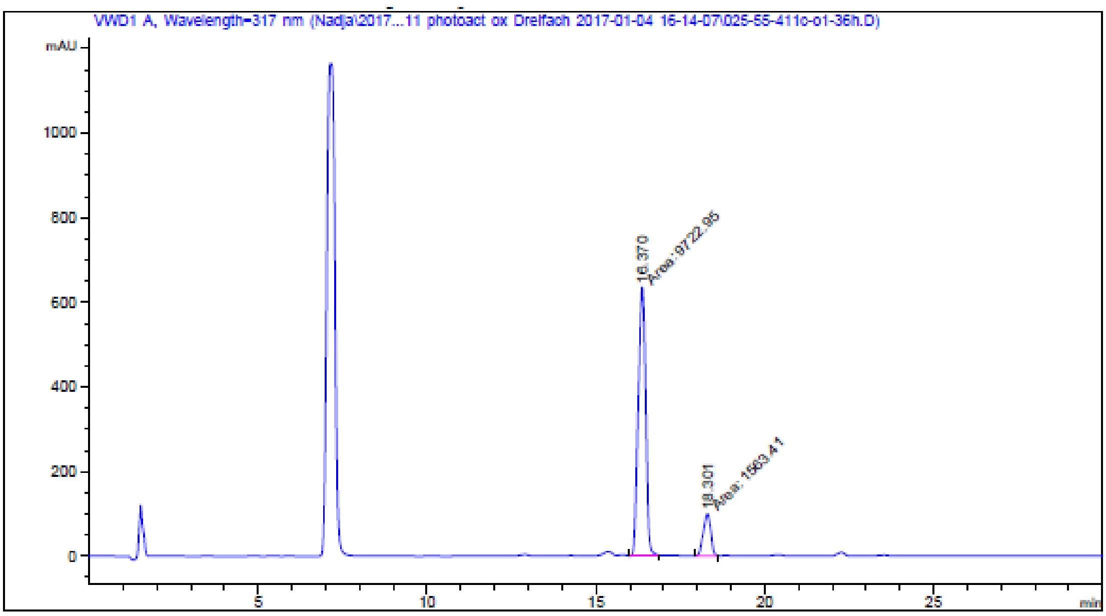


```
=====================================================================
                         Area Percent Report
=====================================================================

Sorted By             :      Signal
Multiplier            :      1.0000
Dilution              :      1.0000
Use Multiplier & Dilution Factor with ISTDs


Signal 1: VWD1 A, Wavelength=317 nm

Peak RetTime Type  Width     Area      Height     Area
  #   [min]        [min]   [mAU*s]     [mAU]       %
----|-------|----|-------|----------|----------|--------|
   1  16.370 MM    0.2555 9722.95117  634.16931  86.1478
   2  18.301 MM    0.2608 1563.40955   99.90733  13.8522
```

6. Activation of **1b**^{c} on isolated Mitochondria

- Stability of **1b**^{c} at elevated temperatures

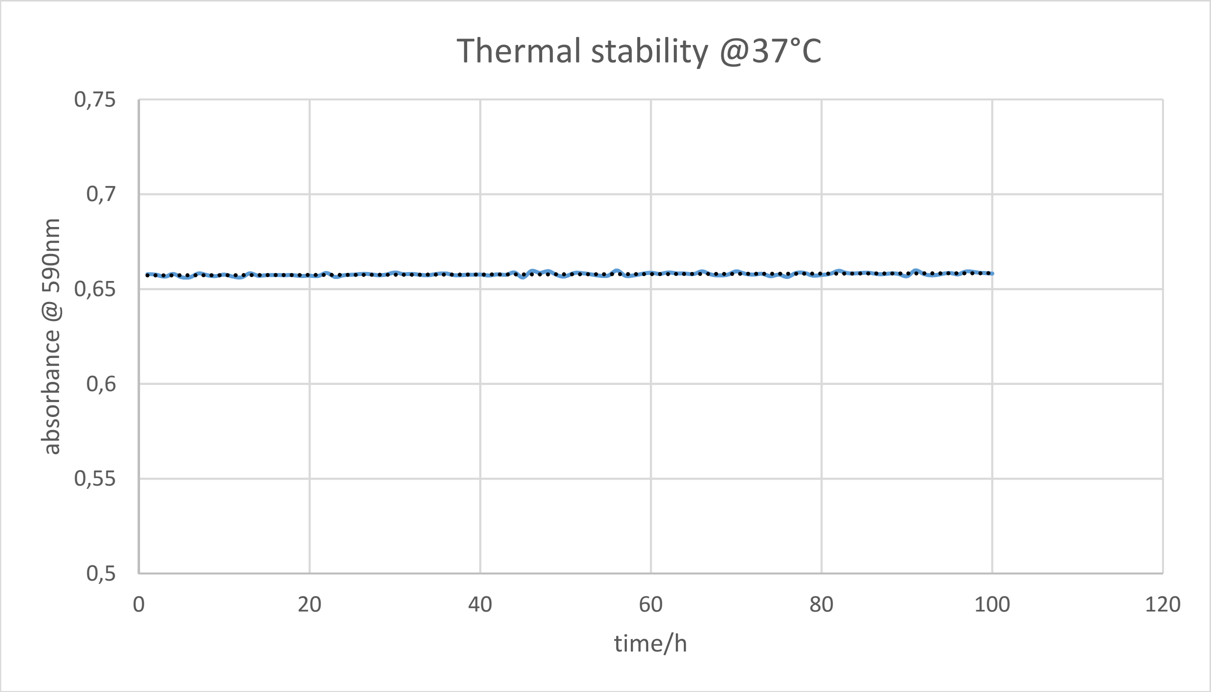

- Purity of compounds **1b**^{o} and **1b**^{c}

1b^{o}

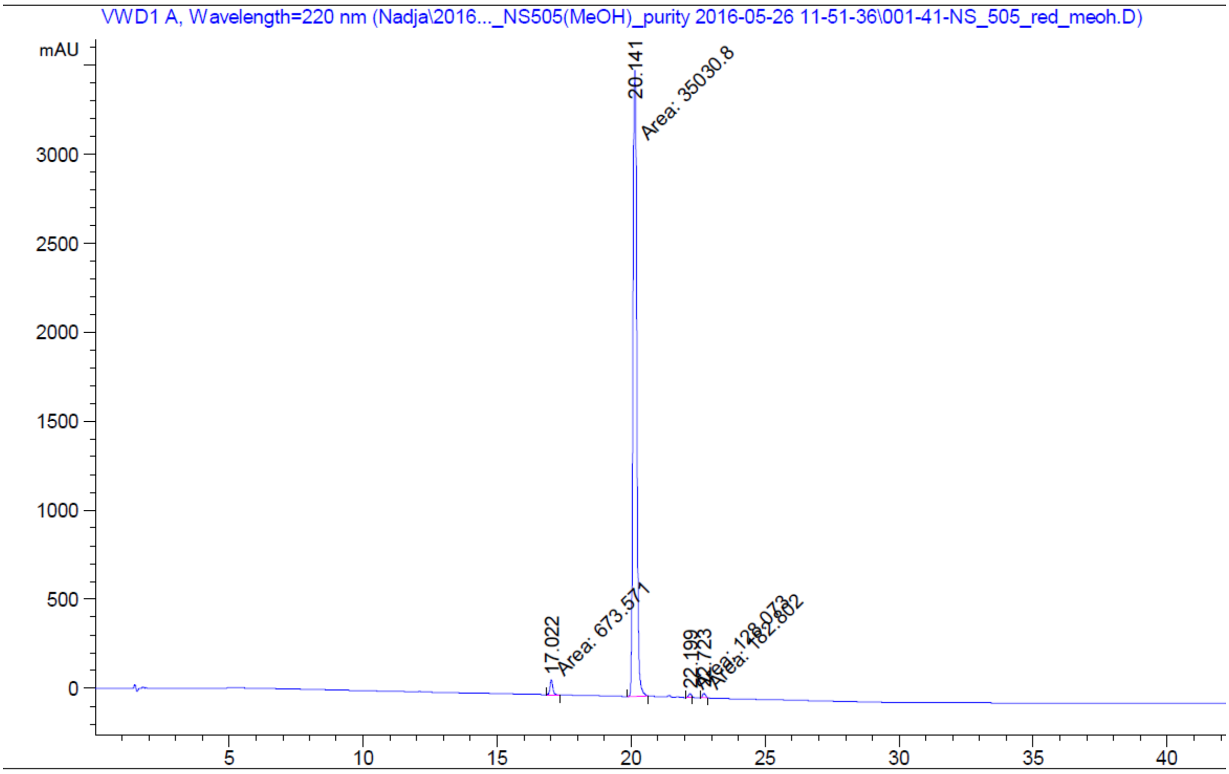

Peak #	RetTime [min]	Type	Width [min]	Area [mAU*s]	Height [mAU]	Area %
1	17.022	MM	0.1302	673.57086	86.23805	1.8702
2	20.141	MM	0.1661	3.50308e4	3515.66992	97.2666
3	22.199	MM	0.1065	128.07347	20.05158	0.3556
4	22.723	MM	0.1231	182.80190	24.75809	0.5076

1b^{c}

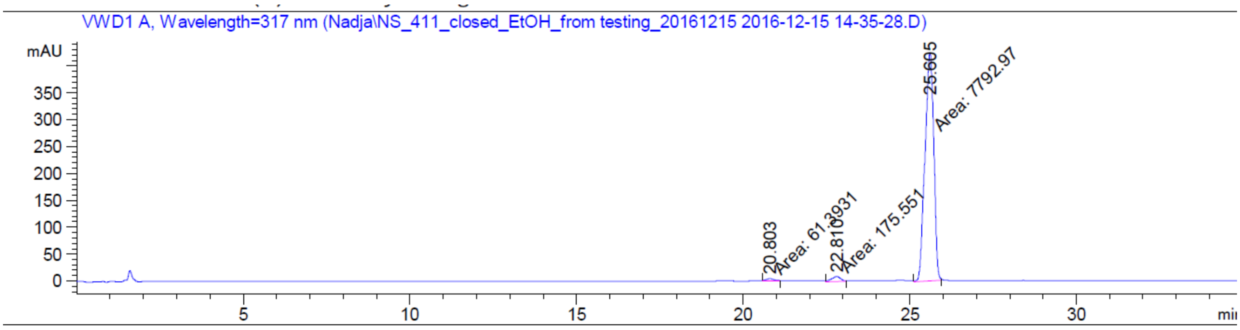

Peak #	RetTime [min]	Type	Width [min]	Area [mAU*s]	Height [mAU]	Area %
1	20.803	MM	0.2713	61.39307	3.77112	0.7646
2	22.810	MM	0.3193	175.55141	9.16268	2.1862
3	25.605	MM	0.3063	7792.96826	424.01196	97.0492
Totals :				8029.91273	436.94577	

- Isolation of mitochondria from yeast cells

A crude mitochondria extract was isolated from the wildtype yeast strain BY4742 (MATα his3Δ1 leu2Δ0 lys2Δ0 ura3Δ0), thankfully provided by the group of Herbert Tschochner, according to Gregg et al.[3] The protein concentration of the extract was determined with the Bradford assay[4] and the mitochondria were stored in 30 mM KP (pH 7.4), 2.2 mM EDTA and 0.1% (w/v) Thesit at -80 °C

- Mitochondrial reduction of 2,6-dichlorophenol indophenol

Reduction of 2,6-dichlorophenol indophenol (DCIP) was measured using the succinate dehydrogenase activity (SDH) of mitochondria as described previously.[5] Prior to the assays, SDH was activated with 8 mM succinate for 15 min at 37 °C. Triplet measurements were carried out in a 96-well plate of assays containing 40 μM **1b**^{c} and **1b**^{o} dissolved in ethanol, respectively, 10 mM succinate, 100 μM DCIP and 0.2 g/L mitochondria in 50 mM KP (pH 7.6), 0.006% (w/v) Thesit, 0.2 mM EDTA at 37 °C. Reduction of DCIP was followed at 520 nm ($\varepsilon_{DCIP,520}$ = 6.8 mM^{-1} cm^{-1}) continuously for 30 min or with 10 min interruption in which the samples were irradiated with 590 nm in the same plate reader used for activity measurements with an integrated 10 W xenon flash lamp (Tecan Trading AG). The resulting data were plotted and fitted with a linear function in the time frame of 0-10 min as well as 20-30 min to visualize the difference in reactivity.

7. Computational Studies

All DFT-calculations (B3LAP/6–31G(d) level) were performed with the Gaussian 09, Revision D01 software package.[6] Optimized structures (**1b**$^{o}_{min}$, **1b**$^{c}_{min}$, **13**$_{min}$, {**1b**$^{o}_{min}$–**13**$_{min}$}$_{ADJ}$, {**1b**$^{c}_{min}$–**13**$_{min}$}$_{ADJ}$) were confirmed to be minima through frequency calculations. Single-point energies were calculated for **1b**$^{o}_{ADJ}$, **1b**$^{c}_{ADJ}$, **13**$_{ADJ}$, {**1b**$^{o}_{min}$–**13**$_{min}$}$_{nonADJ}$ and {**1b**$^{c}_{min}$–**13**$_{min}$}$_{nonADJ}$. All cartesian coordinates are given in paragraph 10. later in the Supporting Information.

Adapting a published strategy, an energy cycle to gain insight into the presence of a non-covalent π stacking between both photoisomers and **13** was calculated according to the following scheme.[7]

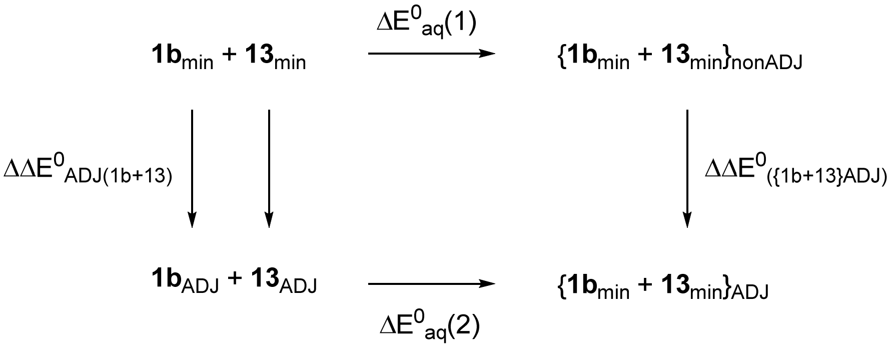

Then, the energies of the optimized structures (indicated with “min”) of both photoisomers, the Hantzsch ester and the respective adduct complexes were compared with single point calculations of mixed structures, revealing the adjustment process (indicated with “ADJ”) of the structures upon π stacking. The energy differences are given as follows:

	**1b**^{o}	**1b**^{c}	$\Delta\Delta E^0$(**1b**^{c}-**1b**^{o})
$\Delta E^0_{aq}(1)$*	-0,38	1,38	
$\Delta E^0_{aq}(2)$*	-16,5	-22,9	
$\Delta\Delta EG^0_{ADJ(1b+13)}$*	1,07	1,75	
$\Delta\Delta E^0_{(\{1b+13\}ADJ)}$*	-15,06	-22,53	-7,47

*Energy differences are expressed in Kcal/mol.

8. NMR spectra

- Compound **1a**[o]

^{1}H NMR ($CDCl_3$, 400 MHz)

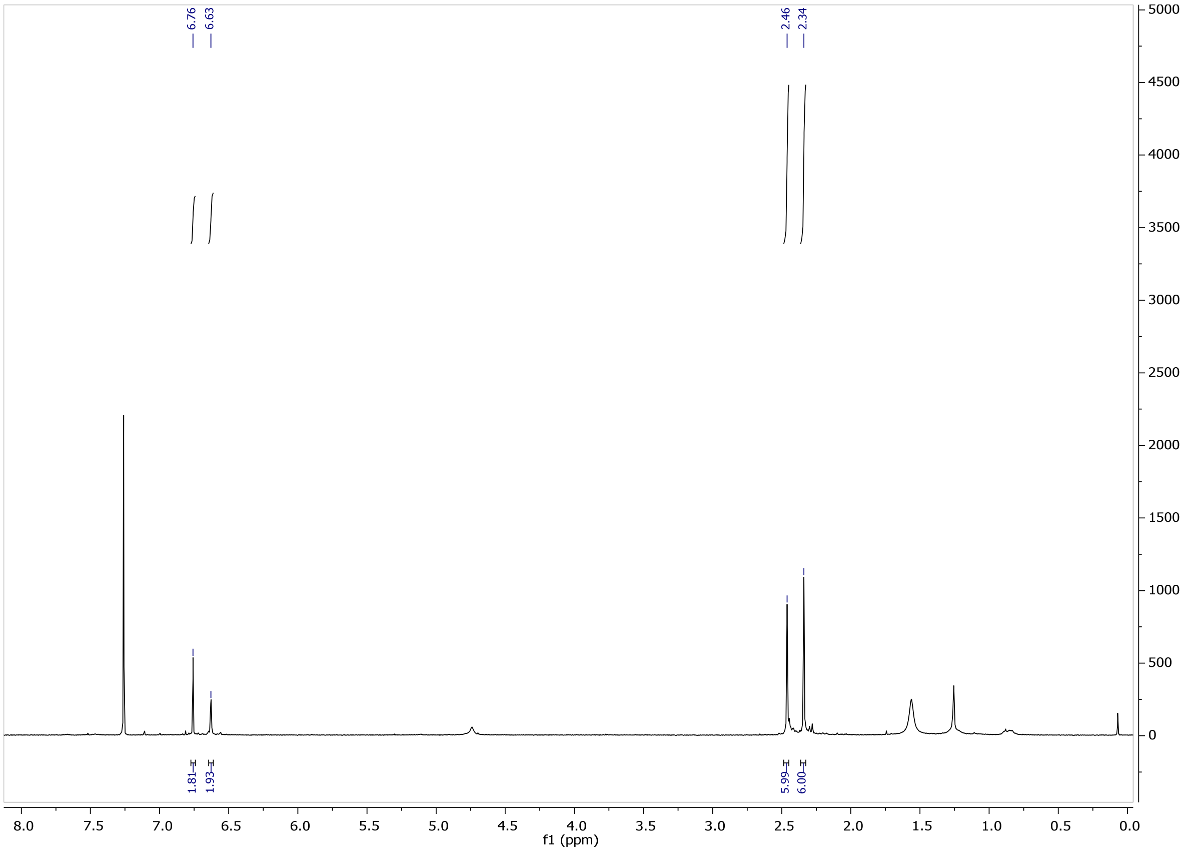

^{13}C NMR ($CDCl_3$, 101 MHz)

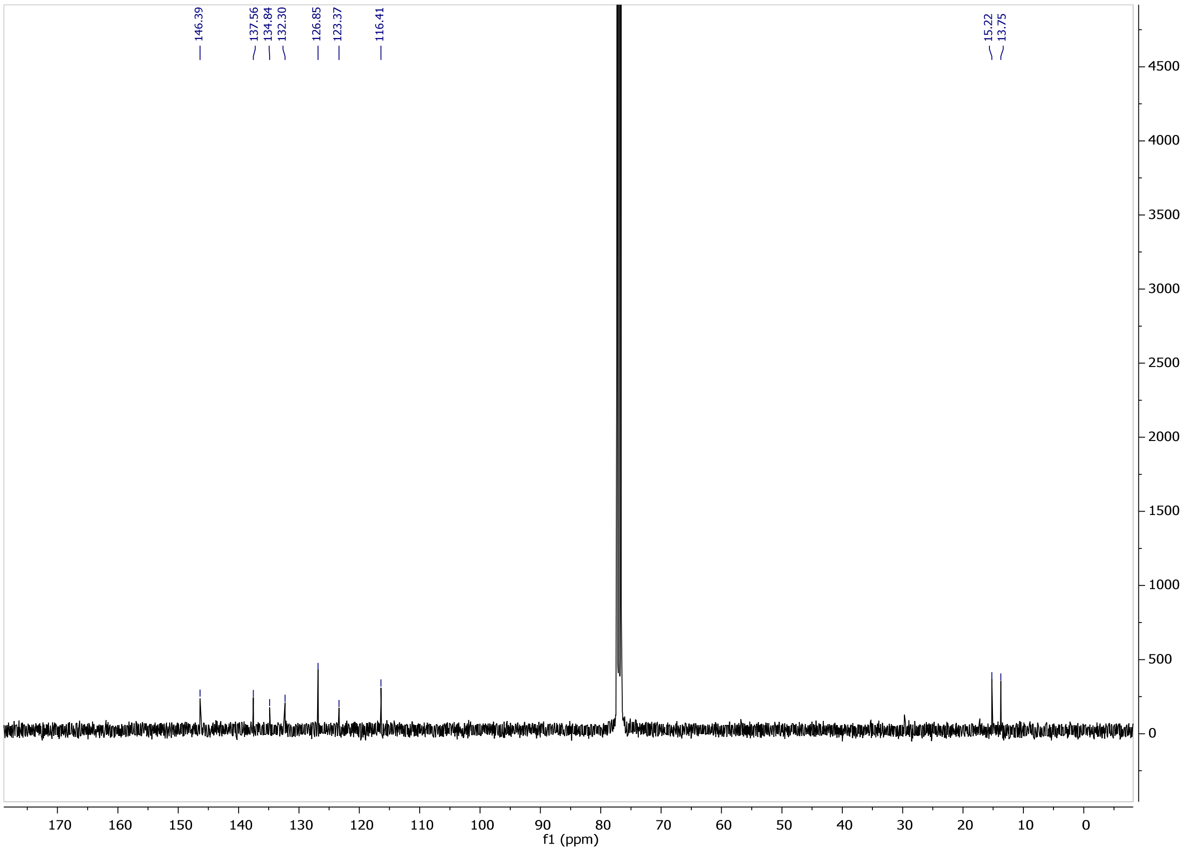

- Compound **1b'**

^{1}H ($CDCl_3$, 600 MHz)

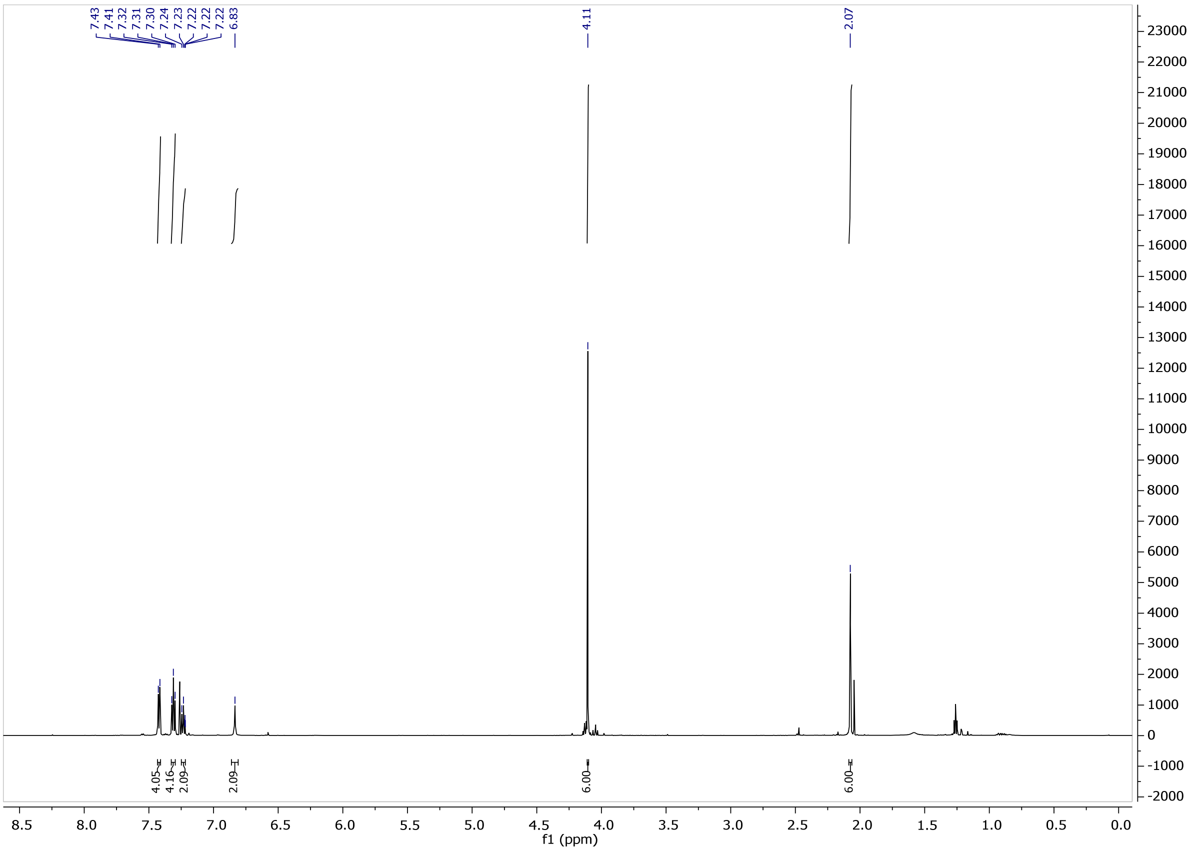

^{13}C ($CDCl_3$, 151 MHz)

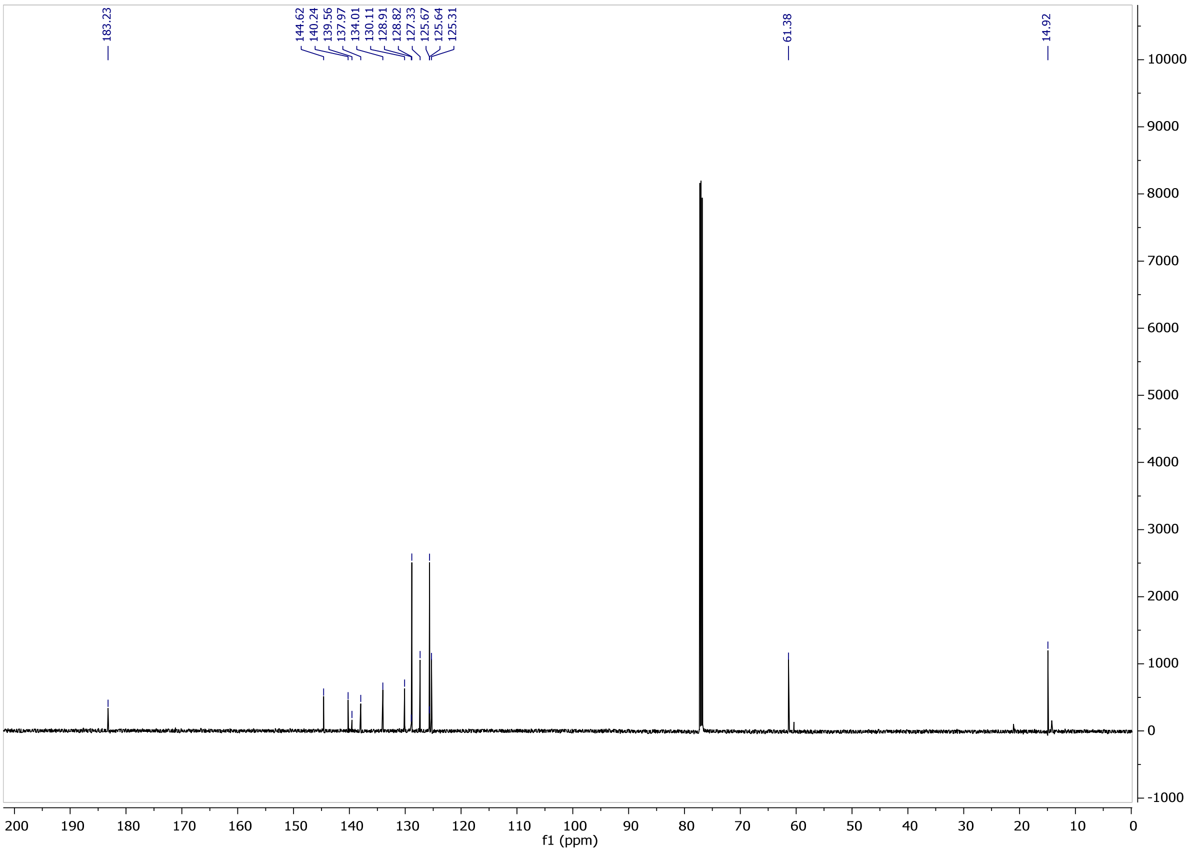

- Compound **1b**[c]

^1H NMR($CDCl_3$, 400MHz)

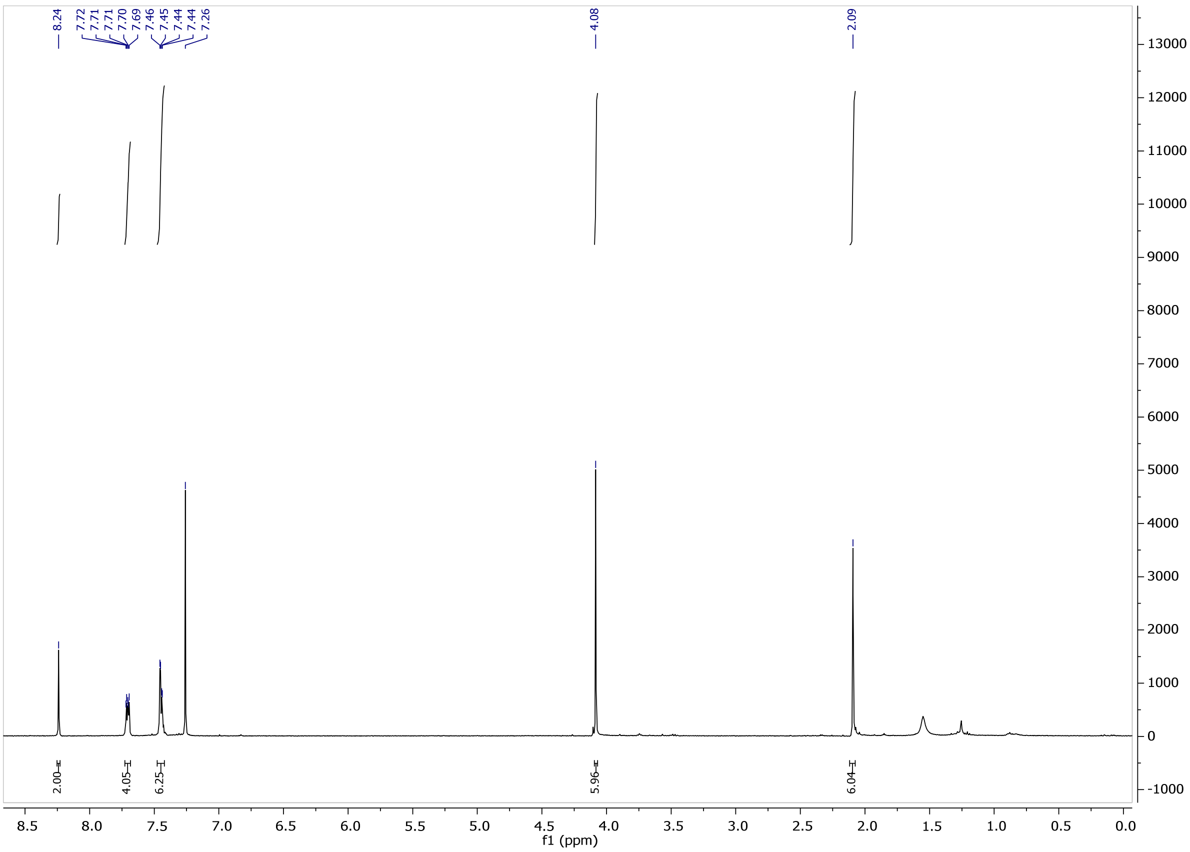

- Compound **9b**

^1H ($CDCl_3$, 300 MHz)

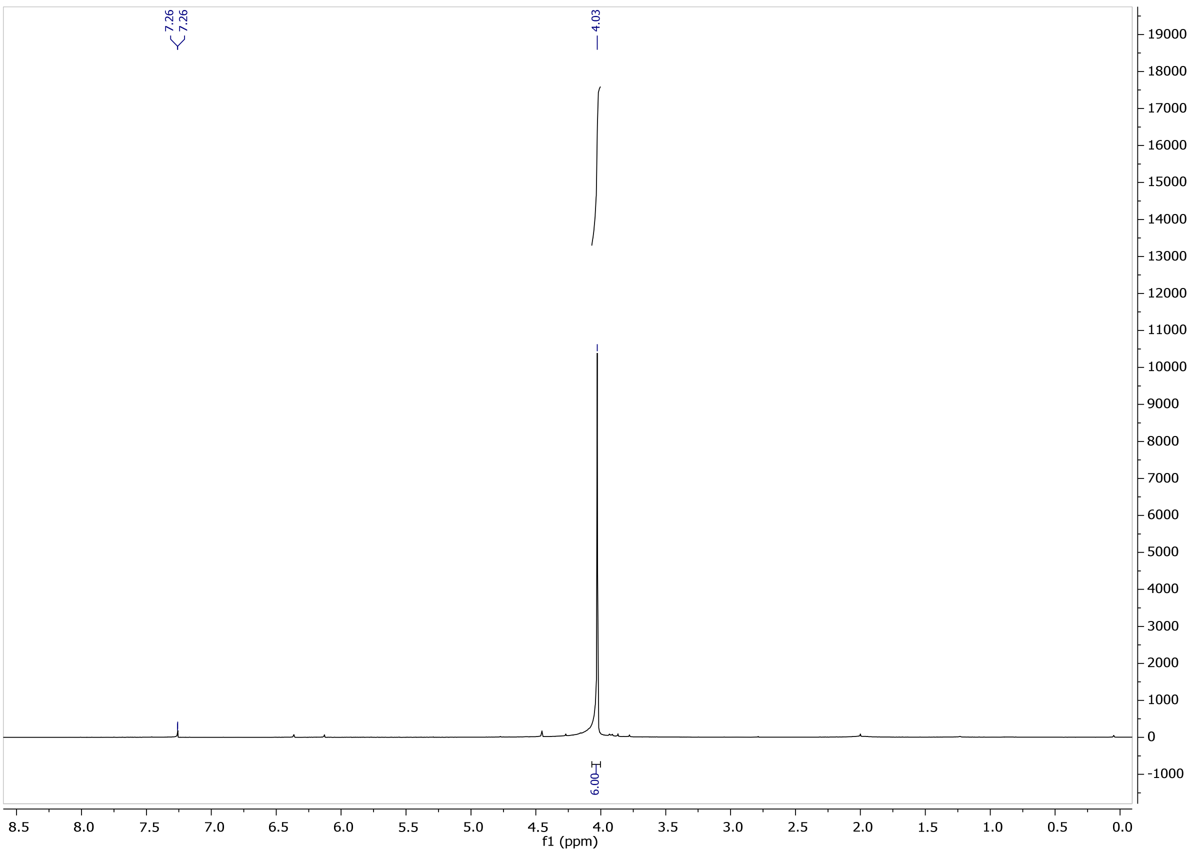

^{13}C NMR ($CDCl_3$, 75MHz)

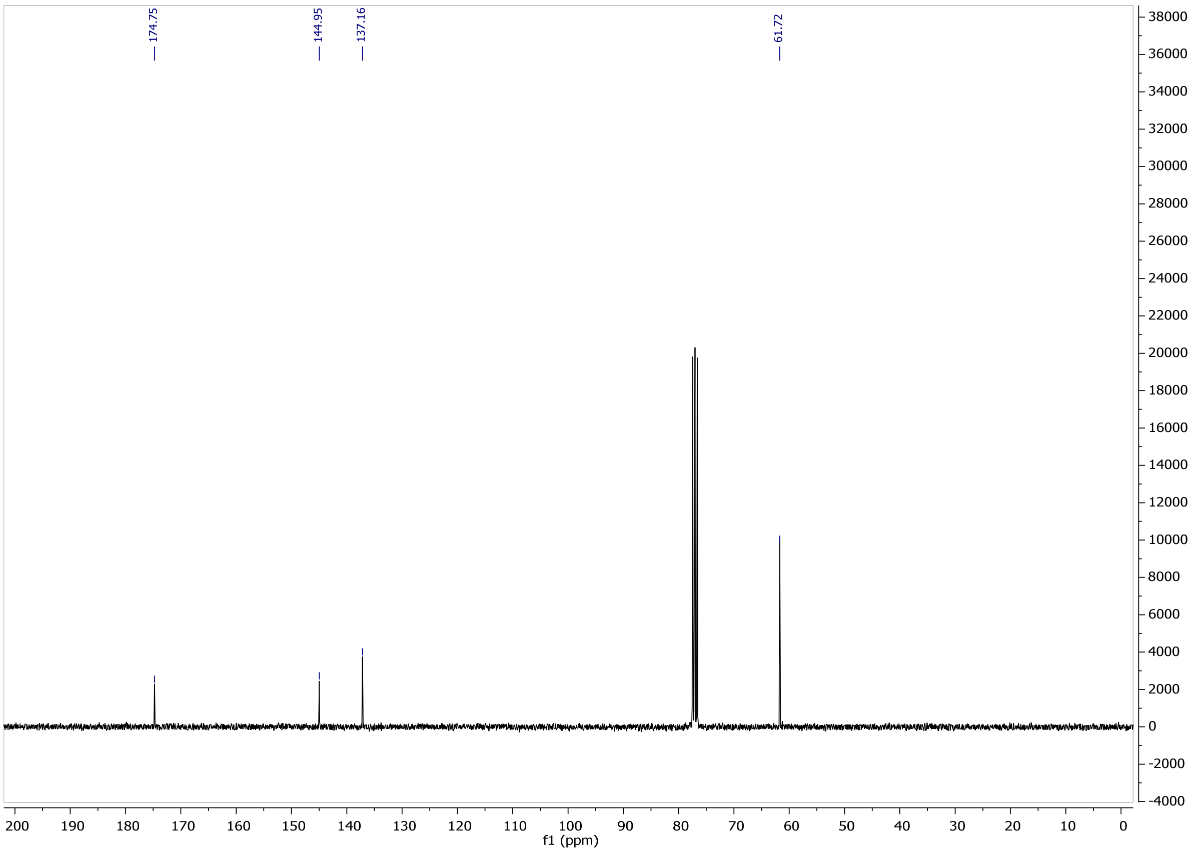

9. X-Ray Structure of 9b

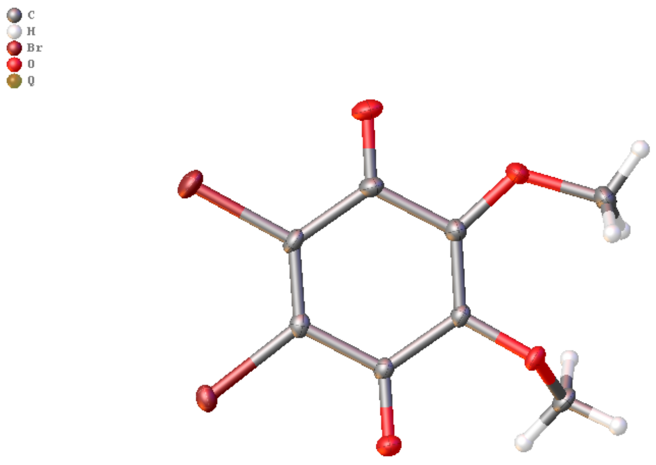

**Compound**	**O076**
Formula	$C_8H_6Br_2O_4$
$D_{calc.}$/ g cm^{-3}	2.248
□/mm^{-1}	10.606
Formula Weight	325.95
Colour	light red
Shape	block
Size/mm^3	0.25×0.18×0.10
T/K	123.01(10)
Crystal System	monoclinic
Space Group	$P2_1/c$
a/Å	8.56023(6)
b/Å	16.31960(16)
c/Å	6.91885(5)
□/°	90
□/°	94.8033(7)
□/°	90
V/Å^3	963.165(14)
Z	4
Z'	1
Wavelength/Å	1.54184
Radiation type	CuK□
□$_{min}$/°	5.185
□$_{max}$/°	73.474
Measured Refl.	13822
Independent Refl.	1927
Reflections Used	1907
R_{int}	0.0288
Parameters	129
Restraints	0
Largest Peak	0.519
Deepest Hole	-0.959
GooF	1.195
wR_2 (all data)	0.0687
wR_2	0.0685
R_1 (all data)	0.0276
R_1	0.0271

Experimental. Single light red block-shaped crystals of (**O076**) were obtained by recrystallisation from .... A suitable crystal (0.25×0.18×0.10) mm^3 was selected and mounted on ... on a GV1000, TitanS2 diffractometer. The crystal was kept at T = 123.01(10) K during data collection. Using **Olex2**, the structure was solved with the **ShelXT** structure solution program, using the Direct Methods solution method. The model was refined with version 2016/6 of **ShelXL** using Least Squares minimisation.[8]

Crystal Data. $C_8H_6Br_2O_4$, M_r = 325.95, monoclinic, $P2_1/c$ (No. 14), a = 8.56023(6) Å, b = 16.31960(16) Å, c = 6.91885(5) Å, □ = 94.8033(7)°, □ = □ = 90°, V = 963.165(14) Å^3, T = 123.01(10) K, Z = 4, Z' = 1, □(CuK□) = 10.606, 13822 reflections measured, 1927 unique (R_{int} = 0.0288) which were used in all calculations. The final wR_2 was 0.0687 (all data) and R_1 was 0.0271 (I > 2(I)).

Structure Quality Indicators

**Reflections:**	d min (Cu) 0.80	I/σ 55.4	Rint 2.88%	complete at 2θ=134° 99%
**Refinement:**	Shift -0.001	Max Peak 0.5	Min Peak -1.0	GooF 1.195

A light red block-shaped crystal with dimensions 0.25×0.18×0.10 mm³ was mounted on ....X-ray diffraction data were collected using a GV1000, TitanS2 diffractometer equipped with a n/a low-temperature device, operating at T = 123.01(10) K.

Data were measured using □ scans scans of 1.0 ° per frame for 1.0 s using CuK□ radiation (gradient vaccum rotating-anode X-ray tube, n/a kV, n/a mA). The total number of runs and images was based on the strategy calculation from the program CrysAlisPro (Agilent).The maximum resolution achieved was □ = 73.474. °

Cell parameters were retrieved using the CrysAlisPro (Agilent) software and refined using CrysAlisPro (Agilent) on 11290 reflections, 82 % of the observed reflections. Data reduction was performed using the CrysAlisPro (Agilent) software which corrects for Lorentz polarisation. The final completeness is 99.90 out to 73.474 in □. The absorption coefficient □ of this material is 10.606 at this wavelength (□ = 1.54184) and the minimum and maximum transmissions are 0.634 and 0.800.

The structure was solved in the space group P2$_1$/c (# 14) by Direct Methods using the **ShelXT** (Sheldrick, 2015) structure solution program and refined by Least Squares using version 2016/6 of **ShelXL** (Sheldrick, 2015). All non-hydrogen atoms were refined anisotropically. Hydrogen atom positions were calculated geometrically and refined using the riding model.

_refine_special_details: ?

_exptl_absorpt_special_details: ?

There is a single molecule in the asymmetric unit, which is represented by the reported sum formula. In other words: Z is 4 and Z' is 1.

Data Plots: Diffraction Data

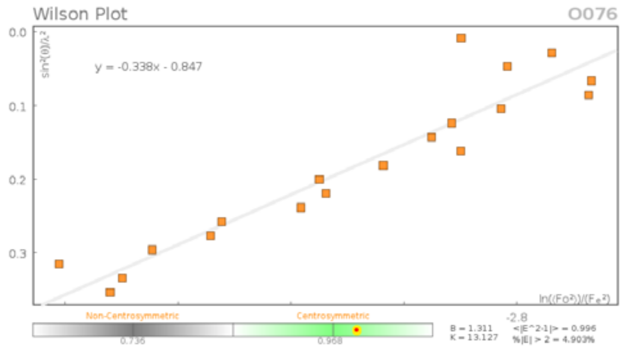

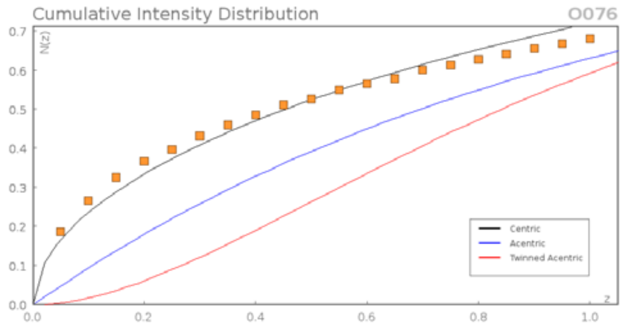

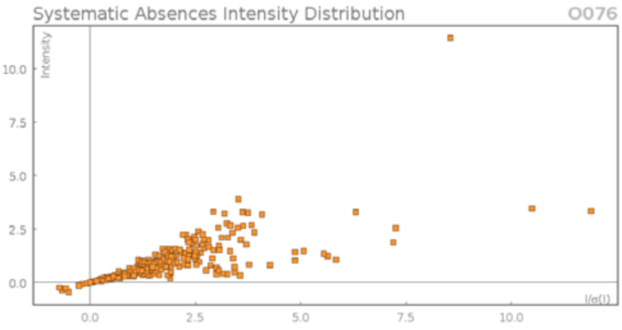

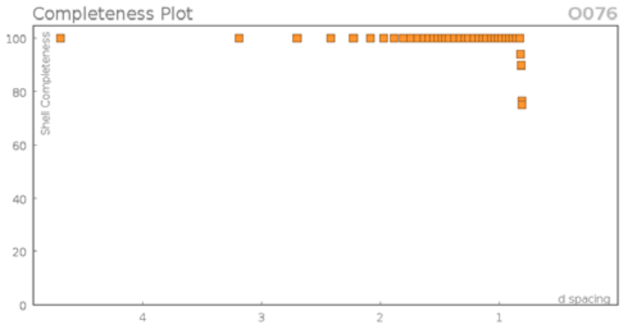

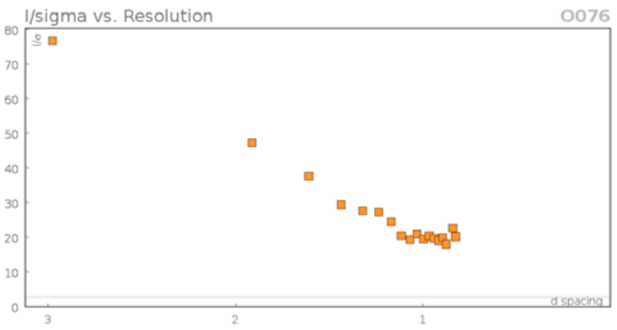

Data Plots: Refinement and Data

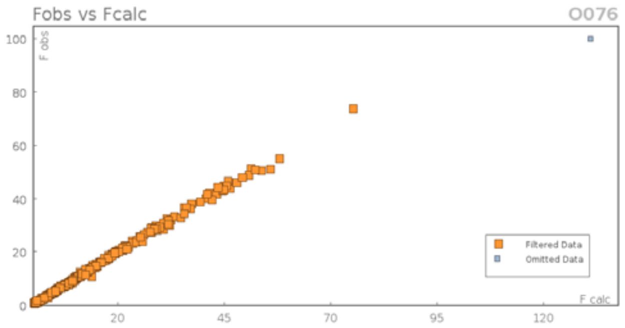

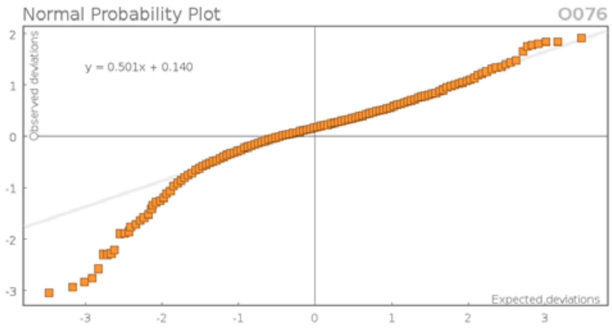

Reflection Statistics

Total reflections (after filtering)	14182	Unique reflections	1927
Completeness	0.993	Mean I/□	55.39
hkl_{max} collected	(10, 19, 8)	hkl_{min} collected	(-10, -19, -8)
hkl_{max} used	(10, 19, 8)	hkl_{min} used	(-10, 0, 0)
Lim d_{max} collected	100.0	Lim d_{min} collected	0.77
d_{max} used	8.53	d_{min} used	0.8
Friedel pairs	2829	Friedel pairs merged	1
Inconsistent equivalents	15	R_{int}	0.0288
R_{sigma}	0.0129	Intensity transformed	0
Omitted reflections	0	Omitted by user (OMIT hkl)	1
Multiplicity	(2411, 2142, 1154, 559, 246, 79, 11, 1)	Maximum multiplicity	20
Removed systematic absences	359	Filtered off (Shel/OMIT)	0

Images of the Crystal on the Diffractometer

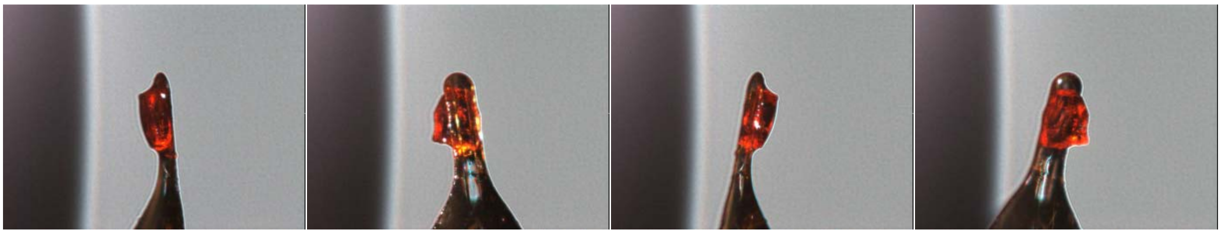

Table 1: Fractional Atomic Coordinates (×10⁴) and Equivalent Isotropic Displacement Parameters (Å²×10³) for **O076**. U_{eq} is defined as 1/3 of the trace of the orthogonalised U_{ij}.

Atom	x	y	z	U_{eq}
Br(01)	3602.1(3)	4478.8(2)	2540.8(4)	17.37(11)
Br(02)	6514.2(4)	3063.3(2)	2723.1(5)	24.33(11)
O(003)	5017(2)	6143.7(12)	2295(3)	20.8(4)
O(004)	7963(2)	6723.3(11)	2289(3)	16.2(4)
O(005)	10440(2)	5474.0(13)	2525(3)	25.2(5)
O(006)	9619(2)	3953.5(13)	2671(3)	25.2(4)
C(007)	5731(3)	4743.6(16)	2539(4)	14.6(5)

Atom	x	y	z	U_{eq}
C(008)	7777(3)	7205.3(16)	4007(4)	18.4(5)
C(009)	6066(3)	5643.1(16)	2439(4)	13.9(5)
C(00A)	8896(3)	5350.6(17)	2538(4)	16.3(5)
C(00B)	7721(3)	5902.3(16)	2490(4)	14.2(5)
C(00C)	11073(3)	6289.2(18)	2343(5)	23.6(6)
C(00D)	8556(3)	4448.2(17)	2611(4)	17.0(6)
C(00E)	6881(3)	4187.5(16)	2614(4)	15.7(5)

Table 2: Anisotropic Displacement Parameters (×10⁴) **O076**. The anisotropic displacement factor exponent takes the form: $-2□^{2}[h^2a^{*2} \times U_{11}+ ... +2hka^{*} \times b^{*} \times U_{12}]$

Atom	U_{11}	U_{22}	U_{33}	U_{23}	U_{13}	U_{12}
Br(01)	14.53(16)	17.29(18)	20.33(16)	-0.73(10)	1.67(10)	-4.12(10)
Br(02)	27.31(18)	10.36(17)	34.83(19)	-0.58(11)	-0.37(13)	-0.24(11)
O(003)	15.3(9)	14.4(10)	32.8(11)	-0.2(8)	1.7(8)	2.5(8)
O(004)	19.0(9)	9.2(9)	20.7(9)	0.9(7)	2.9(7)	-1.3(7)
O(005)	10.9(9)	16.7(11)	47.9(13)	-0.4(9)	2.3(9)	0.8(7)
O(006)	21.3(10)	17(1)	37.0(11)	-1.0(9)	1.7(9)	7.9(9)
C(007)	16.4(12)	13.4(13)	14.1(11)	-0.5(9)	0.9(9)	-2.3(10)
C(008)	21.7(13)	9.6(13)	24.1(13)	-3.1(10)	1.9(10)	0.6(10)
C(009)	14.4(12)	11.5(13)	15.6(11)	-1.2(9)	0.0(9)	-0.9(10)
C(00A)	13.6(12)	14.2(14)	20.9(13)	-1.1(10)	0.3(10)	-0.7(10)
C(00B)	15.2(12)	10.8(13)	16.5(11)	-0.7(9)	0.6(9)	-0.8(10)
C(00C)	12.7(12)	18.5(16)	39.7(17)	0.0(12)	2.2(12)	-2.2(10)
C(00D)	15.5(14)	13.5(14)	22.0(13)	-1.5(10)	0.4(10)	3.1(10)
C(00E)	19.7(13)	8.8(13)	18.4(12)	-1.0(9)	-0.2(10)	-0.9(10)

Table 3: Bond Lengths in Å for **O076**.

Atom	Atom	Length/Å
Br(01)	C(007)	1.873(3)
Br(02)	C(00E)	1.864(3)
O(003)	C(009)	1.212(3)
O(004)	C(008)	1.445(3)
O(004)	C(00B)	1.365(3)
O(005)	C(00A)	1.338(3)
O(005)	C(00C)	1.446(3)

Atom	Atom	Length/Å
O(006)	C(00D)	1.214(3)
C(007)	C(009)	1.498(4)
C(007)	C(00E)	1.336(4)
C(009)	C(00B)	1.476(4)
C(00A)	C(00B)	1.348(4)
C(00A)	C(00D)	1.503(4)
C(00D)	C(00E)	1.496(4)

Table 4: Bond Angles in ° for **O076**.

Atom	Atom	Atom	Angle/°
C(00B)	O(004)	C(008)	115.00(19)
C(00A)	O(005)	C(00C)	121.1(2)
C(009)	C(007)	Br(01)	114.59(19)
C(00E)	C(007)	Br(01)	123.8(2)
C(00E)	C(007)	C(009)	121.6(2)
O(003)	C(009)	C(007)	121.4(2)
O(003)	C(009)	C(00B)	120.8(2)
C(00B)	C(009)	C(007)	117.8(2)
O(005)	C(00A)	C(00B)	129.4(3)
O(005)	C(00A)	C(00D)	110.0(2)

Atom	Atom	Atom	Angle/°
C(00B)	C(00A)	C(00D)	120.6(2)
O(004)	C(00B)	C(009)	115.6(2)
C(00A)	C(00B)	O(004)	122.6(2)
C(00A)	C(00B)	C(009)	121.4(3)
O(006)	C(00D)	C(00A)	120.4(2)
O(006)	C(00D)	C(00E)	121.7(3)
C(00E)	C(00D)	C(00A)	117.8(2)
C(007)	C(00E)	Br(02)	123.0(2)
C(007)	C(00E)	C(00D)	120.6(2)
C(00D)	C(00E)	Br(02)	116.39(19)

Table 5: Torsion Angles in ° for **O076**.

**Atom**	**Atom**	**Atom**	**Atom**	**Angle/°**
Br(01)	C(007)	C(009)	O(003)	-2.7(3)
Br(01)	C(007)	C(009)	C(00B)	178.23(17)
Br(01)	C(007)	C(00E)	Br(02)	0.2(3)
Br(01)	C(007)	C(00E)	C(00D)	-179.90(18)
O(003)	C(009)	C(00B)	O(004)	-2.5(4)
O(003)	C(009)	C(00B)	C(00A)	-176.2(3)
O(005)	C(00A)	C(00B)	O(004)	4.4(4)
O(005)	C(00A)	C(00B)	C(009)	177.6(3)
O(005)	C(00A)	C(00D)	O(006)	0.9(4)
O(005)	C(00A)	C(00D)	C(00E)	-179.4(2)
O(006)	C(00D)	C(00E)	Br(02)	0.2(3)
O(006)	C(00D)	C(00E)	C(007)	-179.7(3)
C(007)	C(009)	C(00B)	O(004)	176.6(2)
C(007)	C(009)	C(00B)	C(00A)	2.9(4)
C(008)	O(004)	C(00B)	C(009)	77.4(3)
C(008)	O(004)	C(00B)	C(00A)	-109.0(3)
C(009)	C(007)	C(00E)	Br(02)	-179.57(19)
C(009)	C(007)	C(00E)	C(00D)	0.3(4)
C(00A)	C(00D)	C(00E)	Br(02)	-179.59(19)
C(00A)	C(00D)	C(00E)	C(007)	0.5(4)
C(00B)	C(00A)	C(00D)	O(006)	-179.3(3)
C(00B)	C(00A)	C(00D)	C(00E)	0.4(4)
C(00C)	O(005)	C(00A)	C(00B)	-3.5(5)
C(00C)	O(005)	C(00A)	C(00D)	176.3(2)
C(00D)	C(00A)	C(00B)	O(004)	-175.4(2)
C(00D)	C(00A)	C(00B)	C(009)	-2.2(4)
C(00E)	C(007)	C(009)	O(003)	177.1(3)
C(00E)	C(007)	C(009)	C(00B)	-2.0(4)

Table 6: Hydrogen Fractional Atomic Coordinates (×10[4]) and Equivalent Isotropic Displacement Parameters (Å[2]×10[3]) for **O076**. U_{eq} is defined as 1/3 of the trace of the orthogonalised U_{ij}.

**Atom**	**x**	**y**	**z**	U_{eq}
H(00A)	8012.63	7768.54	3756.18	28
H(00B)	8477.96	7006.09	5057.56	28
H(00C)	6715.55	7161.56	4347.64	28
H(00D)	10594.01	6543.84	1190.43	35
H(00E)	12185.38	6255.54	2261.98	35
H(00F)	10860.34	6609.39	3454.57	35

10. Cartesian Coordinates

1b$^{o}_{min}$

Atom	x	y	z
C	-15.038.100.939	29.044.368.258	-189.601.034
			-
C	-27.435.818.052	33.607.833.909	22.455.777.594
			-
C	-39.483.644.619	25.473.892.067	19.515.662.397
			-
C	-38.125.147.164	13.217.392.807	10.870.653.103
C	-2.576.252.481	0.8477607128	-0.7641834582
C	-13.509.790.066	15.744.334.641	-1.256.123.951
			-
O	-0.2383080914	10.701.232.635	11.349.367.055
			-
O	-50.459.938.542	28.802.618.984	23.898.102.771
			-
O	-0.4325081838	36.197.832.826	22.979.630.224
			-
C	0.7948298695	36.068.501.912	15.553.350.439
			-
O	-2.818.761.995	44.928.455.524	29.748.998.423
			-
C	-39.449.364.116	53.770.868.513	28.651.658.533
C	-50.842.932.396	0.7028804489	-0.6524457487
C	-23.479.058.148	-0.4412971071	-0.0741639194
C	-14.918.574.954	-0.6393755093	0.9922912281
		-	
S	-14.901.899.315	23.122.883.649	14.790.583.114
		-	
C	-26.387.026.896	27.639.229.878	0.2294284537
		-	
C	-29.935.817.273	16.545.008.209	-0.4902655318
			-
C	-61.044.376.093	0.2791332862	15.684.984.657
C	-72.049.223.768	-0.277954771	-0.9709561582
S	-7.005.242.547	-0.2534301354	0.7742924737
C	-54.365.875.455	0.4948582063	0.6671830293
		-	
C	-30.987.181.919	41.484.777.864	0.0647001844
C	-0.6441714635	0.3443262569	17.451.250.838
C	-46.821.095.016	0.8236038157	19.198.924.874
			-
C	-84.101.279.334	-0.8308376157	16.019.459.192
		-	
C	-22.860.412.204	52.412.126.554	0.4149864988

		-	
C	-27.364.384.881	65.489.214.316	0.2457982109
		-	
C	-40.040.023.534	67.944.416.967	-0.2849181614
		-	
C	-48.212.553.485	57.179.097.126	-0.6375918169
		-	
C	-43.789.617.457	44.094.763.898	-0.4585127408
		-	-
C	-83.591.030.278	13.176.178.816	29.216.306.093
		-	-
C	-9.497.525.022	18.294.484.571	35.387.835.005
	-		-
C	107.110.847.683	-1.882.857.121	28.493.570.635
	-	-	-
C	107.739.515.051	14.136.011.685	15.362.696.475
C	-96.390.531.138	-0.8895972542	-0.9207379426
			-
H	13.074.429.393	45.262.454.292	18.479.294.282
H	0.6092753166	36.220.328.858	-0.4761841457
			-
H	14.008.099.011	27.357.157.635	18.052.892.644
			-
H	-35.747.774.314	63.438.235.029	32.145.844.027
			-
H	-47.760.520.319	50.396.779.586	34.847.652.858
			-
H	-42.810.350.822	54.681.818.263	18.273.184.849
		-	-
H	-36.745.408.446	16.948.162.683	13.320.383.054
			-
H	-60.198.696.384	0.4167866968	26.389.952.116
H	-10.561.703.299	13.545.542.054	16.613.424.732
H	-0.5935361254	0.0933615491	2.810.197.747
H	0.3773905385	0.3715086119	13.503.716.398
H	-395.448.197	16.176.406.824	17.278.834.578
H	-53.554.136.741	11.650.417.704	27.133.608.564
H	-41.290.904.004	-0.0453958374	2.297.640.016
		-	
H	-1.286.808.125	50.636.277.705	0.803819406
		-	
H	-2.090.187.574	73.781.833.972	0.5211265312
		-	
H	-4.353.597.776	78.144.288.417	-0.4189620466
		-	
H	-58.144.081.268	58.965.178.191	-1.041.203.447
		-	
H	-50.354.668.474	35.794.197.657	-0.7027585332
			-
H	-74.148.691.772	-130.864.812	34.579.220.506

H	-9.433.260.732	-21.989.061.367	-45.588.770.773
H	-115.972.021.356	-22.882.190.083	-33.298.671.541
H	-117.126.422.404	-14.461.927.739	-0.9895893764
H	-97.106.405.417	-0.5039002113	0.0927701535

1b$^{c}_{min}$

Atom	x	y	z
C	-99.299.507.049	0.8515276913	0.3104560882
C	-9.799.601.403	-0.3774949452	0.9124215544
C	-85.209.763.459	-11.240.964.334	0.9064322419
C	-74.118.857.808	-0.5779458306	0.0957963348
C	-76.070.160.456	0.6628158584	-0.6655290947
C	-88.175.122.792	14.819.437.539	-0.4335020165
O	-89.522.350.412	26.308.484.045	-0.8741646675
O	-84.208.107.706	-21.557.114.744	15.841.985.308
O	-110.936.788.922	150.449.798	0.5131187105
C	-116.498.259.109	23.821.995.491	-0.4760374544
O	-108.563.504.765	-0.8149636678	16.286.791.853
C	-111.488.222.257	-22.129.752.161	1.763.784.573
C	-61.783.902.309	-12.046.479.283	0.0804706422
C	-66.629.593.781	10.560.956.137	-15.956.823.207
C	-55.232.731.308	0.0887480415	-19.359.982.381
S	-42.585.142.048	11.118.300.076	-28.540.863.606
C	-54.129.965.725	24.453.040.538	-30.443.010.807
C	-65.787.803.241	22.644.501.367	-23.489.638.241
C	-57.906.575.148	-24.575.556.717	0.6408409758
C	-45.539.989.842	-29.120.006.676	0.2658296033
S	-36.948.611.066	-18.146.262.329	-0.8292329373
C	-50.051.265.181	-0.5011870533	-0.6109055868

			-
C	-50.633.409.562	36.042.890.671	38.702.366.783
			-
C	-60.527.400.936	-0.9753659416	29.306.112.916
C	-44.261.803.413	0.5585422611	0.3614765013
		-	
C	-39.348.118.641	41.774.116.552	0.6700137811
			-
C	-37.285.484.284	38.699.153.094	42.291.944.909
			-
C	-34.046.678.488	4.980.131.529	50.052.137.651
			-
C	-44.063.903.743	58.480.317.862	54.425.828.793
			-
C	-57.373.742.475	55.947.414.109	50.989.277.141
C	-60.643.301.434	44.865.669.309	-4.324.762.396
		-	
C	-28.748.098.053	47.395.830.213	-0.0657504085
		-	
C	-23.023.168.947	59.496.611.372	0.31826395
C	-27.727.373.786	-6.622.539.203	144.710.653
C	-38.204.467.474	-6.073.475.399	21.917.603.375
		-	
C	-43.946.850.654	48.651.238.686	1.811.176.367
	-		
H	127.163.469.673	24.242.945.433	-0.2398004384
	-		
H	112.059.646.404	33.761.983.243	-0.4214991621
	-		-
H	115.155.880.529	19.829.136.198	14.861.451.645
	-	-	
H	122.161.054.717	22.508.135.592	1.997.579.188
	-	-	
H	109.603.648.518	27.534.757.167	0.8310202284
	-	-	
H	105.649.357.469	26.660.430.441	25.647.769.676
H	-73.588.158.484	3.009.088.532	-2.293.621.331
		-	
H	-64.712.608.811	30.250.282.302	1.257.319.064
H	-64.606.368.872	-0.4738728787	-3.811.894.655
		-	-
H	-52.528.952.309	16.476.443.136	32.485.045.908
		-	-
H	-6.847.086.337	15.686.491.901	24.689.963.013
H	-35.442.681.451	10.413.373.784	-0.0650127334
H	-51.748.138.542	1.324.813.868	0.5811907574
H	-41.422.081.556	0.072605523	12.983.075.582
			-
H	-29.361.711.056	32.120.130.099	38.834.124.641

			-
H	-23.668.667.817	51.683.555.181	52.657.902.009
			-
H	-41.538.675.912	67.131.852.382	60.493.101.268
			-
H	-65.249.446.414	62.601.097.232	54.417.028.366
			-
H	-71.037.113.941	42.901.508.559	40.828.642.511
		-	
H	-25.090.918.657	42.347.535.409	-0.9555257965
		-	
H	-14.888.083.156	63.684.425.302	-0.267557469
H	-23.244.309.932	-7.565.426.226	17.477.322.014
		-	
H	-41.865.885.379	65.853.613.407	30.773.895.157
		-	
H	-51.923.066.729	44.376.896.015	24.099.693.386

13_{min}

Atom	x	y	z
C	-85.237.231.944	1.845.144.961	4.174.231.402
C	-77.780.346.415	21.896.299.724	30.908.716.649
N	-81.506.540.571	32.977.214.779	23.413.124.614
C	-92.539.323.754	41.020.512.402	25.944.422.176
	-		
C	100.454.531.568	38.176.506.418	36.625.142.974
C	-9.747.192.847	26.479.033.066	45.879.731.578
C	-9.415.863.433	52.244.436.921	15.961.905.634
C	-65.410.991.917	1.501.336.992	25.620.059.942
C	-8.253.060.874	0.7006881917	50.554.962.711
O	-89.462.185.064	0.4373808372	60.250.694.453
O	-71.639.854.711	-0.0488835437	47.130.180.859
		-	
C	-6.876.094.058	11.777.251.462	55.675.284.158
		-	
C	-76.910.744.956	24.020.238.852	51.756.083.409
	-		
C	112.434.406.052	45.749.946.065	405.108.193
	-		
O	119.165.696.212	42.868.234.045	50.276.924.427
	-		
O	115.502.309.254	56.304.935.087	32.401.975.122
	-		
C	127.236.746.206	63.906.951.862	36.039.063.711
	-		
C	139.950.325.138	57.583.863.983	30.559.912.087
H	-7.581.787.103	35.280.268.013	15.418.089.482

H	-96.261.083.171	30.085.798.553	56.184.256.901
	-		
H	106.227.808.678	19.880.074.937	46.474.682.686
H	-93.976.144.385	61.983.642.242	20.902.560.898
H	-86.127.758.779	51.992.431.914	0.8509081237
	-		
H	103.731.392.482	5.154.501.943	10.747.795.022
H	-67.516.544.558	0.4651798938	22.875.529.625
H	-61.560.348.187	2.022.458.262	16.780.265.618
H	-57.517.854.132	14.720.828.541	33.163.175.257
		-	
H	-58.050.166.589	13.528.033.513	54.324.532.253
H	-70.691.217.244	-0.894450874	66.049.045.887
		-	
H	-73.940.140.343	32.602.539.391	57.898.445.528
		-	
H	-87.569.772.832	22.181.141.287	53.341.221.523
		-	
H	-75.271.645.494	26.598.050.961	41.236.787.681
	-		
H	125.512.293.378	73.790.101.728	31.684.226.409
	-		
H	127.663.967.788	64.766.248.819	469.220.384
	-		
H	148.569.275.142	63.971.248.661	32.823.519.076
	-		
H	141.626.825.298	47.796.413.135	35.133.195.294
	-		
H	139.338.937.304	56.372.314.043	19.690.364.998

1b$^{o}_{ADJ}$

Atom	x	y	z
	-		
C	0.80407314	349.775.721	-15.058.936
	-		-
C	207.115.394	392.368.047	179.993.078
	-		-
C	322.183.471	299.959.576	164.907.763
	-		-
C	297.730.862	160.333.084	113.558.157
			-
C	-17.214.937	118.960.893	0.82168213
	-		-
C	0.56292178	214.493.641	0.95931445
			-
O	0.5663618	180.110.869	0.61935797

O	-436.514.137	335.173.676	-192.783.693
O	0.20629407	438.781.774	-158.489.269
C	147.873.499	397.970.545	-212.489.828
O	-223.091.921	521.771.558	-213.226.349
C	-328.437.918	566.789.326	-30.035.809
C	-418.515.763	0.75676409	-0.98184586
C	-140.187.753	-0.20360185	-0.42931895
C	-0.70252086	-0.58842619	0.69755785
S	-0.52539417	-232.353.261	0.74765049
C	-137.936.463	-253.891.817	-0.77136128
C	-177.984.733	-131.898.097	-124.905.733
C	-502.004.666	0.36654462	-20.800.463
C	-607.039.379	-0.44389611	-172.961.582
S	-606.654.767	-0.69825946	0.0080325
C	-463.006.119	0.26218373	0.22656528
C	-159.246.256	-386.514.017	-136.250.269
C	-0.1377374	0.23915978	181.482.925
C	-406.647.122	0.45741426	160.179.287
C	-709.883.527	-104.369.635	-258.867.137
C	-0.70309215	-492.966.732	-11.307.708
C	-0.91739511	-618.006.397	-170.760.516
C	-201.974.625	-639.542.017	-253.634.967
C	-291.298.897	-534.768.885	-27.740.735
C	-27.087.162	-409.958.779	-218.898.606
C	-738.860.876	-0.48460937	-384.860.303
C	-835.602.061	-105.280.301	-467.499.182

C	-906.622.802	-218.367.539	-426.392.498
C	-879.234.545	-274.425.729	-301.557.798
C	-781.825.994	-218.515.259	-219.041.541
H	189.667.285	488.326.595	-257.674.943
H	213.515.316	361.711.466	-133.176.829
H	135.335.041	320.456.288	-288.416.443
H	-294.462.863	664.603.825	-335.324.252
H	-341.559.768	499.315.309	-385.144.501
H	-422.533.101	577.046.671	-245.971.095
H	-229.914.087	-119.545.317	-219.202.403
H	-481.479.699	0.65910474	-310.343.234
H	-0.65281113	12.013.264	188.001.197
H	-0.24486959	-0.26788919	278.022.455
H	0.92429182	0.45131419	165.284.401
H	-342.276.962	134.128.186	162.559.577
H	-486.032.038	0.59726936	23.436.296
H	-346.136.973	-0.40244361	191.524.765
H	0.1722729	-477.009.915	-0.50667087
H	-0.21505712	-698.653.238	-151.387.702
H	-218.573.254	-737.047.608	-298.603.605
H	-378.573.943	-550.778.751	-340.211.729
H	-343.233.254	-330.457.678	-234.440.606
H	-685.948.501	0.40494442	-417.549.138
H	-856.675.645	-0.59878704	-56.401.158
H	-982.593.525	-261.927.032	-490.707.675
H	-933.387.407	-362.568.899	-268.231.005

	-	-	-
H	760.258.714	264.831.274	123.120.406

1b$^{c}_{ADJ}$

Atom	x	y	z
C	-907.836.258	112.179.091	0.17998013
		-	
C	-924.726.447	0.12554757	0.73611198
		-	
C	-815.290.185	113.762.943	0.69431059
		-	-
C	-694.523.697	0.80714238	0.08928182
			-
C	-681.491.236	0.51212176	0.72062855
			-
C	-779.981.716	156.271.355	0.39190023
			-
O	-760.456.436	277.328.926	0.59725088
		-	
O	-828.416.733	220.368.065	130.995.158
	-		
O	1.007.239.579	203.590.213	0.34045185
	-		-
C	1.058.567.529	263.598.508	0.85540958
	-	-	
O	1.041.569.911	0.33691453	13.524.679
	-	-	
C	1.081.948.342	157.197.172	196.805.019
		-	-
C	-590.794.071	172.138.538	0.19599324
			-
C	-579.357.693	0.75600729	161.673.319
C	-493.130.236	-0.4216981	-20.809.625
			-
S	-344.228.685	0.34925571	290.484.382
			-
C	-423.119.303	193.936.034	293.689.147
			-
C	-540.923.105	197.989.552	224.007.228
		-	
C	-584.738.595	308.113.521	0.22444285
		-	-
C	-475.985.164	378.697.737	0.22381751
		-	-
S	-365.035.195	283.206.065	121.713.654
		-	
C	-459.226.653	126.455.115	-0.8373204

C	-360.215.613	305.280.399	-365.222.264
C	-570.017.456	-120.381.636	-317.548.716
C	-377.246.005	-0.49734521	0.23249255
C	-447.822.781	-52.011.553	0.03734703
C	-223.998.303	301.838.564	-400.493.006
C	-164.946.124	408.484.218	-467.827.892
C	-240.654.289	520.751.804	-501.701.297
C	-376.183.993	525.442.011	-467.827.806
C	-435.486.172	419.087.662	-400.629.632
C	-36.131.599	-593.889.001	-0.79250987
C	-336.095.349	-728.509.037	-0.54132164
C	-396.325.993	-79.214.929	0.54553596
C	-481.979.987	-720.023.733	138.193.985
C	-507.446.994	-585.533.758	113.410.649
H	-1.136.975.463	332.072.638	-0.52664653
H	-980.766.858	318.808.191	-138.854.447
H	-1.102.407.398	18.720.126	-151.003.744
H	-1.186.766.634	-140.505.111	222.473.308
H	-1.072.259.874	-242.219.114	129.185.023
H	-1.022.808.837	-174.851.473	286.923.586
H	-598.144.904	288.418.513	-209.069.091
H	-664.793.646	-352.937.683	0.79279585
H	-596.104.963	-0.52242689	-39.891.812
H	-509.090.056	-20.149.577	-358.014.319
H	-662.284.986	-162.758.443	-276.815.284
H	-279.703.169	-0.20208691	-0.16000492

H	-430.955.962	0.40000828	0.55251205
		-	
H	-362.234.795	114.084.185	110.288.635
			-
H	-163.453.439	215.743.499	373.562.874
			-
H	-0.59520121	403.886.562	493.651.851
			-
H	-194.638.749	603.843.238	554.438.435
			-
H	-436.093.433	612.042.707	494.586.719
			-
H	-541.306.769	423.028.094	376.914.255
		-	
H	-31.507.692	545.957.671	-16.508.906
		-	-
H	-269.511.505	783.846.986	119.756.157
		-	
H	-376.409.642	897.116.423	0.74266624
		-	
H	-528.469.006	768.558.584	223.549.721
		-	
H	-572.164.005	529.867.693	180.416.326

13_{ADJ} ($1b^{o}_{ADJ}$)

Atom	x	y	z
	-		-
C	332.523.653	112.640.897	595.218.231
	-	-	-
C	373.679.825	0.05903205	647.753.197
	-	-	-
N	281.185.753	109.658.568	655.723.204
	-	-	-
C	163.430.425	114.346.965	581.132.136
	-		-
C	117.258.628	0.01880182	527.598.399
	-		-
C	185.112.604	132.838.022	564.276.888
			-
C	-10.575.818	-25.313.566	569.928.861
	-	-	-
C	509.435.731	0.43450916	701.053.062
	-		-
C	416.649.765	231.097.563	572.761.441
	-		-
O	369.721.778	341.967.111	552.139.705
	-		-
O	551.085.145	207.932.769	572.213.859

C	-635.699.566	322.903.665	-546.312.813
C	-657.820.609	406.611.306	-671.398.715
C	-0.01163158	0.15642699	-438.089.312
O	0.40416624	12.458.593	-401.017.209
O	0.54063086	-101.253.475	-397.261.373
C	164.752.222	-0.91976797	-30.400.643
C	29.672.539	-0.68045628	-375.832.946
H	-31.534.672	-197.763.282	-691.418.331
H	-17.346.154	204.255.236	-482.634.193
H	-134.154.586	179.646.156	-650.158.122
H	-133.390.529	-312.336.261	-658.073.629
H	0.02650442	-250.818.585	-561.705.431
H	-14.418.571	-304.882.361	-4.811.661
H	-56.832.203	-0.96469009	-625.179.527
H	-566.121.351	0.44195252	-731.506.027
H	-498.097.224	-110.063.647	-78.751.919
H	-729.266.496	279.159.863	-51.058.124
H	-590.865.325	381.569.159	-465.878.746
H	-727.893.207	488.016.367	-649.451.136
H	-563.755.246	450.516.034	-705.620.158
H	-700.271.806	345.970.049	-752.163.472
H	16.386.812	-188.577.485	-25.295.147
H	143.568.847	-0.13009585	-231.640.933
H	378.975.454	-0.69125868	-303.358.868
H	296.323.201	0.29197779	-425.811.232

		-	-
H	315.701.375	146.136.803	450.294.775

13_{ADJ} ($1b^{c}_{ADJ}$)

Atom	x	y	z
C	-858.894.485	177.736.855	414.837.596
C	-781.522.972	215.470.864	309.330.355
N	-823.806.398	319.812.407	228.353.399
C	-93.690.116	397.307.762	250.985.741
	-		
C	1.016.354.515	368.846.977	35.762.785
C	-993.018.507	244.137.926	441.715.315
C	-951.506.933	507.879.554	149.463.994
C	-647.450.968	159.199.649	268.090.001
C	-827.513.523	0.68962655	508.256.571
O	-901.981.727	0.35654175	599.322.778
O	-70.948.059	0.04373206	483.870.319
		-	
C	-680.436.919	109.732.804	567.294.204
		-	
C	-753.608.305	233.409.362	517.005.536
	-		
C	1.133.692.055	446.333.645	400.078.054
	-		
O	1.211.088.197	407.149.093	486.119.307
	-		
O	1.148.566.381	568.040.598	339.311.403
	-		
C	1.263.070.463	645.674.499	38.033.826
C	-138.897.768	604.920.765	304.968.868
H	-778.842.975	330.174.612	138.307.863
	-		
H	1.001.029.288	269.037.575	548.164.277
	-		
H	1.074.693.359	172.189.025	425.109.012
H	-897.268.742	597.763.854	181.646.125
H	-909.516.158	475.374.795	0.53494787
H	-105.559.387	535.855.672	135.538.856
H	-651.160.781	0.50857605	255.757.661
H	-614.059.746	204.670.668	174.160.888
H	-571.780.764	179.506.668	34.454.252
		-	
H	-571.968.373	121.904.003	55.992.159
H	-707.021.525	-0.8699352	670.874.972
		-	
H	-71.935.725	322.436.714	571.111.588

		-	
H	-86.129.623	223.066.138	533.041.365
		-	
H	-735.052.991	248.177.418	410.031.823
	-		
H	1.235.048.877	749.065.827	35.801.003
H	-127.681.889	634.311.073	488.186.614
	-		
H	1.472.403.035	670.316.463	333.093.045
	-		
H	1.416.331.016	501.899.827	329.486.782
	-		
H	1.374.128.279	612.931.826	196.651.967

$(1b^{o}_{min}\text{-}13_{min})_{ADJ}$

Atom	x	y	z
			-
C	-0.8040730809	34.977.569.396	15.058.934.855
	-		-
C	20.711.537.808	39.236.801.652	17.999.306.454
	-		-
C	32.218.344.697	29.995.955.349	16.490.775.014
	-		-
C	29.773.083.957	16.033.307.134	11.355.814.853
	-		
C	17.214.935.722	11.896.088.458	-0.8216820681
C	-0.5629217323	21.449.362.409	-0.9593143737
O	0.5663617609	18.011.085.546	-0.6193579244
	-		-
O	43.651.410.342	33.517.365.036	19.278.367.776
			-
O	0.2062940576	43.878.173.992	15.848.925.724
			-
C	1.478.734.882	39.797.051.467	21.248.981.172
			-
O	-223.091.904	52.177.151.798	21.322.633.273
	-		-
C	32.843.789.287	56.678.928.214	30.035.806.748
	-		
C	41.851.573.121	0.7567640295	-0.9818457857
	-		
C	14.018.774.251	-0.2036018311	-0.4293189171
C	-0.7025208105	-0.5884261487	0.6975577944
		-	
S	-0.5253941271	23.235.324.344	0.7476504392
	-	-	
C	13.793.645.274	25.389.179.782	-0.7713612234

C	- 17.798.471.965	- 13.189.808.687	- 12.490.572.345
C	- 50.200.462.738	0.3665445912	- 20.800.461.481
C	- 60.703.933.248	-0.4438960709	- 17.296.156.837
S	- 60.665.472.163	-0.6982593997	0.0080324951
C	- 46.300.608.387	0.2621837112	0.2265652618
C	- 15.924.624.418	- 38.651.398.723	- 13.625.025.886
C	-0.1377373857	0.2391597656	18.148.291.076
C	-4.066.470.907	0.4574142266	16.017.927.455
C	- 70.988.347.151	- 10.436.962.684	- 25.886.711.704
C	-0.70309209	- 49.296.669.463	- 11.307.707.087
C	-0.9173950396	- 61.800.634.899	- 17.076.050.223
C	- 20.197.460.925	- 63.954.196.599	- 25.363.494.772
C	- 29.129.887.545	- 53.476.884.364	- 27.740.732.856
C	- 27.087.159.951	- 40.995.874.779	- 21.889.858.924
C	-7.388.608.182	-0.4846093346	- 38.486.027.359
C	- 83.560.199.847	- 10.528.029.292	- 46.749.914.647
C	- 90.662.273.342	- 21.836.752.202	- 42.639.246.544
C	- 87.923.447.993	- 27.442.570.806	- 30.155.777.422
C	- 78.182.593.671	- 21.851.524.226	- 21.904.152.376
H	18.966.727.034	48.832.655.765	- 25.767.492.377
H	21.351.529.916	36.171.143.877	- 13.317.681.896
H	13.533.503.084	32.045.626.319	- 28.841.642.061
H	- 29.446.284.073	66.460.377.151	- 33.532.422.633
H	- 34.155.974.254	49.931.527.092	-3.851.444.711
H	- 42.253.306.873	57.704.662.793	- 24.597.107.658
H	- 22.991.406.982	- 11.954.530.753	- 21.920.238.583

H	-48.147.966.225	0.6591046902	-31.034.320.972
H	-0.6528110757	12.013.263.058	1.880.011.825
H	-0.2448695742	-0.2678891665	27.802.243.357
H	0.9242917507	0.4513141603	16.528.438.905
H	-34.227.693.644	13.412.817.565	16.255.956.418
H	-48.603.200.133	0.5972693176	23.436.294.175
H	-34.613.694.635	-0.4024435798	19.152.475.027
H	0.1722728827	-4.770.098.783	-0.5066708346
H	-0.2150571082	-69.865.318.642	-15.138.768.998
H	-21.857.323.743	-73.704.755.151	-29.860.358.181
H	-37.857.391.414	-550.778.711	-34.021.170.262
H	-34.323.322.744	-3.304.576.528	-23.444.058.834
H	-68.594.844.648	0.4049443936	-41.754.910.626
H	-85.667.557.943	-0.5987869915	-56.401.153.734
H	-98.259.344.754	-26.192.701.153	-49.070.763.801
H	-93.338.733.456	-36.256.887.201	-26.823.098.438
H	-76.025.865.778	-26.483.125.433	-12.312.039.622
C	-33.252.362.744	11.264.088.874	-59.521.818.802
C	-37.367.979.609	-0.0590320503	-64.775.314.575
N	-28.118.573.178	-10.965.855.975	-65.572.315.158
C	-16.343.041.307	-11.434.695.599	-58.113.209.227
C	-11.725.861.921	0.0188018177	-52.759.835.895
C	-18.511.258.988	13.283.801.206	-56.427.684.228
C	-10.575.817.171	-25.313.564.093	-56.992.881.811
C	-50.943.569.166	-0.4345091282	-70.105.300.727
C	-41.664.973.301	2.310.975.455	-57.276.139.459
O	-36.972.175.025	34.196.708.509	-55.213.966.194

O	- 55.108.510.385	20.793.275.293	- 57.221.381.462
C	- 63.569.951.875	32.290.363.991	- 54.631.277.064
C	- 65.782.055.933	40.661.127.557	67.139.866.566
C	-0.0116315752	0.156426976	- 43.808.927.846
O	0.4041662104	12.458.592.013	- 40.101.717.818
O	0.5406308188	- 10.125.346.773	- 39.726.134.272
C	16.475.220.919	-0.9197679003	- 30.400.640.635
C	29.672.536.738	-0.6804562234	- 37.583.291.731
H	- 31.534.669.658	- 19.776.326.721	- 69.141.827.849
H	- 17.346.152.667	2.042.552.206	- 48.263.415.644
H	- 13.415.457.544	17.964.614.246	-6.501.580.742
H	- 13.339.051.843	- 31.233.623.744	- 65.807.357.874
H	0.0265044204	- 25.081.856.597	- 56.170.538.846
H	- 14.418.569.918	- 30.488.233.777	-4.811.660.635
H	- 56.832.198.695	-0.9646900149	- 62.517.947.962
H	- 56.612.130.977	0.4419524839	- 73.150.596.965
H	- 49.809.718.578	- 11.006.363.899	- 78.751.913.102
H	-7.292.664.396	27.915.984.229	- 51.058.120.114
H	- 59.086.528.017	38.156.913.048	-4.658.787.101
H	- 72.789.315.292	48.801.632.997	- 64.945.108.701
H	- 56.375.520.202	45.051.599.964	- 70.562.010.413
H	- 70.027.175.365	34.597.002.253	- 75.216.341.675
H	16.386.810.753	- 18.857.747.115	- 25.295.145.088
H	14.356.883.655	-0.1300958435	- 23.164.091.522
H	37.897.542.462	-0.6912586317	- 30.335.884.474

			-
H	29.632.317.837	0.2919777636	42.581.119.928
		-	-
H	31.570.135.069	14.613.679.192	45.029.474.075

($1b^{c}_{min}$-13_{min})$_{ADJ}$

Atom	x	y	z
C	-97.004.469.697	11.576.897.879	0.3124094828
C	-96.367.747.974	-0.0725992805	0.9032082039
C	-8.369.992.332	-0.853820236	0.9330678212
C	-72.665.719.045	-0.3882003749	0.0780252081
C	-7.409.501.795	0.8596594826	-0.6882739918
C	-8.564.090.081	17.411.407.164	-0.4306143476
O	-86.524.835.066	29.073.832.319	-0.8495768174
		-	
O	-82.940.963.455	18.371.150.135	16.861.499.779
	-		
O	108.219.976.372	19.147.453.412	0.4726162908
	-		
C	115.891.298.624	21.544.497.797	-0.7216801132
	-		
O	107.147.534.103	-0.4916692931	15.997.650.728
	-	-	
C	110.869.669.311	18.845.739.351	15.957.561.444
		-	
C	-60.748.234.533	10.960.795.727	0.01895636
			-
C	-64.789.340.304	11.826.314.148	16.601.043.835
			-
C	-5.421.130.263	0.1402566161	20.359.669.844
			-
S	-41.297.769.162	10.738.866.989	30.110.471.758
			-
C	-52.037.908.655	24.744.741.388	31.745.977.417
			-
C	-63.511.412.018	23.741.181.215	24.309.623.681
		-	
C	-57.507.387.119	23.689.069.234	0.5670555965
		-	
C	-45.620.392.621	29.091.894.153	0.1421003401
S	-36.796.372.163	-1.874.271.102	-0.9886767604
C	-48.895.326.143	-0.476415182	-0.7274773279
C	-48.165.168.158	35.974.742.122	-4.032.173.842
			-
C	-60.592.222.038	-0.8917054556	30.008.531.479
C	-41.989.833.546	0.5473818614	0.2109441763

C	-40.129.699.057	-42.115.712.698	0.5265194477
C	-34.763.226.776	37.924.129.352	-4.418.592.111
C	-31.164.928.337	48.694.841.887	-52.256.577.912
C	-4.087.272.242	57.715.777.437	-56.670.343.625
C	-54.232.315.032	55.873.157.682	-52.966.083.396
C	-5.786.549.858	45.129.565.057	-44.903.012.248
C	-30.136.234.073	-48.381.848.144	-0.2433855735
C	-25.050.565.471	-60.814.738.589	0.1252635883
C	-29.803.084.036	-67.227.899.626	12.716.118.372
C	-39.679.088.423	-61.100.180.832	20.494.878.015
C	-44.789.399.459	-48.684.143.827	16.843.956.226
H	-124.307.618.151	27.780.642.093	-0.4158762314
H	-109.932.916.666	26.775.153.939	-14.727.876.097
H	-119.636.878.949	1.208.001.244	-1.128.463.601
H	-121.623.736.903	-18.907.047.669	17.860.717.567
H	-108.873.510.952	-23.413.740.794	0.6223539253
H	-105.591.290.922	-24.338.067.834	23.742.915.974
H	-70.815.994.911	31.664.738.293	-23.635.040.018
H	-64.367.757.693	-28.939.216.187	12.136.070.044
H	-64.733.590.783	-0.3700796361	-3.867.261.622
H	-53.173.682.679	-16.139.317.323	-33.483.440.438
H	-68.695.688.315	-14.326.682.549	-25.039.706.133
H	-33.031.676.691	0.9629042898	-0.2551566537
H	-488.168.138	13.673.305.686	0.4502036474
H	-39.127.706.277	0.0518516357	11.420.295.489
H	-2.706.781.957	31.091.153.679	-40.704.632.918
H	-2.076.336.724	50.051.372.562	-55.071.376.252

			-
H	-38.067.526.401	66.099.497.428	62.980.338.718
			-
H	-61.856.075.886	627.871.462	56.436.601.848
			-
H	-68.300.295.821	43.687.346.478	42.302.489.529
		-	-
H	-2.644.520.752	43.599.827.098	11.463.270.595
		-	
H	-17.390.068.173	65.503.997.801	-0.4852364572
		-	
H	-25.819.808.907	76.912.723.172	15.598.373.443
		-	
H	-4.335.436.419	65.978.014.902	29.475.783.977
		-	
H	-52.275.727.006	43.938.796.309	23.100.092.374
C	-85.025.823.675	0.9349295655	45.679.407.988
C	-720.514.493	0.9566062251	4.148.707.563
N	-67.477.167.587	20.567.597.688	3.443.278.669
C	-75.195.791.971	31.362.451.098	30.456.849.665
C	-88.270.631.765	31.869.035.338	34.282.083.811
C	-94.401.494.038	21.016.344.697	4.299.212.831
C	-67.667.221.216	41.270.199.193	21.916.569.567
C	-61.561.946.349	-0.1117538286	43.352.947.644
C	-91.329.402.013	-0.1626215751	5.306.346.888
	-		
O	102.986.234.548	-0.1145608577	56.894.242.183
		-	
O	-83.382.256.494	12.379.752.313	55.439.371.736
		-	
C	-89.177.397.186	23.329.784.286	6.292.522.427
		-	
C	-96.646.973.597	32.979.682.576	5.384.494.069
C	-97.718.661.345	42.475.333.545	30.663.028.629
	-		
O	109.264.794.642	42.712.344.255	34.830.126.347
O	-92.819.446.205	52.049.143.658	22.384.149.345
	-		
C	101.877.742.957	62.628.430.607	18.448.628.039
C	-11.037.548.424	58.502.094.106	0.6524218138
H	-57.832.499.964	20.441.226.771	31.453.698.935
H	-97.865.377.763	25.331.047.675	52.507.439.849
	-		
H	103.567.064.772	17.288.582.038	38.242.552.097
H	-56.987.949.616	38.882.822.947	21.752.394.156
H	-71.410.269.223	4.107.993.249	11.636.339.144
H	-68.900.947.925	51.452.379.185	25.624.565.058
		-	
H	-64.169.942.371	10.059.449.429	37.613.235.534

H	-51.789.861.098	0.2446786852	39.955.487.063
H	-60.741.757.548	-0.4102206473	5.381.111.147
H	-80.608.510.431	-28.181.845.165	67.660.348.125
H	-95.730.497.734	-19.265.791.347	70.659.693.532
H	-100.212.992.493	-41.547.700.352	59.673.798.658
H	-105.294.357.154	-28.084.179.423	49.273.557.462
H	-9.009.190.832	-36.712.830.961	45.907.037.049
H	-95.305.394.883	70.986.113.547	15.932.510.072
H	-108.073.035.747	65.383.028.373	27.012.559.398
H	-116.309.603.177	67.036.002.943	0.3053831617
H	-117.205.633.769	50.414.174.382	0.9266289814
H	-104.036.021.902	55.086.211.599	-0.1720667971

$(1b^{o}_{min}\text{-}13_{min})_{nonADJ}$

Atom	x	y	z
C	-150.381.032	290.443.727	-189.601.063
C	-274.358.222	336.078.391	-22.455.781
C	-394.836.507	254.738.959	-195.156.654
C	-38.125.153	132.173.948	-108.706.548
C	-257.625.287	0.84776084	-0.76418358
C	-135.097.921	15.744.337	-125.612.414
O	-0.23830813	107.012.343	-113.493.688
O	-504.599.462	288.026.234	-238.981.064
O	-0.43250825	361.978.384	-229.796.337
C	0.79482999	360.685.073	-155.533.528
O	-281.876.242	449.284.623	-29.749.003
C	-394.493.701	537.708.766	-286.516.629

C	-508.429.402	0.70288055	-<br>0.65244585
C	-234.790.618	-0.44129717	-<br>0.07416394
C	-149.185.772	-0.63937561	0.99229139
S	-149.019.016	-231.228.872	147.905.853
C	-263.870.309	-276.392.341	0.22942849
C	-299.358.219	-165.450.108	-<br>0.49026561
C	-610.443.856	0.27913333	-<br>156.849.871
C	-720.492.346	-0.27795481	-0.9709563
S	-700.524.361	-0.25343017	0.77429259
C	-543.658.841	0.49485828	0.66718313
C	-309.871.867	-414.847.842	0.0647002
C	-0.64417156	0.34432631	174.512.535
C	-468.211.022	0.82360395	191.989.278
C	-841.012.919	-0.83083775	-<br>160.194.616
C	-228.604.157	-524.121.345	0.41498656
C	-273.643.891	-654.892.248	0.24579825
C	-400.400.296	-679.444.273	-0.2849182
C	-482.125.609	-571.791.057	-<br>0.63759192
C	-437.896.242	-440.947.707	-<br>0.45851282
C	-835.910.428	-131.761.808	-<br>292.163.105
C	-949.752.647	-182.944.874	-<br>353.878.404
C	-<br>1.071.108.643	-188.285.742	-28.493.575
C	-<br>1.077.395.317	-141.360.139	-<br>153.626.988
C	-963.905.461	-0.88959739	-<br>0.92073808
H	130.744.314	452.624.612	-<br>184.792.971
H	0.60927541	362.203.344	-<br>0.47618422
H	140.081.012	273.571.618	-<br>180.528.954
H	-357.477.797	634.382.449	-32.145.849
H	-477.605.276	503.967.872	-<br>348.476.582
H	-428.103.574	546.818.267	-<br>182.731.876
H	-36.745.414	-169.481.653	-<br>133.203.851

H	-601.987.059	0.41678676	-<br>263.899.562
H	-105.617.049	135.455.441	166.134.273
H	-0.59353621	0.09336157	281.019.817
H	0.3773906	0.37150867	135.037.185
H	-395.448.257	161.764.093	172.788.372
H	-535.541.452	116.504.195	271.336.127
H	-412.909.103	-0.04539584	229.764.036
H	-128.680.832	-506.362.855	0.80381953
H	-209.018.789	-737.818.455	0.52112661
H	-435.359.844	-781.443.002	-<br>0.41896211
H	-5.814.409	-589.651.868	-10.412.036
H	-503.546.762	-357.942.031	-<br>0.70275864
H	-741.487.035	-130.864.832	-<br>345.792.257
H	-943.326.218	-219.890.647	-<br>455.887.778
H	-115.972.039	-228.821.935	-<br>332.986.766
H	-<br>1.171.264.402	-1.446.193	-<br>0.98958952
H	-971.064.202	-0.50390029	0.09277017
C	-561.317.887	-<br>1.086.924.755	-<br>405.491.909
C	-649.644.335	-<br>1.156.754.287	-32.929.363
N	-785.721.933	-<br>1.136.871.922	-<br>348.609.297
C	-841.606.419	-<br>1.048.735.118	-<br>440.213.197
C	-759.285.583	-975.567.009	-<br>519.908.751
C	-607.954.816	-988.537.863	-<br>511.651.385
C	-992.649.793	-<br>1.047.366.237	-<br>437.219.737
C	-618.963.258	-<br>1.257.796.692	-22.120.263
C	-415.035.688	-<br>1.097.333.695	-<br>396.012.963
O	-339.657.846	-<br>1.033.274.349	-<br>467.520.235
O	-369.206.577	-<br>1.184.415.448	-<br>301.309.165
C	-225.656.075	-119.658.286	-<br>290.412.321
C	-166.576.065	-<br>1.087.907.934	-<br>201.719.937

C	-803.846.223	-878.473.348	-620.826.853
O	-725.926.414	-81.590.553	-69.091.901
O	-939.222.437	-863.326.869	-630.886.068
C	-985.351.482	-768.452.879	-729.590.945
C	-985.838.341	-626.366.544	-675.047.317
H	-848.904.072	-1.189.708.616	-290.515.712
H	-56.755.878	-1.017.407.975	-609.633.506
H	-56.311.754	-889.992.679	-493.347.424
H	-1.034.344.335	-1.074.173.966	-534.546.323
H	-1.030.586.417	-1.118.127.695	-362.627.927
H	-1.030.794.418	-94.791.679	-413.008.545
H	-560.360.417	-1.212.988.643	-140.637.472
H	-711.499.747	-1.298.079.125	-178.459.871
H	-560.005.026	-1.340.960.733	-26.039.769
H	-210.136.873	-1.295.903.513	-247.335.526
H	-182.170.495	-1.193.317.242	-390.586.557
H	-0.58922283	-1.104.441.396	-189.141.773
H	-181.199.148	-989.492.733	-247.014.512
H	-213.229.199	-1.088.741.304	-102.616.311
H	-1.086.515.322	-801.999.259	-754.127.073
H	-922.097.301	-776.136.992	-818.337.049
H	-1.028.436.872	-557.995.153	-749.422.116
H	-883.941.002	-593.689.516	-6.526.615
H	-1.046.149.766	-619.746.716	-583.846.524

$(1b^{c}_{min}\text{-}13_{min})_{nonADJ}$

Atom	x	y	z
C	-957.100.204	-174.607.876	-0.17104519
C	-944.065.278	-297.510.169	0.4309204
C	-81.620.274	-372.170.334	0.42493109
C	-705.293.655	-317.555.261	-0.385705
			-
C	-724.806.685	-193.479.065	114.703.061
C	-845.856.336	-111.566.256	-0.91500347
			-
O	-859.328.612	0.03324235	135.566.623
O	-806.186.182	-475.331.862	110.269.755
O	-107.347.305	-109.310.834	0.03161747
	-		
C	1.129.087.763	-0.21540657	-0.95753891
	-		
O	1.049.740.201	-341.257.051	114.717.821
	-		
C	1.078.987.389	-481.058.238	128.228.363
C	-581.944.068	-380.225.486	-0.40103069
			-
C	-630.400.998	-15.415.108	207.718.404
			-
C	-516.432.348	-250.885.859	241.750.003
			-
S	-389.956.426	-148.577.639	333.558.838
			-
C	-505.404.691	-0.15230204	352.580.314
			-
C	-62.198.309	-0.33315601	283.046.571
C	-543.170.791	-505.516.288	0.15933976
C	-41.950.491	-550.960.799	-0.2156717
			-
S	-333.591.104	-441.223.331	131.073.448
			-
C	-464.617.673	-309.879.382	109.240.709
			-
C	-470.439.119	100.668.324	435.173.892
			-
C	-569.379.051	-357.297.281	341.211.332
C	-406.723.043	-203.906.426	-0.12002477
C	-357.586.186	-677.501.928	0.18851257
			-
C	-336.959.836	127.230.955	471.069.682
			-
C	-30.457.177	238.252.602	548.671.626
			-
C	-404.744.046	325.042.648	592.408.549

C	-537.842.463	299.713.607	-558.043.021
C	-57.053.806	188.896.131	-480.626.475
C	-251.585.954	-733.719.078	-0.54725178
C	-19.433.665	-854.726.913	-0.16323733
C	-241.378.708	-922.014.737	0.96560551
C	-346.149.669	-867.108.343	171.025.948
C	-403.573.515	-746.273.163	132.967.542
H	-1.235.739.898	-0.17331155	-0.72130185
H	-108.470.163	0.77859244	-0.90300062
H	-1.115.663.976	-0.61469258	-196.764.686
H	-1.185.715.735	-484.842.073	15.160.783
H	-1.060.141.641	-535.108.299	0.34951907
H	-1.020.598.728	-52.636.503	20.832.762
H	-699.986.658	0.41148257	-277.512.321
H	-611.231.145	-562.263.559	0.775818
H	-610.168.748	-307.147.964	-429.339.689
H	-489.394.551	-424.525.135	-373.000.668
H	-6.488.137	-416.625.621	-295.049.822
H	-318.531.804	-155.626.904	-0.5465141
H	-481.586.412	-127.279.249	0.09968954
H	-378.325.818	-252.500.112	0.81680649
H	-257.722.085	0.61440709	-43.649.147
H	-200.791.639	257.075.004	-574.729.276
H	-379.491.762	411.558.014	-653.081.281
H	-616.599.521	366.250.448	-592.320.546
H	-674.476.214	169.254.519	-456.436.654
H	-215.014.153	-683.236.119	-143.702.737
H	-112.985.774	-896.605.069	-0.74905889
H	-19.654.806	-101.630.346	126.623.124
H	-382.763.857	-918.296.949	259.588.887
H	-483.335.694	-703.529.728	192.846.854
C	-989.601.213	-600.006.919	-281.024.455

C	-871.306.731	-58.577.846	-346.506.305
N	-850.734.682	-472.855.385	-424.677.198
C	-942.933.701	-370.807.277	-44.385.983
C	-1.063.443.876	-378.291.396	-381.386.135
C	-1.099.199.386	-495.029.743	-290.660.542
C	-893.005.078	-261.755.667	-535.728.306
C	-753.403.595	-6.802.947	-346.195.893
C	-1.025.445.177	-71.389.156	-195.337.933
O	-1.132.697.484	-72.170.108	-13.759.137
O	-929.667.807	-810.656.722	-184.588.513
C	-96.255.297	-923.470.487	-100.534.027
C	-1.042.893.703	-1.028.368.744	-17.609.031
C	-1.170.641.863	-278.375.539	-39.246.359
O	-1.276.912.913	-288.994.582	-333.363.325
O	-1.142.726.747	-172.178.986	-473.706.218
C	-1.246.653.064	-0.72580841	-486.000.422
C	-1.349.025.509	-110.954.048	-591.883.159
H	-762.270.555	-465.070.944	-472.343.384
H	-1.124.360.004	-458.008.498	-190.350.061
H	-119.248.225	-541.645.216	-325.038.955
H	-892.536.074	-164.824.516	-485.392.551
H	-791.184.106	-2.834.904	-569.939.463
H	-957.389.057	-251.348.493	-623.351.263
H	-781.345.058	-778.534.605	-384.938.585
H	-671.856.172	-640.678.688	-407.784.937
H	-71.563.257	-696.065.635	-244.937.745

H	-865.530.532	-96.281.713	-0.68918552
H	-1.017.380.493	-88.764.042	-0.13088681
H	-1.059.487.238	-1.115.863.557	-1.121.355
H	-1.140.261.443	-988.167.576	-205.319.993
H	-989.525.479	-106.117.433	-265.947.784
H	-1.193.340.358	0.1886951	-513.497.589
H	-1.294.253.152	-0.58894763	-388.620.073
H	-1.422.041.581	-0.30100494	-604.222.143
H	-1.402.635.618	-201.476.413	-562.157.087
H	-1.300.556.404	-128.453.987	-68.854.429

11. References

1. Fröhlich, J.; Hametner, C.; Kalt, W., Synthesis of trisubstituted thiophenesvia a halogen dance reaction at 2-bromo-5-methylthiophene. *Monatshefte für Chemie / Chemical Monthly* **1996,** *127* (3), 325-330.
2. Wang, R.; Pu, S.; Liu, G.; Cui, S.; Liu, W., Synthesis and photochromism of isomeric unsymmetrical diarylethenes bearing both naphthalene and thiophene moieties. *Journal of Photochemistry and Photobiology A: Chemistry* **2012,** *243*, 47-55.
3. Gregg, C.; Kyryakov, P.; Titorenko, V. I., Purification of Mitochondria from Yeast Cells. **2009,** (30), e1417.
4. Bradford, M. M., A rapid and sensitive method for the quantitation of microgram quantities of protein utilizing the principle of protein-dye binding. *Anal Biochem* **1976,** *72* (1), 248-254.
5. Reddy, C. A.; Beveridge, T. J.; Breznak, J. A.; Marzluf, G. A.; Schmidt, T. M.; Snyder, L. R., *Methods for General and Molecular Microbiology, Third Edition*. American Society of Microbiology: 2007.
6. (a) Becke, A. D., Density-functional thermochemistry. III. The role of exact exchange. *The Journal of Chemical Physics* **1993,** *98* (7), 5648-5652; (b) Lee, C.; Yang, W.; Parr, R. G., Development of the Colle-Salvetti correlation-energy formula into a functional of the electron density. *Physical Review B* **1988,** *37* (2), 785-789; (c) Stephens, P. J.; Devlin, F. J.; Chabalowski, C. F.; Frisch, M. J., Ab Initio Calculation of Vibrational Absorption and Circular Dichroism Spectra Using Density Functional Force Fields. *The Journal of Physical Chemistry* **1994,** *98* (45), 11623-11627.
7. Kuhn, B.; Kollman, P. A., A Ligand That Is Predicted to Bind Better to Avidin than Biotin: Insights from Computational Fluorine Scanning. *Journal of the American Chemical Society* **2000,** *122* (16), 3909-3916.
8. (a) Dolomanov, O. V.; Bourhis, L. J.; Gildea, R. J.; Howard, J. A. K.; Puschmann, H., OLEX2: a complete structure solution, refinement and analysis program. *Journal of Applied Crystallography* **2009,** *42* (2), 339-341; (b) Sheldrick, G. M., Crystal structure refinement with SHELXL. *Acta Crystallographica. Section C, Structural Chemistry* **2015,** *71* (Pt 1), 3-8; (c) Linden, A., Chemistry and structure in Acta Crystallographica Section C. *Acta Crystallographica Section C* **2015,** *71* (1), 1-2.
